# A family of ribosome hibernation factors widespread in Archaea

**DOI:** 10.1038/s41467-026-72341-8

**Published:** 2026-04-27

**Authors:** Clément Madru, Gabrielle Bourgeois, Rémi Dulermo, Régine Capeyrou, Gwendoline Joncour, Karima Figuigui, Magalie Duchateau, Julia Chamot-Rooke, Claire Duboc, Stéphane l’Haridon, Logan Mc Teer, Marta Kwapisz, Béatrice Clouet-d’Orval, Marie Bouvier, Yves Mechulam, Guillaume Borrel, Emmanuelle Schmitt, Didier Flament

**Affiliations:** 1 Structural Biology of the Cell, BIOC, CNRS, Ecole Polytechnique, Institut Polytechnique de Paris, 91120, Palaiseau, France; 2 Univ Brest, Ifremer, Biology and Ecology of Deep Marine Ecosystems (BEEP), 29280, Plouzané, France; 3 University of Toulouse, CNRS, CBI, Toulouse, France; 4 Proteomics Platform, Mass Spectrometry for Biology Unit, Institut Pasteur, Université de Paris-Cité, CNRS USR 2000, 75015, Paris, France; 5 Department of Microbiology, Unit Evolutionary Biology of the Microbial Cell, Institut Pasteur, 75015, Paris, France

**Keywords:** Ribosome, Archaeal biology, Cryoelectron microscopy

## Abstract

Ribosome hibernation preserves translation machinery during stress, yet its mechanisms in Archaea remain poorly defined. Using cryo-EM analysis, we studied hibernation pathways in *Pyrococcus abyssi* stressed cells. We identified HibA, a previously unrecognized family of hibernation factors widespread in Archaea. HibA consists of a bacterial-like HPF/RaiA domain fused to a Cystathionine Beta Synthase module. Unexpectedly, HibA binds to the ribosome in three different conformations, occupying the A, P and E sites of tRNAs, as well as that of mRNA, enhancing its ability to protect the ribosome from degradation. Idle ribosomes also frequently accumulate the archaeal homolog of eukaryotic ribosome maturation protein SBDS (aSBDS), suggesting that stressed archaeal cells may engage parallel hibernation routes in which aSBDS can complement HibA. Deletion of *hibA* in *Thermococcus barophilus* delays recovery from stationary phase and reduces 70S ribosome pools, establishing its role in ribosome preservation. Taxonomic profiling shows that many archaeal lineages encode distinct repertoires of ribosome-associated protection factors, underscoring the modular and multi-layered nature of archaeal hibernation systems. In addition, a comprehensive phylogenetic analysis highlights the evolutionary relationships between prevalent ribosome hibernation factors across Bacteria and Archaea.

## Abstract (web summary)

Ribosome hibernation preserves translation machinery during stress. Here, the authors identify a ribosome hibernation factor that is widespread in archaea and combines bacterial HPF/RaiA-like and cystathionine beta-synthase domains.

## Introduction

Protein synthesis is an essential and highly energy-demanding process that requires tight regulation, particularly under stress or nutrient limitation^1,2^. A major strategy to modulate translation in these conditions is ribosome hibernation, a mechanism that allows cells to reversibly inactivate and protect ribosomes from degradation, thereby conserving resources while preserving the translational machinery for rapid reactivation once favorable conditions return^3–6^. In Bacteria and Eukaryotes, ribosome hibernation is mediated by dedicated protein factors that block the binding of mRNA and tRNAs, halting translation and protecting ribosomes from degradation of their active centers by cellular nucleases^7,8^.

Despite their structural diversity, the loss of these factors contributes to similar phenotypes, i.e., reduced growth resumption, reduced stress tolerance, and accelerated ribosome degradation^9–12^. Bacterial hibernating ribosomes can exist as translationally inactive 70S particles or dimerized 100S complexes, stabilized by protein factors classified in three different families: HPF/RaiA, RMF, and Balon^13–16^. HPF/RaiA proteins (lHPH, HPF, and RaiA) share a structural domain that binds to the 30S small ribosomal subunit (SSU) at the binding sites of mRNA, tRNA, and initiation factors to inactivate the ribosome^17^. In contrast, Balon binds to ribosomes in an mRNA-independent manner^14^. Finally, RMF induces conformational changes in the 30S subunit, promoting the formation of 100S dimers^18^. Eukaryotic ribosome hibernation factors are more diverse and include six families: Stm1/Serbp1, Lso2, IFRD1/IFRD2, MDF1, MDF2, and Dap1b (reviewed in ref. ^14^). These factors were shown to act in a similar way to bacterial factors, by occupying the mRNA channel and either partially or fully blocking tRNA binding sites. However, eukaryotic and bacterial hibernation factors are not homologous, indicating an independent origin^5^.

Ribosome hibernation in Archaea remained largely unexplored until recently, despite its likely importance for survival in extreme and nutrient-limited environments. The archaeal ribosome is of the eukaryotic type. With the exception of the 3’ end of the 16S rRNA, archaeal rRNAs are closer to eukaryotic than to bacterial rRNAs^19–23^. In addition, archaeal ribosomal proteins are either universal or found only in Eukaryotes and Archaea^22–26^. Recently, three archaeal ribosome hibernation factors have been reported. aRDF, restricted to the *Pyrococcus* genus, promotes small-subunit dimerization by binding to the ribosomal protein eS32^27^. Dri, identified in *Pyrobaculum calidifontis*, consists of dual-lobed Cystathionine Beta Synthase (CBS) modules that bind both subunits and block the mRNA channel and the peptidyl transferase center^28^. Dri homologs are unevenly distributed, being largely restricted to *Thermoproteota* or present as single CBS modules, in a subset of other phyla. Finally, in *Methanosarcina acetivorans*, a methanogen ribosome dimerization factor (MRDF) composed of proteins with established functions in purine biosynthesis (PurH) and translation elongation (aEF2) was recently shown to promote the formation of an inactive 50S-dimerization complex. Although PurH and aEF2 are broadly distributed across Archaea, the formation of this repurposed dimerization assembly has so far been observed only in methanogens^29^.

Here, we report the functional and structural characterization of a previously unrecognized family of archaeal ribosome hibernation factors, which we have designated HibA. Combining structural, biochemical, genetic, and phylogenomic approaches, we identify HibA as a factor that inactivates the ribosome and contributes to its preservation during stress. Deletion of *hibA* in the model archaeon *Thermococcus barophilus* delays growth resumption from stationary phase and reduces 70S ribosome pools. High-resolution cryo-EM structures of HibA:ribosome complexes isolated in cell-extract from stressed *Pyrococcus abyssi* reveal that HibA adopts multiple distinct conformations overlapping A, P and E site tRNA positions, indicating that a single factor can have several binding sites on the ribosome. In addition, we observed that HibA frequently co-occurs with an E-site tRNA or with the archaeal version of SBDS (aSBDS, Shwachman-Bodian-Diamond Syndrome), suggesting that stressed cells may mobilize multiple actors to protect inactive ribosomes. Finally, taxonomic analyses depict the diversity of hibernation-related proteins in Archaea. In parallel, our phylogenetic analysis provides insights into the origin and diversification of the HPF/RaiA domain across Bacteria and Archaea.

Together, our findings establish HibA as a key mediator of ribosome hibernation in Archaea. They provide a mechanistic and functional framework to understand how archaeal cells preserve their translational machinery under stress, and open new perspectives on the evolution and regulation of ribosome dormancy across the three domains of life.

## Results

### Identification of HibA as a novel hibernation factor in Archaea

**Fig. 1:**
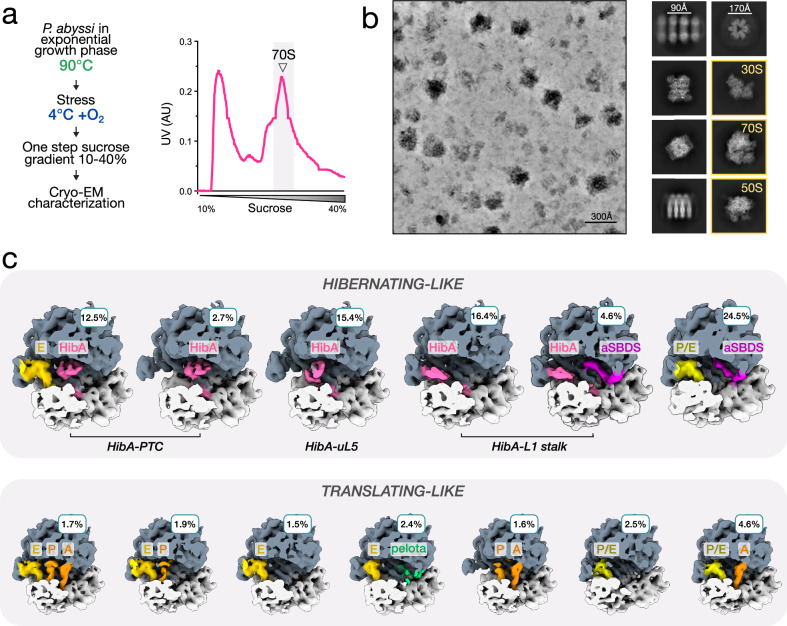
Cryo-EM structures reveal multiple hibernating complexes in cell extracts. **a** Schematic overview of the experimental protocol. *P. abyssi* cells were grown at 90 °C (exponential phase), exposed to cold stress at 4 °C for 1 h, and subjected to oxidative stress during cell harvesting. Ribosomal complexes were isolated by one-step sucrose gradient centrifugation, and the 70S fraction was analyzed by cryo-EM. The corresponding sucrose gradient profile is shown. **b** Representative denoised micrograph among a total of 17,964 (processed with cryoSPARC) illustrating the presence of high-molecular-weight complexes, together with corresponding 2D classes. **c** 3D classes obtained from the 70S particle dataset, with the relative proportion of each class indicated, except the junk/unidentified classes that represent 7.7%. Source data are provided as a Source Data file.

To understand how Thermococcales adapt ribosomal translation to oxidative and cold-shock stresses, we harvested *Pyrococcus abyssi* cells at the end of exponential growth and stored them for 1 h at 4 °C aerobically before centrifugation. We then isolated ribosomes, but instead of using a standard ribosome purification protocol, we only performed one sucrose gradient step to recover the high-molecular-weight fraction, which was directly used for an extensive cryo-EM data collection (Fig. 1a). 70S particles were easily picked from the images. In addition, we also detected many macromolecular complexes, the description of which goes beyond the scope of this article (Fig. 1b and Supplementary Fig. 1). Overall, the large variety of high-molecular-weight complexes observed in the images validates our purification strategy aimed at isolating states close to the native state.

We isolated ~1.5 M 70S particles that were 3D classified, focusing on the tRNA binding sites. 16.2% of the particles were “translating-like”, whereas 76.1% of the particles contained uncharacterized proteins at tRNA binding sites, reflecting “hibernating-like” states (Fig. 1c). Six “hibernating-like” classes were identified. Five classes contained a previously uncharacterized protein (pink in Fig. 1, 75.5% of “hibernating-like” 70S particles) with one class containing an additional protein at the A site side (magenta in Fig. 1c). Finally, the remaining class contained only the A site protein (magenta in Fig. 1c) and P/E tRNA (24.5% of “hibernating-like” particles).

**Fig. 2:**
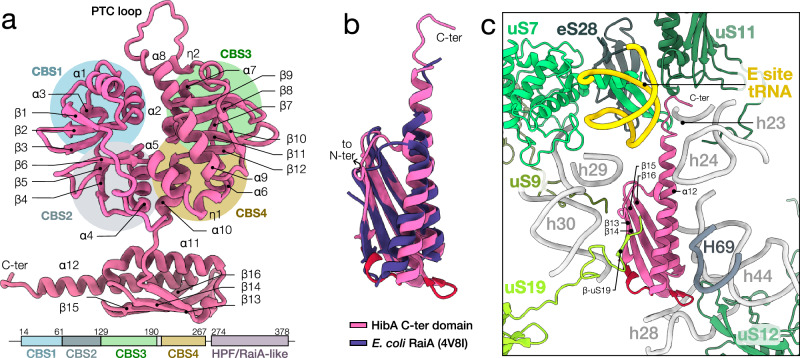
Structure of HibA and the binding site of its C-terminal domain on 30S. **a** HibA 3D structure. The N-terminal domain of HibA (1-269) is made up of four CBS domains. Two CBS domains interact through their beta-sheets to form a Bateman module. Two bateman modules interact to form a disk-like structure defined as a parallel CBS module^84^. Each CBS domain consists of a three-stranded (two parallel and one anti-parallel) β-sheet with two α-helices^85^. One long loop is inserted between α8 and β10 of the third CBS domain. The C-terminal domain (270-387) has the βαβββα topology found in all bacterial HPF/RaiA-like structures. The last five residues are not visible in the cryo-EM map. **b** Comparison of HibA C-terminal domain with *E. coli* RaiA. The two HibA archaeal-specific insertions are colored in red (also in view **c**). **c** HibA C-terminal domain binding site on 30S. HibA contacts h44, h28, h24, h23 helices of the 16S rRNA as well as uS12, uS9, uS19, uS11, uS7 and eS28 ribosomal protein. The C-terminal end of HibA is at the entrance of the mRNA exit channel.

Heterogeneity and conformational variability of each class were addressed using 3D flex refinement in Cryosparc (Supplementary Fig. 1) to resolutions better than 2.5 Å. The 3D structures of the two proteins were initially traced into the cryo-EM maps using ModelAngelo^30^. The A site-binding protein (magenta in Fig. 1) was identified as archaeal SBDS (aSBDS), a three-domain protein ubiquitous in Eukaryotes and Archaea. The second protein (pink in Fig. 1) is made up of two domains. The C-terminal domain is bound similarly on the 30S subunit in all classes, whereas the N-terminal domain is found at different positions on the 50S subunit. Initial building showed that the 30S-bound domain displayed a βαβββα fold similar to that of the bacterial hibernation factor HPF/RaiA. PSI-Blast searches against archaeal genomes revealed a large protein family having low sequence identity with bacterial HPF/RaiA proteins. These proteins were detected in 47% of the archaeal genomes and are widely distributed across all major lineages of Archaea. Members of this family, typically annotated as Cystathionine Beta Synthase (CBS) domain-containing proteins, display a unique modular architecture. The N-terminal part is composed of four CBS domains of about 60 amino acids, arranged in tandem (hereafter named CBS module or CM, residues 1-269), linked to a C-terminal part (residues 270-392) that shares sequence similarities with the core domain of bacterial hibernation promoting factors HPF/RaiA (Fig. 2 and Supplementary Figs. 2, 3). Altogether, our results strongly suggest that this family of proteins is involved in hibernation. Considering its wide distribution, we propose to name this new family HibA for ribosome Hibernation in Archaea (Fig. 2).

Interestingly, aSBDS was identified as a possible partner of HibA in 6% of the “hibernating-like” particles (Fig. 1c). In addition, 32% of the “hibernating-like” particles contained aSBDS, without HibA but with a P/E tRNA (Fig. 1c). Therefore, hibernation in *P. abyssi* could rely on two uncharacterized factors, a previously unidentified protein named HibA and aSBDS. Importantly, mass spectrometry analysis of our 70S fraction (Fig. 1a) showed that HibA and aSBDS are indeed detected in our sample and have iBAQ values similar to those of ribosomal proteins, which supports their high abundance (Supplementary Fig. 4). Given the novelty of HibA, we focused our study on this factor.

### HibA adopts multiple distinct conformations on the ribosome

The C-terminal domain of HibA is positioned in the mRNA:tRNA binding channel in the 30S, in a manner similar to that of bacterial HPF homologs^14,17,18,31^ (Fig. 2b, c and Supplementary Fig. 5a–c). However, in the archaeal case, the number of contacts with the ribosome is strikingly greater. In addition, there are two specific insertions in the archaeal RaiA/HPF domain as compared to the bacterial one (Fig. 2b and Supplementary Fig. 3). Hence, in the A site, HibA contacts the B2a intersubunit bridge (H69:h44), interacts with uS12 as well as with h44-A1461 and G496 decoding residues (ec-h44-A1492, G530 *E. coli* numbering). This contributes to stabilize π-stacking of bases h44-A1462, h44-A1461, and H69-A2154 (ec-H69-A1913 Supplementary Fig. 5a–d). These four bases are universally conserved and known to be crucial for tRNA substrate selection during decoding^32^. Other archaeal specific contacts are observed between the long C-terminal tail of uS19 (Supplementary Fig. 6) and HibA β13. In total, 270 contacts involving 52 HibA residues were identified (Supplementary Data 1).

**Fig. 3:**
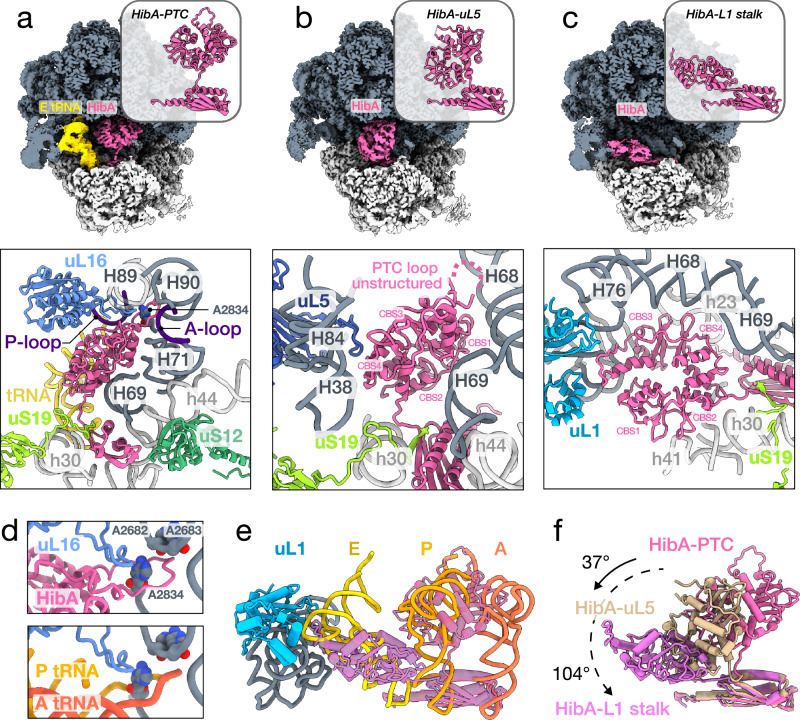
The three positions of HibA N-terminal domain on 50S. **a** HibA-PTC conformation. (Upper part) The cryo-EM map was clipped to show HibA inside the ribosome. HibA is shown in pink, and the E site tRNA in yellow. Ribosomal proteins interacting with HibA are colored and labeled. (Lower part) HibA-PTC environment is represented. **b** HibA-uL5 conformation. (Upper part) Same as (**a**). (Lower part) HibA-uL5 environment is represented. **c** HibA-L1 Stalk conformation. (Upper part) Same as (**a**). (Lower part) HibA-L1 Stalk environment is represented. **d** Comparison of HibA-PTC structure (upper panel) with that of a translating human ribosome (lower panel, PDB 8G61). Only A and P site tRNAs are shown. Base A2834, universally conserved and located between both A- and P-tRNA CCA ends, is sandwiched by the HibA-loop. The uL16 loop is also shown in blue. **e** Superposition of the HibA-PTC and HibA-L1stalk conformations on an archaeal translating ribosome with the three tRNAs (PDB 8HKY). The superposition shows how HibA binding sites overlap those of the tRNAs. **f** Superposition of the three HibA conformations. The rotation angles of the HibA N-domain relative to HibA-PTC are indicated.

Unlike the constant position of the C-terminal domain on the SSU, the N-terminal domain adopted three different positions on the large ribosomal subunit (LSU): bound to the peptidyl-transferase center (PTC) (HibA-PTC; 30% of the HibA-bound particles), between P and E sites (HibA-uL5; 30% of the HibA-bound particles), or at the E site contacting the L1 stalk (HibA-L1 stalk; 40% of the HibA-bound particles) (Figs. 1c and 3). Most of the HibA-PTC particles contained an E site tRNA (Fig. 1c), suggesting that it promotes the stabilization of the HibA-PTC conformation. Notably, comparison of HibA-PTC and HibA-uL5 cryo-EM maps showed that the ribosome is in the same unrotated state. In contrast, HibA-L1 stalk corresponds to a rotated state of the 30S with respect to the 50S.

In the HibA-PTC conformation (Fig. 3a and Supplementary Fig. 7a), a long loop (residues 190-207, hereafter named PTC loop) is positioned like the CCA ends of A and P tRNAs and surrounds A2834, a universally conserved adenine of the 23S rRNA, sandwiched between the A and P sites on the LSU and known to play a key role in peptide bond formation (Fig. 3d). The HibA PTC loop also engages a large number of interactions with universally conserved nucleotides^33–35^ of the A and P sites at the PTC. In addition, a long loop of uL16 overhangs HibA PTC loop and contributes to the stabilization of 23S-A2834. On the other hand, CBS domains interact with the upper part of 23S-H69, while the distal part interacts with the HibA C-terminal domain. Overall, the HibA N-terminal domain binds PTC residues critical for tRNAs binding and catalysis, and protects them from degradation.

In the HibA-uL5 conformation (Fig. 3b and Supplementary Fig. 7b), the N-terminal domain rotates by 37° relative to HibA-PTC (Fig. 3f). CBS3 and 4 overlap the elbow and the acceptor helix of the P site tRNA whereas CBS1 and 2 overlap the D and anticodon stems of the E site tRNA (Supplementary Fig. 8b). CBS4 contacts uL5 that makes part of the B1a-B1b/c bridge connecting the 50S to the 30S subunit. Only a few contacts are observed, suggesting that the HibA N-domain can move easily (Supplementary Fig. 7b and Supplementary Data 1).

For the third conformation (HibA-L1 stalk, Fig. 3c, e, f, Supplementary Fig. 7c and Supplementary Data 1), to improve the quality of the cryo-EM map in the region of the HibA binding site, we performed a local refinement following particle subtraction. The N-domain of HibA rotates by 104° (relative to HibA-PTC) and binds to the L1 stalk through contacts with uL1 and the 23S rRNA. The position of the domain corresponds to that of the anticodon helix of an E site tRNA (Fig. 3e and Supplementary Fig. 8c). The SSU is rotated as in a post-translocation state.

In summary, HibA acts as a hibernation factor by stabilizing an inactive 70S conformation and occluding key functional sites (Supplementary Fig. 8), thereby protecting the ribosome from dissociation and degradation (Fig. 3 and Supplementary Fig. 8). The fusion between the two structural domains of HibA, which interact with the SSU and the LSU, clearly contributes to maintaining the fully assembled ribosome. In addition, the function of HibA benefits from conformational plasticity on the ribosome that is unique among known hibernation factors.

### Multi-layered ribosome protection and inactivation

**Fig. 4:**
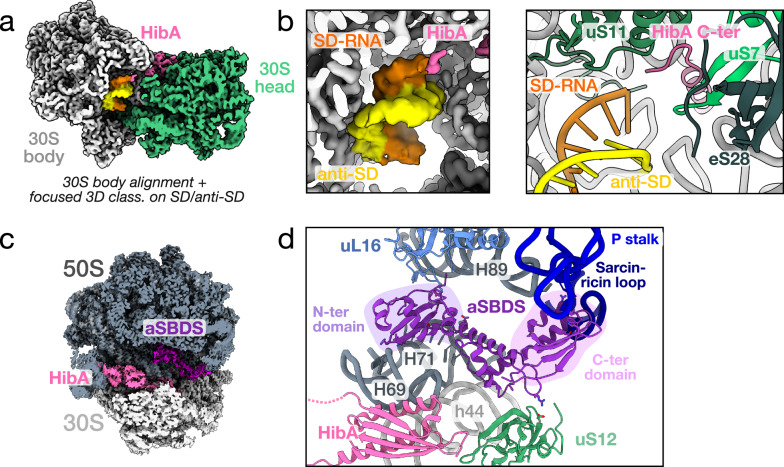
Involvement of aSBDS and SD:antiSD duplex in ribosome hibernation. **a** 2.1 Å cryo-EM map from focused classification on SD:antiSD. The SD:antiSD duplex is in orange and yellow. The C-terminal end of HibA is observed in proximity to the SD:antiSD duplex. **b** Close-up on the SD:anti-SD duplex. Color code is the same as in panel **a**. **c** The cryo-EM map was clipped to show HibA and aSBDS inside the ribosome. HibA is in pink, aSBDS is in magenta. **d** The N-terminal domain of aSBDS interacts with uL16 in the PTC, the intermediate domain contacts uS12, and the C-domain interacts with the P stalk and the Sarcin-ricin region. Contacts are detailed in Supplementary Data 1.

During model building, we observed extra density near the anti-SD sequence at the 3’ end of the 16S rRNA. We did focused-classification on the mRNA exit channel (Supplementary Fig. 1), which revealed the presence of an SD:antiSD helix with 9 paired bases in 17% of the HibA-containing particles (Fig. 4a,b). Interestingly, the C-terminal end of HibA is observed in proximity of the SD:antiSD duplex (Fig. 3a). This suggests that HibA-mediated ribosome inactivation may involve the binding of an SD sequence forming an SD:antiSD duplex, possibly from a short regulatory RNA or an mRNA degradation product. This SD:antiSD helix may protect the 16S rRNA from its 3’ degradation as reported in bacteria^36^.

As mentioned above, aSBDS was identified together with HibA-L1 stalk in 6% of the “hibernating-like” particles (Fig. 4c, d and Supplementary Fig. 9). The N-terminal domain of aSBDS occupies the PTC, the second domain interacts with uS12, and the C-terminal domain contacts the P stalk and the Sarcin-Ricin loop (Fig. 4c, d). In eukaryotes, SBDS is an essential and conserved factor that controls the final stages of the 60S ribosomal subunit biogenesis, acting in concert with eEFL1 to remove eIF6 from the nascent 60S^37,38^. However, an involvement of aSBDS in aIF6 release has not been observed in Archaea^39^. Here, we found aSBDS bound to the ribosome together with HibA or associated with an E-site tRNA. Its position protects not only the PTC but also the P stalk and the Sarcin-Ricin loop that remain vacant in the absence of aa-tRNA:EF1A. This suggests that SBDS may contribute to ribosome preservation under stress conditions. Interestingly, the conformation observed here corresponds to that of SBDS bound to 60S in Eukaryotes^38^, raising the possibility that SBDS could also interact with the full ribosome in Eukaryotes. The position of the C-terminal domain of aSBDS overlaps that occupied by the tRNA elbow as observed during the first step of accommodation when an aminoacyl-tRNA:EF1A complex is bound to the ribosome (Supplementary Fig. 9a, c). Consequently, when HibA binds in the “L1 stalk” conformation, the vacant functional sites of the ribosome (PTC, GTPase activating center) can be occupied by aSBDS, which thus complements the action of HibA in blocking the ribosome in an inactive state that even prevents aa-tRNA:aEF1A binding.

### In vitro reconstitution of HibA:ribosome complexes

To further characterize the mechanism of action of HibA, we next produced and purified a His-tagged recombinant version of HibA from *Pyrococcus abyssi* (Pa-HibA, uniprot: Q9UYR4) and used it to reconstitute HibA:ribosome complexes assembled from purified 30S and 50S subunits (Supplementary Fig. 10a–c). We collected a large data set of 70S:HibA assemblies (~30k movies) on a Titan Krios microscope (Supplementary Fig. 10d) and obtained ~840k 70S particles. 3D classification identified the same three distinct 70S:HibA conformations, HibA-PTC (8.7%), HibA-uL5 (40%), and HibA-L1 stalk (51.4%), with the C-terminal domain consistently occupying the mRNA:tRNA channel of the 30S subunit. This shows that these three states are intrinsic to HibA and do not require additional cellular factors. Notably, the HibA-PTC state is less represented, suggesting that in vitro conditions are less favorable for this conformation, possibly because an E-site tRNA is missing.

In addition to 70S ribosomes, we identified 50S bound to aIF6, with aIF6 preventing 50S association to 30S. No 50S bound to HibA were observed, suggesting that 50S:HibA complexes have low stability. In contrast, we identified HibA-bound 30S particles (Supplementary Fig. 10d) where the C-terminal domain occupied the mRNA binding channel. HibA N-terminal domain remained unresolved, consistent with its increased mobility in the absence of the LSU.

**Fig. 5:**
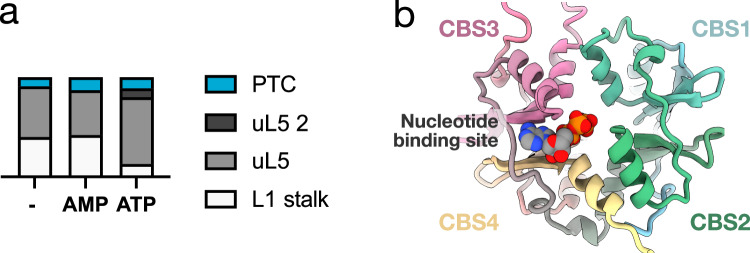
AMP/ATP binding to HibA in 70S hibernating complexes. **a** Proportions of HibA states in reconstituted complexes in the absence of added nucleotide or in the presence of either AMP or ATP. **b** Nucleotide binding site on the CBS module. ATP is represented but AMP binds at the same site (see also Supplementary Figs. 12 and 13).

Surprisingly, despite the known propensity of the CBS modules to bind nucleotides, we did not observe any nucleotide bound to HibA-CBS module (HibA-CM) in any conformational state of the native HibA:ribosome complexes (Fig. 1). Thus, we first tested nucleotide-binding by the isolated HibA-CM using ITC (Supplementary Fig. 11). HibA-CM bound adenylated nucleotides with highest affinity for AMP (Kd = 12 ± 2 µM), then ADP (Kd = 65 ± 1 µM) and ATP (Kd = 113 ± 21 µM). The stoichiometry was consistent with one nucleotide per HibA-CM (Supplementary Fig. 11). In contrast, no heat signal was obtained when GTP or GMP was used in ITC titrations. Then, we used our reconstitution strategy to examine whether adenylated nucleotides could modulate HibA positioning or the distribution of its three conformations in reconstituted 70S:HibA assemblies. Ribosomal complexes were assembled in presence of 5 mM AMP-Mg^2+^ or ATP-Mg^2+^ and processed as above. AMP binding did not change classes conformation and distribution (Fig. 5a and Supplementary Fig. 12). Although AMP was clearly visible in the cryo-EM map, we did not refine the model because of the limited resolution (Supplementary Fig. 12). In the presence of ATP, the three canonical conformations of HibA were observed, but an additional, weakly populated uL5 state was detected (uL5-2 in Fig. 5a and Supplementary Fig. 13). ATP binding was observed in PTC and uL5 states at the interface between CBS3 and 4 (Fig. 5b), as AMP (Supplementary Fig. 12). Superposition of the free and ATP-bound states showed a slight adjustment of the position of CBS3, indicating limited local adjustments rather than major rearrangements. The additional uL5 state could reflect increased ribosome:HibA conformational dynamics in the presence of ATP. However, since these experiments were performed at equilibrium with saturating amounts of ATP and in the absence of mRNA and tRNA competition, it is difficult to conclude on a potential direct role of ATP in the release of HibA from the ribosome. Thus, the overall conformational landscape of HibA on the ribosome remained largely unchanged in the presence of either AMP or ATP.

### HibA inhibits translation, preserves 70S ribosomes, and promotes recovery from the stationary phase

**Fig. 6:**
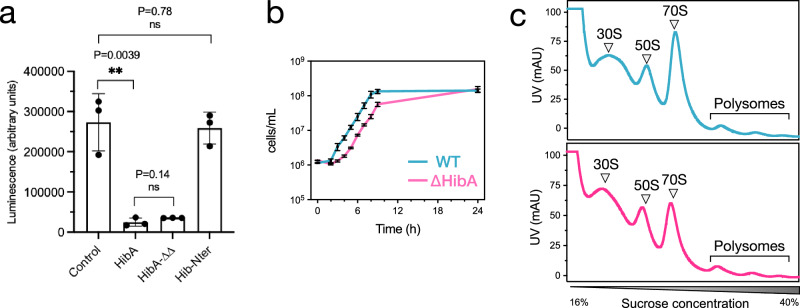
Translation inhibition and phenotypic analyses. **a** HibA inhibits translation in *P. abyssi* cell extracts. In vitro translation using the Nanoluc system (see Materials and methods) was performed in the absence (control) or presence of 6 µM HibA or of the indicated variants. Experiments were performed in triplicate. The results are reported in the graph with the means and standard deviations. *p* values were derived from two-tailed *t*-tests using Prism (GraphPad). Deletions in the HibA-loops (HibA-∆∆) do not significantly modify inhibition as compared to HibA. The isolated N-terminal domain does not significantly inhibit translation. **b** WT (blue) and ∆*hibA* (pink) cells grown in TRM medium at 85 °C for 24 h were used for inoculating fresh TRM medium. Cells were then incubated at 85 °C, and their growth was monitored by flow cytometry at various times after inoculation. Data are presented as mean values ± SEM (*n* = 3 independent biological replicates per strain). **c** 3 mg of total proteins, from WT (blue) and ∆*hibA* (pink) cells in stationary phase (15 h), were fractionated on 10 to 50% sucrose density gradients by ultracentrifugation. The free 30S and 50S ribosomal subunits, 70S ribosomes, and polysome fractions are indicated. Source data are provided as a Source Data file.

To evidence the ability of HibA to compete with tRNA and mRNA bindings and inhibit translation, we set up an in vitro assay based on translation of a HiBiT peptide reporter using a *P. abyssi* cell extract. We optimized the conditions as described in the “Methods” section. Upon addition of an mRNA encoding the HiBiT peptide, we observed efficient translation at 80 °C. In the presence of 6 µM HibA protein, translation was strongly inhibited (Fig. 6a). In addition, when the isolated N-terminal domain of HibA was used in the assay, no significant inhibition was observed. This shows that the C-terminal domain is essential for the efficient activity of HibA. We also attempted to produce the isolated C-terminal domain in both *P. abyssi* and *T. barophilus* species, but despite several efforts, we could not obtain it in a soluble form.

As the ribosome hibernation factors have been shown to enhance cellular stress survival, we generated a *hibA*-deleted mutant strain of *Thermococcus barophilus* (uniprot: F0LKU5), a genetically tractable *Thermococcales* species from the same ecological niche as *P. abyssi*^40^. As shown in Fig. 6b, regrowth of stationary-phase Δ*hibA* mutant cells was significantly delayed compared with that of wild-type cells: lag phases for WΤ and Δ*hibA* lasted 2 and 4 h, respectively, whereas the growth rate in exponential phases remained similar for both strains. To test whether this phenotype reflected reduced viability of the Δ*hibA* mutant, we performed Most Probable Number assays after culturing the cells in nutriment-deprived medium for up to 120 h. Δ*hibA* displayed a slightly greater loss of viability than WT during the first 20 h, followed by a modestly lower viability over 20–120 h (Supplementary Fig. 14), but the magnitude of this effect remained limited (Supplementary Fig. 14). Instead, sucrose gradient analyses revealed that the number of 70S ribosomes in Δ*hibA* stationary-phase cells was ~60% lower than in WT, as determined by peak area comparisons (Fig. 6c). This substantial decrease in 70S particles demonstrates that HibA is crucial for maintaining the stability and integrity of the inactive ribosome pool during stationary phase, a condition in which cells experience combined nutrient and physicochemical stresses. Accordingly, the impaired growth recovery of the Δ*hibA* strain likely reflects both the reduced abundance of intact 70S particles and the time required to rebuild a functional ribosome pool before translation and growth can restart.

### Wide distribution of HibA, complementing potential alternative hibernation factors in Archaea

**Fig. 7:**
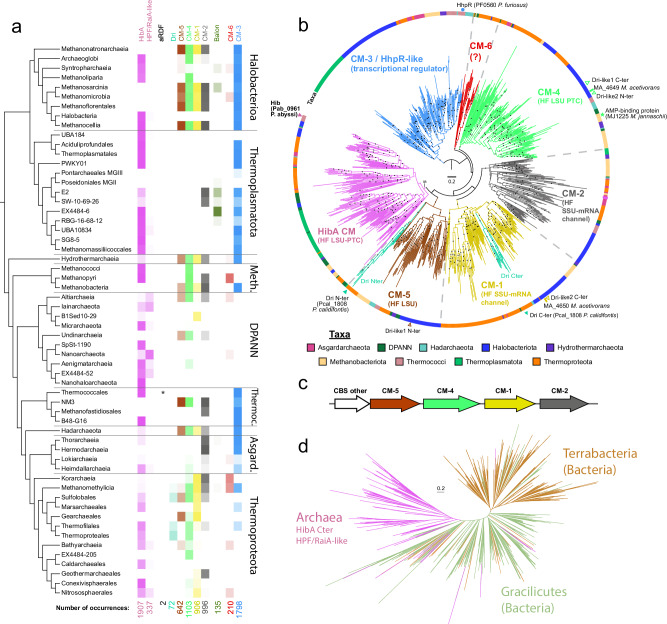
Distribution and phylogenetic relationships between hibernation factors in Archaea. **a** Distribution of HibA, HPF/RaiA-like, aRDF, Dri, CBS module (CM) families (CM-1 to CM-6) and potential Balon in Archaea (database of 4026 archaea). The total number of archaea in which these proteins occur is indicated below the heatmap. * Indicates the presence of aRDF which otherwise would not be visible with heatmap shading. Meth., *Methanobacteriota*, Thermoc., *Thermococcales*, Asgard., *Asgardarchaeota*. **b** Phylogeny of the CM. Branches are colored according to CM-containing proteins in (**a**). The outer layer indicates the taxonomic origin of the sequences. Position of experimentally characterized proteins is indicated by a filled symbol. HF hibernation factor. SSU-mRNA and LSU-PTC indicate potential interaction sites of the CM members with the ribosome. The tree was rooted on CM-3, which was separated from the other CM by the longest branch and may have a different type of interaction (transcription regulator) than the others. Maximum-likelihood tree (LG + R7) based on a trimmed alignment of 230 amino acid positions. Black dots on the branches indicate ultrafast bootstrap supports >90%. The support value of the deepest branch of the HibA CM family is indicated on the tree. **c** Example of CM genes clustering occurring in 10% of the archaeal genomes. **d** Phylogeny of the HPF/RaiA domain from archaea and bacteria. Source data are provided as a Source Data file.

HibA was detected in all major lineages of Archaea, corresponding to 47% of all genomes (Fig. 7a and Supplementary Fig. 15). In addition to canonical HibA, we identified proteins composed solely of the HPF/RaiA-like domain. We hereafter refer to these proteins as HPF/RaiA-like, present in 8% of the archaea, mostly DPANN and *Nitrososphaerales* (Fig. 7a and Supplementary Fig. 15). By analogy with Bacteria, these proteins may also contribute to ribosome hibernation.

The absence of HibA from about half of the Archaea, together with the narrow distribution of the recently described Dri and aRDF factors^27,28^ (Fig. 7a), suggested that additional hibernation factors remained to be identified. Furthermore, a complex of repurposed PurH and aEF2 has been observed to promote 50S dimerization in *Methanosarcina acetivorans*, representing an additional mode of ribosome inactivation^29^. Despite the widespread occurrence of PurH and aEF2 in Archaea, whether this innovation emerged elsewhere remains unclear. In contrast, both HibA and Dri contain CBS modules, and standalone CM proteins were also identified in dormant ribosomes of *Methanosarcina acetivorans* by mass spectrometry^28^. We therefore explored the diversity of CMs as potential hibernation factors. A phylogenetic analysis delineated six CM families (CM-1 to CM-6) with strong support (Fig. 7b), often arranged in gene clusters (Fig. 7c). Domain placement within this phylogeny indicates that Dri originated from a fusion of CM-1 and CM-5 in *Thermoproteota* (Fig. 7b). Interestingly, CM-5 and HibA-CM form a well-supported clade, in agreement with the interactions of both Dri N-terminal and HibA-CM with the LSU. Proteins identified in dormant ribosomes of *M. acetivorans* correspond to CM-1 and CM-4. The phyletic distribution of these CM families further provides clues about their involvement in ribosome hibernation. CM-1, CM-2, CM-4 and CM-5 are widespread but rarely co-occur with HibA (Fig. 7a and Supplementary Figs. 15, 16). CM-2 and CM-5 in particular display the strongest patterns of exclusion with HibA. Such an exclusion pattern suggests that Archaea generally retain either HibA or CM-1/2/4/5 (fused or standalone) as hibernation factors. In contrast, CM-3 does not display a complementary phyletic pattern with HibA, suggesting that it may not play a role in ribosome hibernation. This is in agreement with the *Pyrococcus yayanosii* HhpR transcription factor, belonging to the CM-3 family^41^. CM-6 have a narrow distribution, mostly in *Thermoproteota*, and cluster with eukaryotic AMPKγ (Supplementary Fig. 17), also suggesting that they are unlikely to be involved in ribosome hibernation.

Finally, we searched for additional homologs of bacterial hibernation factors lacking CBS and/or HPF/RaiA domains. This led to the identification of homologs of the recently described bacterial hibernation factor Balon^14^ in some *Halobacteriota* (i.e., 30% of the *Methanosarcinia* and 10% of the *Syntropharchaeia*) and several *Thermoplasmatota* lineages (up to 84% in EX4484-6) (Fig. 7a). Their restricted distribution suggests a lineage-specific adaptation. Overall, 84% of the Archaea have HibA, HibA-Cter, Dri, standalone CM-1/2/4/5, and/or the potential Balon. Among the few lineages lacking identifiable factors, marine planktonic groups are the most notable (*Poseidoniia* (*Thermoplasmatota*) and *Nitrosopumilaceae* (*Thermoprotei*), Fig. 7a), suggesting either a reduced requirement for ribosome hibernation factors in the ocean water column or the existence of yet unrecognized hibernation factors.

### Evolution of hibernation factors comprising the HPF/RaiA and CBS modules

**Fig. 8:**
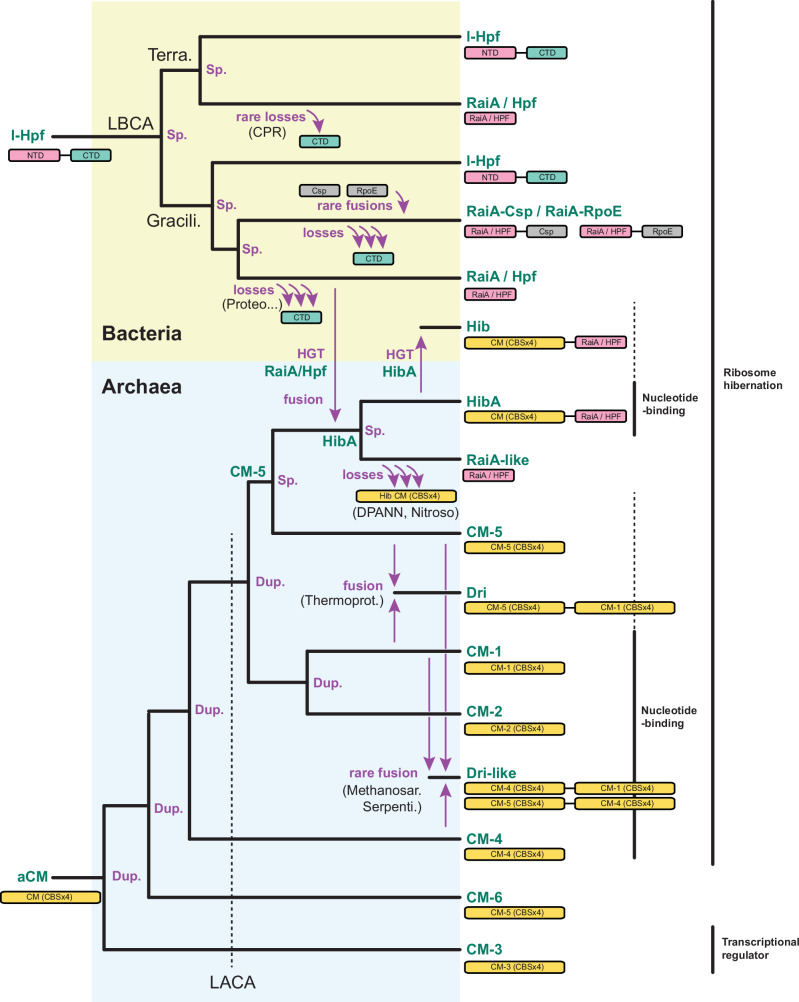
Evolutionary scenario of proteins containing the RaiA/HPF domain and CBS modules (CM) in Archaea and Bacteria. Sp. speciation, Dup. duplication, Proteo. Proteobacteria, Nitroso. Nitrososphaerales, Thermoprot. Thermoproteota, Methanosar. Methanosarcinaceae, Serpenti. Serpentinarchaeaceae. See text for description.

The wide distribution of HibA in Archaea reveals that the HPF/RaiA domain contributes to ribosome hibernation beyond Bacteria (Supplementary Fig. 18). Current knowledge of its distribution in Bacteria remains very limited^16^. The discovery of a widely distributed ribosome hibernation function in Archaea now provides the first opportunity to investigate evolutionary relationships among HPF/RaiA proteins across bacteria and archaea. To this aim, we reconstructed phylogenies of HPF/RaiA-domain proteins in both Archaea and Bacteria and analyzed patterns of domain association (Fig. 8).

In Bacteria, the phylogeny of the HPF/RaiA domain (lHPF-NTD, HPF, and RaiA proteins, see Supplementary Fig. 3a) reveals a split between the two earliest bacterial branches, *Terrabacteria* and *Gracilicutes*, yet a large distribution across both lineages (Supplementary Fig. 19), consistent with its presence in the Last Bacterial Common Ancestor (LBCA). Mapping of lHPF, HPF/RaiA onto this phylogeny shows that most *Terrabacteria* have lHPF, whereas *Gracilicutes* have both lHPF and HPF/RaiA. Because the CTD of lHPF does not occur as a standalone protein, the current diversity of lHPF is unlikely to reflect multiple independent fusion events. Instead, our data support lHPF as the ancestral bacterial hibernation factor, with the current diversity of HPF/RaiA resulting from multiple losses of lHPF-CTD during bacterial evolution (Fig. 8).

In Archaea, HibA-CM forms a monophyletic group, closely related to CM-5 (Fig. 7b), consistent with a fusion between an ancestral CM-5 and an HPF/RaiA-like protein. This raises the question of the origin of the “unfused” HPF/RaiA proteins present in current Archaea. HPF/RaiA-like proteins are less common than HibA in Archaea and are generally nested within large HibA clades (Supplementary Fig. 20), supporting multiple emergences through HibA-CM losses. The hypothesis of a single fusion between CM-5 and HPF/RaiA-like protein further implies that ribosome binding by standalone CM predates the emergence of HibA. Indeed, CM-5 arose by duplication events that also led to CM-1 and CM-4 (Figs. 7b and 8), that have been detected in dormant ribosomes^28^. Thus, their ancestor may already have played a role in ribosome hibernation.

In the phylogeny of CM-3, all major lineages are represented and monophyletic, suggesting that the duplication separating CM-3 transcriptional regulator from families of hibernation factors occurred before the Last Archaeal Common Ancestor (LACA). This would indicate that ribosome hibernation based on CM proteins lacking the HPF/RaiA domain was present in the LACA.

Finally, we built a combined phylogeny of archaeal (HibA Cter + HPF/RaiA proteins) and bacterial (lHPF NTD + HPF + RaiA) HPF/RaiA domains to infer their phylogenetic relationships. The position of Archaea suggests acquisition of their HPF/RaiA through an early horizontal gene transfer (HGT) from Bacteria (Figs. 7d and 8). This interpretation should, however, be taken with caution because of the small size (<100 aa after trimming) and fast evolution of the domain. HGT nevertheless remains our preferred hypothesis, as the alternative scenario, where HPF/RaiA-like proteins and standalone CM coexisted in Archaea before fusion with CM-5, is not supported by the current mutual exclusion between HibA, HPF/RaiA-like, and CM-5/1/2/4.

## Discussion

Ribosome hibernation factors are central to cellular adaptation under stress. In Archaea, their distribution and physiological roles remain largely unresolved, despite the recent identification of Dri, aRDF, and MRDF^27–29^. The discovery and characterization of HibA provide functional insight into the contribution of archaeal ribosome hibernation to stress adaptation. Genetic analysis demonstrates that HibA is critical for efficient recovery from stress in Archaea. In *T. barophilus*, deletion of *hibA* led to a pronounced delay in regrowth from the stationary phase. In accordance, bacterial ribosome hibernation factors have been consistently shown to be implicated in the ability of cells to resume growth after stress^6^. Mutants lacking HPF or RMF hibernation factors display extended lag phases during recovery from the stationary phase^11,12^ or after nutrient starvation^9,42^. Beyond these observations, mechanistic studies suggest that the growth-recovery defect of such mutants can be attributed to a reduction in 70S/100S ribosomes under stress, followed by nucleolytic degradation of ribosomal subunits by the exoribonuclease RNase R^7,8^. This framework is consistent with our finding that the *ΔhibA* mutant of *T. barophilus* exhibited a marked reduction of 70S ribosomes during the stationary phase. By contrast, canonical RNase R homologs are mostly absent from Archaea, and 3’ end ribonucleolytic turnover is primarily mediated by RNA exosome^43^. Together, these data support a conserved role of ribosome hibernation in preserving translation-competent ribosomes under stress. The archaeal HibA factor contributes to ribosome preservation in a manner comparable to bacterial hibernation factors. However, the molecular mechanisms leading to ribosomal degradation might differ between domains and remain to be characterized. The viability assays revealed no detectable difference over a long period of stress between the wild type and the *ΔhibA* strains. In bacteria, the impact of ribosome hibernation on stress period survival is variable. While some studies report reduced viability of hibernation mutants, others find little to no effect, highlighting that the behavior depends strongly on the stress conditions and genetic background tested (reviewed in ref. ^6^). In this instance, it is possible that the conditions assayed may not have been stringent enough to reveal a viability defect, or alternatively that Archaea may possess redundant hibernation factors able to partially compensate for the loss of HibA under prolonged stress. In support of this latter hypothesis, we detected aSBDS, with or without a tRNA bound in the ribosomal site, in a substantial fraction of dormant ribosomes, suggesting the coexistence of alternative ribosome-associated mechanisms contributing to translational silencing or stabilization under stress. Consistent with its protective role, the expression of *hibA* in Thermococcales is controlled by the transcriptional regulator Phr, a master repressor of the archaeal heat shock response^44^. Taken together, its physiological relevance and regulation by Phr place *hibA* among archaeal stress-responsive genes, contributing to adaptation across multiple stress conditions.

HibA displays a unique modular organization combining a bacterial-type HPF/RaiA core with a CBS module. The C-terminal domain of HibA makes extensive contacts with rRNA and r-proteins in the mRNA channel of the SSU. Comparison with bacterial ribosome structures bound to HPF/RaiA homologs shows that most of the contact points of the hibernation factors with ribosomal RNA are conserved. However, in bacteria, interactions with ribosomal proteins are more limited, and only interactions with uS9 and uS7 have been identified in some cases^14,17,18,31^ (Supplementary Figs. 3,5). Structural and sequence alignments of the HibA C-terminal domain with bacterial HPF/RaiA proteins show two archaeal-specific insertions absent from most bacterial homologs (Supplementary Fig. 3). One insertion, located between β13 and α11, contributes to the binding site for the C-terminal tail of uS19 that has an extension of 8-9 residues in Archaea and Eukaryotes compared with its bacterial counterpart (Supplementary Fig. 6). The second insertion, located between β14 and β15, is bound to uS12 and participates in the stabilization of the universally conserved bases crucial for tRNA substrate selection during decoding in the A site^32^ (Supplementary Fig. 5). However, deletion of the two archaeal-specific insertions (HibA-∆∆, Methods) did not change the translation inhibition activity of HibA in our in vitro assay (Fig. 6a), but this is likely due to the large number of other contacts involved in HibA binding.

HibA binds to the LSU through its N-terminal CBS module. Orientation of the CBS module defines the three conformations of HibA observed here, in the PTC, bound to uL5 in the B1a-B1b/c bridge or bound to the L1 stalk. In the HibA-PTC conformation, the long HibA PTC loop interacts with nucleotide residues crucial for the peptidyl-transferase activity and with uL16. Although uL16 is universally positioned close to the A-site tRNA binding site, only eukaryotic and archaeal uL16 have an extended loop inserted into the PTC (Supplementary Fig. 21). The uL16 loop is likely important during catalysis, potentially contributing to the positioning of the tRNA CCA-ends and/or substrate accommodation at the PTC^45,46^. Thus, HibA not only binds important nucleotides but also targets important ribosomal protein regions that are characteristic of archaeal ribosomes.

In all three conformations of HibA, the CBS module occupies vacant binding sites normally occupied by tRNAs during translation. Therefore, HibA provides a reversible ribosome inactivation mechanism, in competition with tRNAs and mRNA bindings when favorable growth conditions return. To our knowledge, multiple conformations have not been described before for a ribosomal hibernation factor. Thus, the possibility of conformational variability should be considered in future structural studies of hibernation proteins. These three conformations collectively protect the ribosome active sites from degradation, which could improve the efficiency of the hibernation mechanism. Alternatively, the three conformations of HibA may also illustrate routes used by the factor to bind and detach from the ribosome. Finally, we observed that conserved residues in the N-terminal domains of HibA orthologs are mainly located at the core of the CBS module rather than in the regions in contact with the ribosome (Supplementary Fig. 22). This supports a mode of action for the CBS modules of HibA based on the occupation of vacant sites rather than on specific interactions. The anchoring of HibA to the ribosome via the strong binding of its C-terminal domain to the 30S would facilitate the dynamic exploration of vacant sites on the 50S and their protection.

The importance of CBS modules in hibernation has recently been underscored by the identification of Dri, an archaeal ribosome hibernation factor containing two CBS modules^28^. One CBS module of Dri is observed bound to the PTC. However, the binding of HibA and Dri to PTC differs markedly, with their CBS modules adopting opposite orientations (Supplementary Fig. 23). In Dri and HibA, a long loop is inserted between CBS3 and CBS4. However, the loop adopts different conformations and binding sites on the ribosome (Supplementary Fig. 22). This shows that the versatility of the CBS module is important for the evolution of these hibernation factors. Notably, a long loop located in the same place, i.e., between CBS3 and 4, is also present and important for the function in the C-terminal CBS module of Dri and in the recently described stand-alone CBS module protein HhpR, acting as a pressure-dependent transcriptional regulator in *P. yayanosii*^41^. Therefore, such insertions could play a recurrent role in the function of these CBS modules.

CBS modules, either standalone or fused with functional domains, are widespread and function as regulatory modules that connect cellular or environmental signals with essential processes. They bind a variety of small ligands, most commonly adenylates but also S-adenosyl methionine, diadenosine polyphosphates, or divalent cations, and depending on the ligand, may activate or inhibit their protein partner^47^. Here, HibA CBS module preferentially binds AMP, consistent with the general principle that AMP is a sensitive indicator of cellular energy stress^48^. We observed no clear effect of ATP or AMP on HibA binding to the ribosome. Nevertheless, nucleotide binding could still contribute to HibA engagement or release, or alternatively regulate its interaction with a sequestering partner. In support of this hypothesis, we and others have detected by pulldown approaches coupled to mass spectrometry analyses, interactions between HibA and key actors of DNA recombination and initiation of DNA replication in *Thermococcales* (HibA was referred to as Pab0961 and TK1186 in these studies)^49,50^. In this case, the cytoplasmic concentration of HibA available for its binding to the ribosome would be regulated depending on the ATP/AMP ratio. This possibility remains to be explored.

Together, these observations suggest that the CBS module provides an additional regulatory layer to ribosome hibernation. Yet our structural data also indicate that HibA may act within a broader network of structural or regulatory partners. In the HibA-PTC conformation, the presence of deacylated tRNA in the E site is consistent with the accumulation of uncharged tRNA observed under stress conditions^51^. The E site tRNA may act as a signal to facilitate the recruitment of HibA and to favor the HibA-PTC conformation. In addition to tRNA, aSBDS was observed in a subset of HibA-L1 stalk particles (Fig. 2c), suggesting a function for aSBDS not anticipated from studies of its eukaryotic homolog. Finally, our work suggests that ribosome hibernation in Archaea may also involve the formation of an SD:antiSD duplex that would protect the 3’ end of the 16S rRNA, highly sensitive to RNase-mediated degradation^36^. Thus, ribosome preservation in Thermococcales would be achieved through the protection of all active sites of the ribosome, from the mRNA entry channel to the mRNA exit channel. Altogether, these additional factors highlight that archaeal ribosome hibernation is a multi-layered process, potentially integrating both protein and RNA-based mechanisms.

Our phylogenetic analyses reveal the evolutionary relationships of HPF/RaiA family proteins in Bacteria and Archaea and establish lHPF as the ancestral bacterial hibernation factor. In Archaea, by contrast, the topology and distribution patterns point to an ancestral CM mediated hibernation mechanism predating HibA, with subsequent domain fusions generating archaeal solutions: the association of an HPF/RaiA-like protein with CM-5 to form HibA, the CM-1/CM-5 fusion that produced Dri in *Thermoproteota*, and the CM-1/CM-4 in *M. activorans*. Together, these observations highlight the modularity of archaeal hibernation systems, in which CBS modules and HPF/RaiA-like proteins function either as standalone components or in fusion, and illustrate how CBS modules diversified in Archaea to support ribosome hibernation (HibA, Dri) as well as transcriptional regulation (HhpR). More broadly, archaeal CBS proteins may constitute a primordial regulatory toolkit linking cellular energy and environmental signals to essential cellular processes.

These evolutionary insights open perspectives for identifying additional hibernation factors in Archaea. While CM-1, CM-4, and CM-5 have already been detected in dormant ribosomes^28^, the role of CM-2 and CM-6 remains unresolved. Phylogeny and exclusion patterns suggest that CM-2 may also bind the ribosome, whereas the distribution of CM-6 points to a distinct function yet to be explored. In parallel, the detection of potential Balon homologs in Archaea, and their established role as a hibernation factor in Bacteria^14^, support the possibility that Balon-like proteins also contribute to archaeal ribosome hibernation.

Ecological patterns may also highlight niche-dependent specialization of hibernation strategies. Canonical factors are absent from dominant marine planktonic groups, suggesting that the relatively stable and nutrient-balanced conditions of the ocean water column reduce the selective pressure for such mechanisms. A comparable trend is observed among methanogens: CM-1 and CM-5 (corresponding to the N- and C-terminal moieties of Dri) are specifically absent from gut-associated species (*Methanobrevibacter*, *Methanosphaera*, *Methanocorpusculum*, *Methanomicrobium*) but retained in their closest environmental relative species carrying the CM-5/4/1/2 cluster. This contrast indicates that hibernation strategies involving CM-1 and CM-5 are more important in fluctuating, nutrient-poor environments than in the stable, nutrient-rich conditions of the gut. Accounting for microbial diversity and ecology will be essential for future investigations of archaeal hibernation strategies.

In conclusion, this study closes a major gap by establishing ribosome hibernation as an adaptive strategy to stress in Archaea, thereby extending this mechanism to all three domains of life. HibA emerges as a key factor in this process, linking archaeal hibernation to the conserved HPF/RaiA domain central to prokaryotic strategies. We also highlight that archaeal ribosome hibernation has been shaped by CBS modularity and ribosomal plasticity during evolution. Furthermore, this work provides a solid foundation for future work aimed at investigating the role of the potential partners identified, the integration of ribosome hibernation into global stress and energy networks, and the candidate additional ribosome factors in Archaea, yet to be experimentally tested.

## Methods

### Strains, media, and growth conditions

Bacterial and archaeal strains are listed in Supplementary Table 1. *E. coli* strain DH5α was used as the general cloning host. This bacterium was cultivated in Luria-Bertani (LB) broth. Growth of *T. barophilus* was performed in Thermococcales-rich medium (TRM), under anaerobic conditions at 85 °C as previously described^52^. When necessary, media were supplemented with ampicillin (25 μg/mL) for *E. coli*, simvastatin (2.5 μg/mL), and 6MP (100 µM) for *T. barophilus*. Depending on the experiment, elemental or colloidal sulfur (0.1% or 0.5 g/L final concentration) was added to TRM. Solid media were obtained by adding 16 g/L of agar for *E. coli* and 10 g/L of phytagel for *T. barophilus*. To monitor archaea abundances in liquid media by flow cytometry, an Agilent Advanteon cytometer equipped with a laser with an excitation wavelength of 488 nm was used. Samples (200 μL of culture) were fixed with glutaraldehyde (0.5% final concentration). After 15 min at 4 °C, the samples were stored at −80 °C until analysis. The thawed samples were then diluted from 10 to 1000-fold with autoclaved 0.2 μm filtered salted milliQ water solution (NaCl 20 g/L) and stained with the nucleic acid specific dye SYBR®Green I (Invitrogen-Molecular Probes; final concentration: 1) for 15 min at room temperature in the dark. The trigger was set on green fluorescence. Samples were delivered at a rate of 5 μL/min and analyzed for at least 1 min. Salted milliQ water solution and non-inoculated TRM medium were used as negative controls. The data were processed using the NovoExpress tool (v1.4.1).

### Plasmids construction

Primers used are listed in Supplementary Table 2. Deletion of *hibA* (TERMP_00697) was performed using plasmid pRD507. This plasmid was constructed by fusion of PCR products obtained using primer pairs 635/636 and 637/638, followed by amplification with primers 635/638. The resulting fusion fragment was inserted, after digestion by *Bgl*II and *Kpn*I, into pUPH previously digested by *Kpn*I and *Bam*HI.

### Transformation methods and strains verification

The transformation of *T. barophilus* (genetic strain Δ*TERMP_00517* will be referred to as wild type [WT]^40^) was performed as previously described^53^ using 0.2–2 µg of plasmid. Verification of the deletion was performed by PCR using primer pairs 7/8 and 398/399 to ensure that the non-replicative plasmid used to construct the mutant was not retained in the cell, and with primers 639/640 to confirm the deletion of *hibA*. In addition, Whole Genome Sequencing also confirmed the deletion of TERMP_00697 in the mutant strain.

### Most probable number assays

The concentration of cells capable of resuming growth after nutrient stress was determined using the serial dilution culture method, specifically the three-tube Most Probable Number (MPN) assay^54^. Cultures of 50 mL of both strains were started from precultures and incubated at 85 °C until the stationary phase. Cells were then centrifuged for 10 min at 10,000 × *g*, and washed with carbon-free TRM to remove residual nutrients. After washing, cells were resuspended in 15 mL of TRM lacking carbon sources, and cell concentration was determined by optical cell counting. Subsequently, a 50 mL volume of starvation TRM was inoculated to obtain an initial density of 2.5 × 10^6^ cells/mL. These cultures were then incubated at 85 °C, and after 0, 5, 24, 48, and 120 h, 6 serial tenfold dilutions in a TRM-rich medium with colloidal sulfur to assess the ability of the WT and mutant strains to resume growth in rich medium after stress exposure. The results are compared with the MPN statistical table^54^ to estimate the concentration of viable cells in the stressed cultures. This method provides an estimate of the number of viable cells with a defined margin of error.

### Sucrose gradient fractionation of whole-cell extracts

*T. barophilus* WT and ∆*hibA* cellular extracts were prepared from cells cultivated anaerobically at 85 °C under atmospheric pressure (0.1 MPa) in TRM medium^52^, and collected in the stationary phase (15 h; 0.5 to 1.10^8^ cells/mL) by filtration (0.45 µm) and centrifugation (26,000 × *g* for 5 min at 4 °C). Following a washing step in 1X PBS buffer, 250 mg of cells were re-suspended with 500 mL of SEC100 buffer (20 mM HEPES, pH 7.5; 100 mM NH_4_Cl; 20 mM Mg acetate; 2.5 mM DTT) containing an EDTA-free protease inhibitor cocktail (cOmplete^TM^, Roche). Whole-cell extracts were prepared by disrupting the cells with one volume of glass beads (>100 µm, Sigma G4649) using two cycles of 2 ×30 s at 5500 rpm at 4 °C (Precellys homogenizer). The glass beads were then removed by centrifugation at 5000 × *g* for 5 min. Cell lysates were cleared by centrifugation at 16,000 × *g* for 10 min at 4 °C, followed by filtration through a 0.45 µm Ultrafree-MC HV centrifugal filter (Millipore). The cell extracts were quantified by Bradford protein assay. A total of 1.5 mg of protein was layered onto a linear 10–50% sucrose gradient in SEC100 buffer. The gradients were then centrifuged at 220,000 × *g* in a Beckman SW41Ti rotor for two and a half hours at 4 °C. The sucrose gradients were analyzed on a density gradient fractionation system (ISCO UA-6 detector/Brandel Foxy Gradient) with continuous monitoring at 254 nm, allowing the various ribosomal peaks to be resolved. Data was acquired using PeakTrack software from Teledyne. Excel was then used to create the profiles.

### Production and purification of *P. abyssi* (His)_6_-HibA

HibA with an N-terminal (His)_6_ tag was produced in *E. coli Rosetta*. Cultures (1 L) were done at 37 °C in 2xYT medium containing ampicillin (50 mg/L) and chloramphenicol (34 mg/L). When OD_600nm_ reached 0.7, expression was induced with 0.5 mM of IPTG, and the culture was transferred to 18 °C. Cells were lysed by sonication in buffer A (10 mM MOPS-NaOH, pH 6.7; 500 mM NaCl; 3 mM β-mercaptoethanol) supplemented with PMSF and benzamidine. The clarified lysate was incubated for 5 min at 65 °C to precipitate *E. coli* proteins. HibA was then purified by Talon Metal affinity chromatography (Clontech) followed by size exclusion chromatography on a Superdex 200 column (10 mm × 300 mm; Cytiva) equilibrated in buffer A. The N-terminal domain of HibA was produced thanks to the introduction of a stop codon in position 272 and purified as above. We also produced a variant of HibA where the two archaeal-specific insertions in the C-terminal domain have been deleted (∆_283_SAL_285_ and _324_KFRGVHL_330_ replaced by AA). This variant HibA∆∆ was purified using the same protocol as for the wild-type protein, except that the clarified lysate was not heated.

### Size-exclusion chromatography of ribosomal complexes

A total of 500 pmol of Pa-(His)_6_-HibA were incubated with 35 pmol of *P. abyssi* ribosomal subunits^55,56^ (30S, 50S or a 1:1 30S:50S mixture) for 45 min at 51 °C in 50 µL of ribosome buffer (20 mM Hepes, pH 7.5; 100 mM NH_4_Cl; 10.5 mM MgAc; 3 mM β-mercaptoethanol). The mixture was then loaded onto an Agilent Bio-SEC5 column (1000 Å) equilibrated with ribosome buffer.

### In vitro reconstitution of HibA:ribosome complexes

30S and 50S subunits, purified as described in refs. ^55,56^, were activated for 5 min at 51 °C. A total of 10 pmol of each subunit was incubated for 1 h at room temperature with 50 pmol of (His)_6_-HibA in a total volume of 12 µL of ribosome buffer for 1 h at room temperature. Samples were then diluted to A_260_ = 4.2 before deposition onto cryo-EM grids. For the HibA-ATP and HibA-AMP datasets, 5 mM of the respective nucleotides and 5 mM MgCl_2_ were added at the final step of HibA:ribosome incubation.

### Purification of 70S ribosomes from *P. abyssi* cell extracts

*P. abyssi* cells (DSM 25543)^57^ were grown anaerobically at 90 °C and harvested at the end of the exponential growth phase (Td = 33 min) as previously described^55^. Cultures were then exposed to an aerobic environment and cooled for 1 h in a 4 °C cold room before centrifugation. Cell pellets were frozen in liquid nitrogen and stored at −80 °C. To purify ribosomes, cells (0.3 g) were resuspended in 660 µL of ribosome buffer supplemented with RNaseOUT inhibitor (Invitrogen). After the addition of an equal volume of 5 µm glass beads (Sigma), cells were vortexed for 5 min. The lysate was centrifuged for 5 min at 5000 × *g* and further clarified by two additional centrifugation steps at 19,000 × *g* (10 min and 5 min) The extract was then fractionated on a 10–40% sucrose gradient centrifuged for 2h30 at 36,000 rpm (SW41 rotor, Beckmann). Fractions corresponding to 70S ribosomes were pooled. Sucrose was removed by successive dilution/concentration steps using a Vivaspin 500 PES 30 kDa concentrator, ensuring a residual sucrose concentration below 1% (w/v). Samples were diluted to A_260_ = 3 for cryo-EM grids preparation.

### In vitro translation

HiBiT peptide sequence was cloned into pET15b between NdeI and XhoI restriction sites (Supplementary Table 2). A total of 2.5 µg of Xho1-linearized plasmid was then transcribed using T7 RNA polymerase, and the transcription product was purified using a Mono-Q column (Cytiva). In vitro translation assays were made by mixing 37.5 ng HiBiT mRNA, 3 mM ATP, 2 mM GTP, 3 mM spermine, 0.5 mM of each aminoacid and 2 µL of *P. abyssi* lysate prepared as described^55,56^. Final concentrations of Mg(OAc)_2_ and NH_4_Cl were 10 mM and 133 mM, respectively. Reactions were incubated at 80 °C for 10 min in a PCR machine and then cooled on ice. For each reaction, an 8 µl aliquot was added to 20 µL of LgBiT buffer (20 mM HEPES pH 7.5, 50 mM KCl and 10% glycerol) containing NanoGlo HiBiT lytic substrate (1:50 dilution, Promega) and LgBiT protein (1:100 dilution, Promega). Luminescence was measured in a GloMax® 20/20 Luminometer (Promega).

### Cryo-EM analysis

Samples (3.4 μL) were spotted onto grids (Quantifoil R2/1 Copper 300 mesh with an additional 2 nm continuous carbon layer) at 20 °C and 90% humidity for 10 s. Each sample was vitrified by plunging into liquid ethane at −182 °C, after 1.2 s of blotting using a Leica EM-GP plunger. Conditions were optimized using a Titan Themis cryo-microscope (Thermo Fisher) at the CIMEX facility of Ecole Polytechnique. Final datasets were collected on Titan Krios cryo-microscopes (Thermo Fisher) available at the ESRF (CM01) or SOLEIL (Polaris) facilities (Supplementary Table 3). Data processing was conducted in Cryosparc 4^58^. The same workflow was used for all datasets described in this study. Briefly, initial 2D templates were generated via blob picking and used to train a Topaz model^59^. Topaz picks were then used to generate three initial 3D models, which were refined through heterogeneous refinement. Further 3D classifications, global or focused, were performed before final homogeneous refinements. Subsequent global and focused 3D classifications were carried out prior to final non-uniform refinements. Flexible regions were further refined using 3D Flex^60^ to improve local map quality. A detailed description of all processing steps is provided in Supplementary Figs. 1, 10, 12 and 13. Particle orientation diagnostic and local resolution cryo-EM maps are shown in Supplementary Fig. 24.

Models were built in Coot^61^ and refined in Phenix^62^. 3D structures of the 30S from *P. abyssi*^55,56,63^ and that of the 50S from *T. kodakarensis* and *P. furiosus*^64,65^ were used as starting models. Examples showing the quality of the cryo-EM map for rRNA modified nucleotides (Supplementary Table 4), magnesium ions and HibA environment are shown in Supplementary Figs. 25 and 26. Water molecules were only added in the HibA-PTC reference structure (2.1 Å, PDB 9SRE). rRNA secondary structure diagrams are given in Supplementary Figs. 27 and 28. They have been updated according to the 3D structures. All structural figures were drawn using ChimeraX^66^.

### Isothermal titration calorimetry

Before ITC measurements, HibA N-terminal domain was dialyzed against buffer B (10 mM MOPS-NaOH, pH 6.7; 50 mM NaCl; 3 mM β-mercaptoethanol) containing 2 mM MgCl_2_. Titrations curves were obtained using a MicroCal ITC200 apparatus (Malvern). The protein (202 µL) was titrated through injections (4 µL) of ATP (2.48 mM), ADP (2.45 mM), or AMP (0.76 mM) solutions in buffer B containing 3 mM MgCl_2_. Concentrations of proteins were 194 µM (ATP, ADP, GTP, and GMP titrations) or 100 µM (AMP titration). Typical titration experiments are shown in Supplementary Fig. 11. Dissociation constants and stoichiometries were deduced from least-square fits of measured heat values to standard binding isotherms using Origin (OriginLab). Reported results are mean ± s.d. from three (ATP or AMP) or two (ADP) experiments. Titration experiments with GTP or GMP (1 mM in the syringe) did not cause any measurable heat change (Supplementary Fig. 11).

### Genome databases

Two genome databases covering the diversity of archaea were assembled: a minimal database with 388 genomes (Arc388) and a larger one with 4026 genomes (Arc4026). Arc388 was used for phylogenetic analyses, while Arc4026 was used for distribution analyses. All archaeal genomes available in NCBI (2024/01/16) were collected, as well as genomes from other published datasets, including Earth, ocean, soil, glacier, and termite MAG catalogs^67–71^. To build the Arc4026 database, these genomes were dereplicated at 92% ANI using dRep^72^, and only genomes with more than 80% completeness and less than 5% contamination according to CheckM were retained^73^. The Arc388 database is a manually curated sub-selection of genomes covering the main archaeal lineages. For Bacteria we used a previously published database of 1067 genomes covering all major lineages^74^.

### Phylogeny and distribution of HibA, potential alternative hibernation factors

All sequences were aligned with MAFFT L-INS-i^75^ and trimmed with BMGE-1.12^76^ using the BLOSUM30 substitution matrix. All maximum- likelihood trees were built with IQ-TREE 2.0.6 using the TESTNEW option to define the best model^77^. Tree visualization, coloring, and protein presence/absence mapping were performed with ITOL^78^.

Pa-HibA was used as a seed to search for the closest sequences in Arc4026. From the C-terminal region (corresponding to the RaiA domain of HibA) of these sequences, we built an HMM domain using HMMBUILD (hmmer v3.3.2 [http://hmmer.org/]). Additional HibA sequences were identified in Arc4026 using the HibA-Cter HMM profile (HMMSEARCH) and incorporated for iterative refinements of the profile. Specificity of the hits was assessed by identifying additional conserved domains, including the presence of the CBS module, and by examining Alphafold2-predicted protein structures available in Uniprot (https://www.uniprot.org/) for the most divergent sequences. Shorter sequences lacking the CM module but displaying the HPF/RaiA fold were retained.

The HibA alignment was also truncated at the C-terminal region to generate a HibA-Nter HMM profile corresponding to the CBS module. Using this profile, additional proteins containing a CBS module were identified in the Arc388 database. All proteins with an E-value lower than 10^−28^ were retained. Most proteins with a higher E-value are composed of a single tandem of CBS instead of two and can be associated with diverse alternative conserved domains. Selected hits included the Dri hibernation factor, formed by a fusion of two CBS modules. Dri sequences were split and annotated as Dri Nter and Dri Cter. After alignment trimming, a maximum-likelihood tree was built and used to delineate paralogous protein families corresponding to CBS modules (called CM-1 to CM-6), in addition to the family formed by the CBS module region of HibA. The description of HhpR (MJ0729), belonging to the CM-3 family, was published recently^41^ and its genome was not part of the Arc388 database. The sequence of this protein was thus not retrieved from our analysis but directly added to the CM alignment.

HMM profiles were built for each of these families and used to determine their distribution in the Arc4026 and Bac1067 databases. Alternative hibernation factors known to occur in bacteria (Etta, SRA, ElaB/YqjD, RMF, RsfS, and Balon) and Eukaryotes (Stm1, IFRD2, Lso2, MDF1, MDF2, and Dapl1) were searched using available COG or Pfam HMM profiles or by BLASTp searches using the originally characterized proteins. For Balon, an HMM profile was generated using proteins identified in Bacteria^14^. With the exception of Balon, few or no significant hits were obtained. Potential archaeal Balon proteins were distinguished from homologous aeRF1 and pelota proteins by identifying sequences lacking the NIKS and GGQ motifs of aeRF1 and forming a distinct monophyletic lineage in an ML phylogeny. All genomes encoding a putative archaeal Balon protein also have aeRF1, and sometimes a pelota protein. Identified hibernation factors were mapped on a tree containing all archaea from the Arc4026 database. Markers used to build this tree correspond to the Phylosift dataset^79^ and five additional markers as described in ref. ^80^.

Sequences containing the HPF/RaiA domain and the lHPF CTD were searched in the Bac1067 database using the COG1544 and PF16321 HMM profiles, respectively. To identify conserved domains fused to HPF/RaiA, we used CD-search^81^ on proteins longer than 150 aa lacking the lHPF CTD. Phylogenies of the bacterial lHPF and HPF/RaiA without the addition domain were built with and without the addition of the HibA-Cter domain and HPF/RaiA-like proteins from Archaea.

### Mass spectrometry analysis of the 70S fraction

70S preparations as described in Fig. 1a were diluted in 8 M urea, 100 mM Tris-HCl, pH 8.5, to obtain a final urea concentration of 6 M. Proteins were reduced using 5 mM Tris(2-carboxyethyl)phosphine for 30 min at room temperature. Alkylation of the reduced disulfide bridges was performed using 10 mM iodoacetamide for 30 min at room temperature in the dark. Proteins were then digested in two steps, first with 500 ng r-LysC Mass Spec Grade (Promega) for 4 h at 30 °C, and then the samples were diluted below 2 M urea with 100 mM Tris HCl pH 8.5, and 500 ng Sequencing Grade Modified Trypsin was added for the second digestion overnight at 37 °C. Proteolysis was stopped by adding formic acid (FA) at a final concentration of 5%. The resulting peptides were cleaned using AssayMAP C18 cartridges on the AssayMAP Bravo platform (Agilent) according to the manufacturer’s instructions. Peptides were eluted using 50% acetonitrile (ACN), 0.1% formic acid. Peptides were concentrated to dryness and resuspended in 2% ACN/0.1% FA just prior to LC-MS injection.

LC-MS/MS analysis was performed on a Q ExactiveTM Plus Mass Spectrometer (Thermo Fisher Scientific) coupled with a Proxeon EASY-nLC 1200 (Thermo Fisher Scientific). Two hundred and fifty ng of peptides was injected onto a home-made 22 cm C18 column (1.9 μm particles, 100 Å pore size, ReproSil-Pur Basic C18, Dr. Maisch GmbH, Ammerbuch-Entringen, Germany). Column equilibration and peptide loading were done at 900 bars in buffer A (0.1% FA). Peptides were separated with a multi-step gradient from 3 to 6% buffer B (80% ACN, 0.1% FA) in 3 min, 6 to 31% buffer B in 82 min, 31 to 62% buffer B in 20 min at a flow rate of 250 nL/min. Column temperature was set to 60 °C. MS data were acquired using Xcalibur software using a data-dependent method. MS scans were acquired at a resolution of 70,000, and MS/MS scans (fixed first mass 100 m/z) at a resolution of 17,500. The AGC target and maximum injection time for the survey scans and the MS/MS scans were set to 3E6, 20 ms and 1E6, 60 ms, respectively. An automatic selection of the 10 most intense precursor ions was activated (Top 10) with a 45 s dynamic exclusion. The isolation window was set to 1.6 m/z and the normalized collision energy was fixed to 28 for HCD fragmentation. We used an underfill ratio of 1.0% corresponding to an intensity threshold of 1.7E5. Unassigned precursor ion charge states as well as 1, 7, 8, and >8 charged states were rejected, and peptide match was disabled.

### Protein identification

Acquired Raw data were analyzed using MaxQuant software version 2.1.4.0^82^ using the Andromeda search engine^83^. The MS/MS spectra were searched against the P. abyssi Uniprot reference proteome database (1891 entries).

All searches were performed with oxidation of methionine and protein N-terminal acetylation as variable modifications and cysteine carbamidomethylation as a fixed modification. Trypsin was selected as a protease allowing for up to two missed cleavages. The minimum peptide length was set to 5 amino acids, and the peptide mass was limited to a maximum of 8000 Da. One peptide unique to the protein group was required for the protein identification. The main search peptide tolerance was set to 4.5 ppm and to 20 ppm for the MS/MS match tolerance. The second peptides were enabled to identify co-fragmentation events. The false discovery rate for peptide and protein identification was set to 0.01.

### Reporting summary

Further information on research design is available in the Nature Portfolio Reporting Summary linked to this article.

## Supplementary information

Supplementary information

Description of Additional Supplementary Files

Supplementary Data 1

Reporting Summary

Transparent Peer Review file

## Source data

Source Data

## Data Availability

The atomic models and cryo-EM maps have been deposited in the Protein Data Bank. The PDB and EMDB codes are as follows, 70S-HibA-PTC: 9SRE, EMDB 55139; 70S-HibA-PTC-wo-E-tRNA: 9SRC, EMDB-55137;70S-HibA-uL5: 9SRD, EMDB-55138; 70S-HibA-L1 stalk: 9T7H; HibA-CBSmodule-L1 stalk: 9SRF, EMDB-55140; 70S-HibA-L1 stalk-SBDS: 9SRB, EMDB-55136; 70S-HibA-SD-antiSD: 9SRA, EMDB-55135; HibA-ATP: 9SR9, EMDB-55134. The mass spectrometry proteomics data have been deposited to the ProteomeXchange Consortium via the PRIDE partner repository with the dataset identifier PXD073243. Source data are provided with this paper.
