# Supplementary material for: A family of ribosome hibernation factors widespread in Archaea
Source: Nat Commun. 2026 Apr 27;17:5751. doi: 10.1038/s41467-026-72341-8 (PMC13324472; doi:10.1038/s41467-026-72341-8)
Supplement: Supplementary file 1 — Supplementary information [file 41467_2026_72341_MOESM1_ESM.pdf]

A family of ribosome hibernation factors widespread in Archaea

Clément Madru[2#], Gabrielle Bourgeois[2#], Rémi Dulermo[1#], Régine Capeyrou[3], Gwendoline Joncour[1], Karima Figuigui[1], Magalie Duchateau[4], Julia Chamot-Rooke[4], Claire Duboc[1], Stéphane l'Haridon[1], Logan Mc Teer[1], Marta Kwapisz[3], Béatrice Clouet-d'Orval[3], Marie Bouvier[3], Yves Mechulam[2], Guillaume Borrel[5*], Emmanuelle Schmitt[2*] & Didier Flament[1*]

Supplementary information

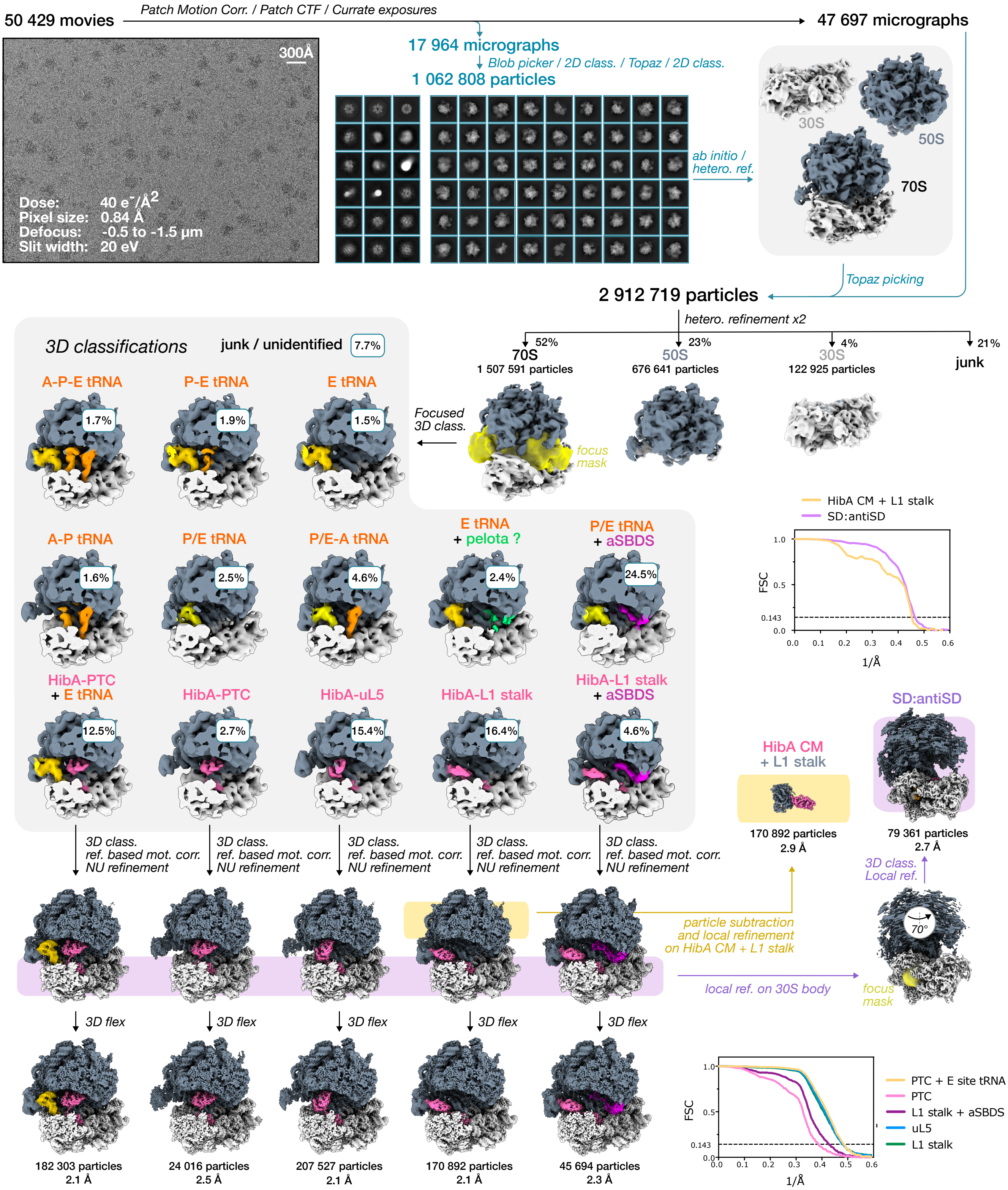

Supplementary fig. 1: Cryo-EM structure determination of ribosomes from cell extracts

Data processing was performed in CryoSPARC v4.1. Following motion correction and CTF estimation, exposures were filtered based on CTF fit (<5 Å) and ice thickness. An initial subset of 17,964 images was processed by blob-based particle picking and 2D classification to retain ribosome classes, which were used to train Topaz. Particles repicked with the trained model underwent iterative 2D classification, ab initio reconstruction, and heterogeneous refinement to remove contaminants, and the resulting clean subset was used to retrain Topaz for large-scale picking. Using this model, particle picking across the entire dataset yielded 2,912,719 particles (box size of 480 pixels) which were classified in 3D against ribosome and junk references. The resulting 70S ribosomes were subjected to focused classification on the tRNA-binding sites, and HibA-bound classes were refined with reference-based motion correction to generate final maps using 3D Flex, with the masks shown in supplementary fig. 10. To enhance resolution of the HibA CM/L1 stalk complex, particle subtraction with a mask excluding the rest of the ribosome mask was followed by local refinement centred at the L1 stalk. In parallel, HibA-bound particles were aligned on the 30S body, classified with a mask over the SD:anti-SD region, and the resulting particles were refined with a 30S body mask.

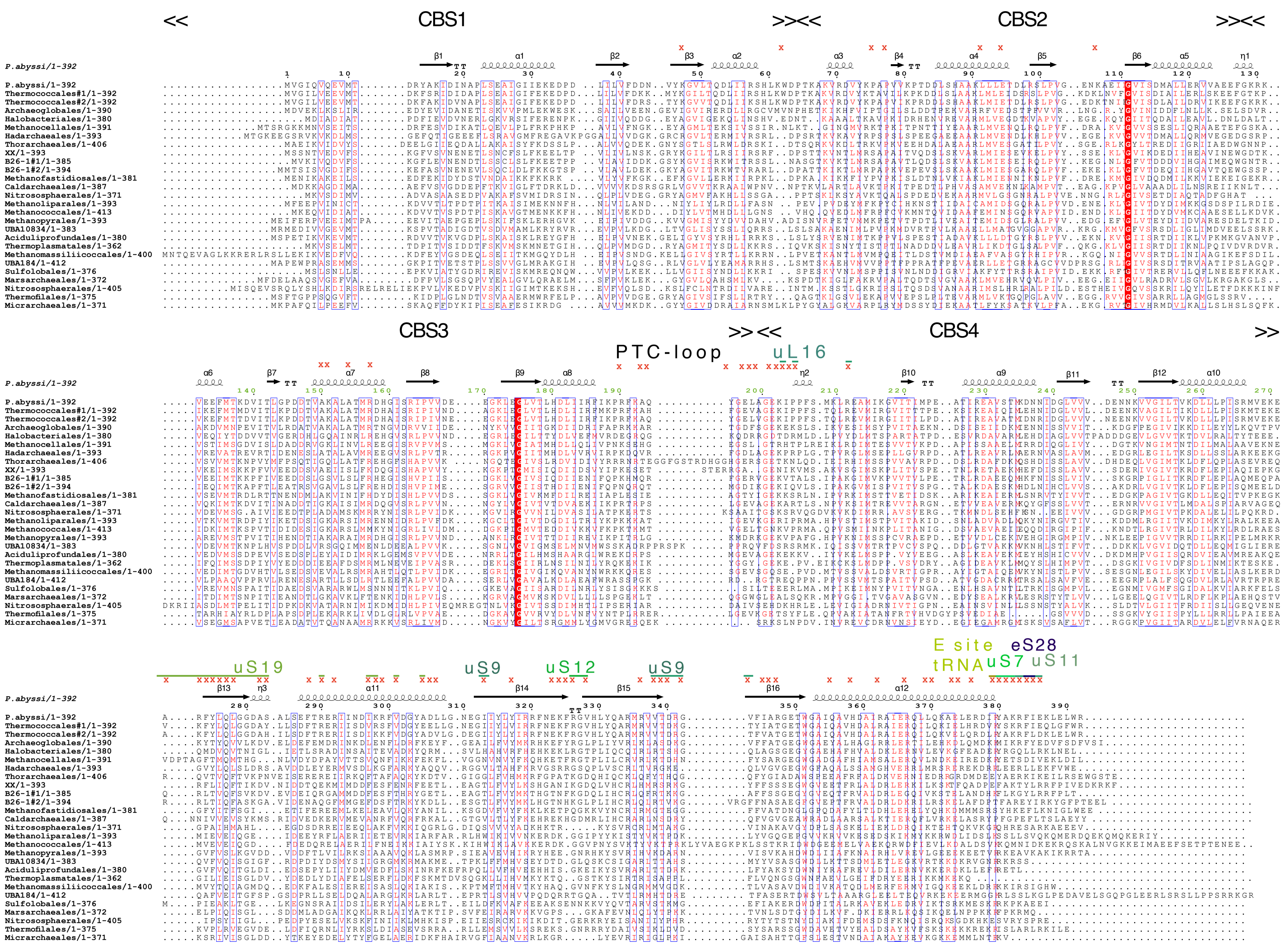

Supplementary fig.2: Sequence alignment of HibA from various Archaea.

HibA residues interacting with the ribosome are indicated with a red star. When a ribosomal protein is involved in the interaction, it is indicated above the red star.

a

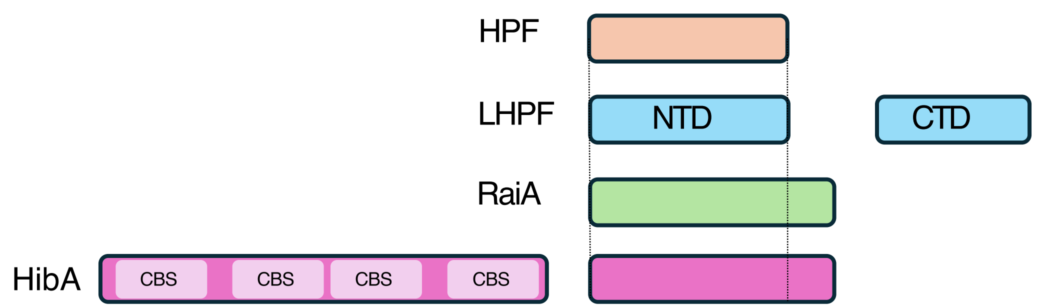

b

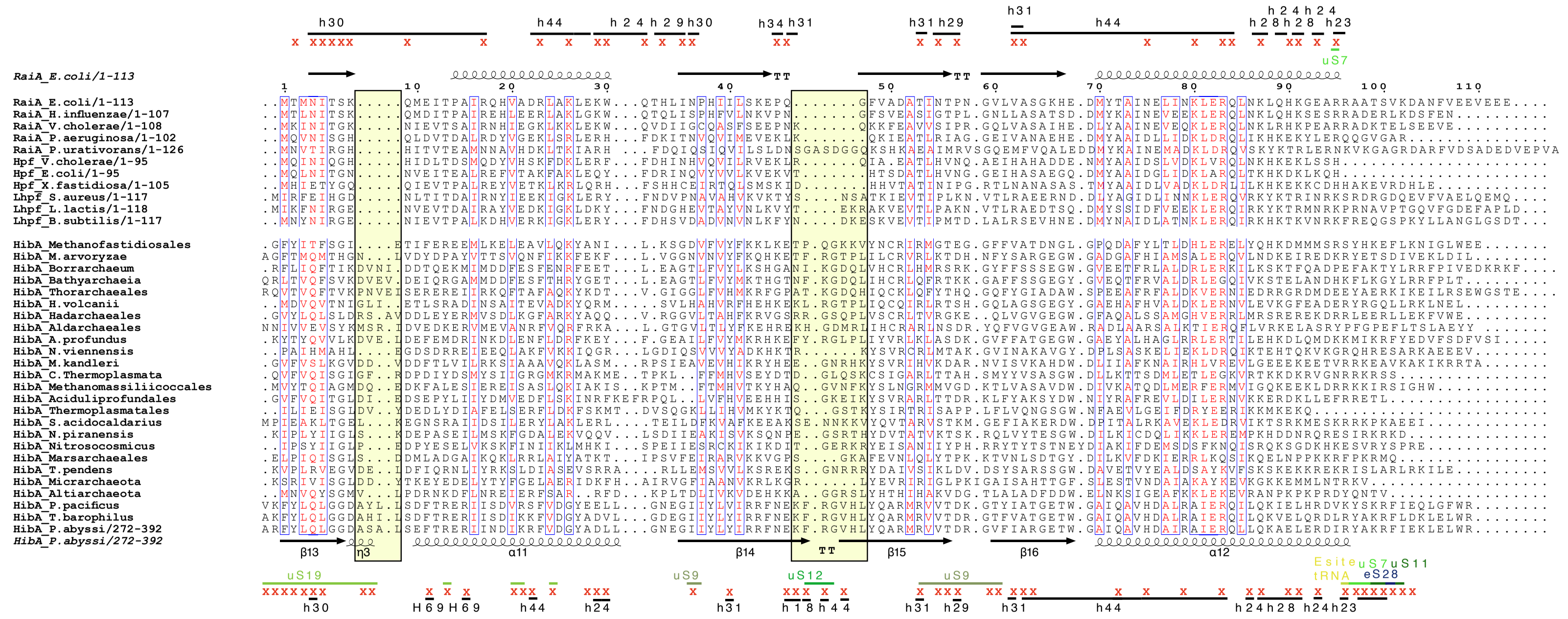

Supplementary fig.3: HibA shares a common domain with bacterial hibernation factors involved in 30S subunit binding.

a. Schematic representation of domain arrangement of HibA and bacterial hibernation factors. Vertical dashed lines delineate homologous regions ; horizontal dashed lines indicate variable length linker region between N- and C-terminal domains of LHPF and between N- and C-terminal domains of HibA. b. Structural alignment of C-terminal domains of HibA from diverse archaeal species with representative members of bacterial hibernation factor families. The reference structures are E. coli RaiA (4V8I) and P. abyssi HibA (present work). RaiA or HibA residues interacting with the rRNAs are indicated with dark dashes and labelled, those interacting with a ribosomal protein are indicated with colored dashes and labelled. The figure shows that most of the contact points of the hibernation factors with ribosomal RNA are conserved however P. abyssi HibA can be distinguished from RaiA homologues by two insertions (highlighted by yellow boxes). These insertions are involved in numerous contacts with ribosomal proteins (see text, Fig. 2 and Supplementary fig. 5 and 6). Note that RaiA from Psychrobacter urativorans has an insertion between the second and third b-strand. However, this region does not form a b-hairpin (PDB code 8RD8) as in HibA. H is used for helices of the LSU, h is used for helices of the SSU

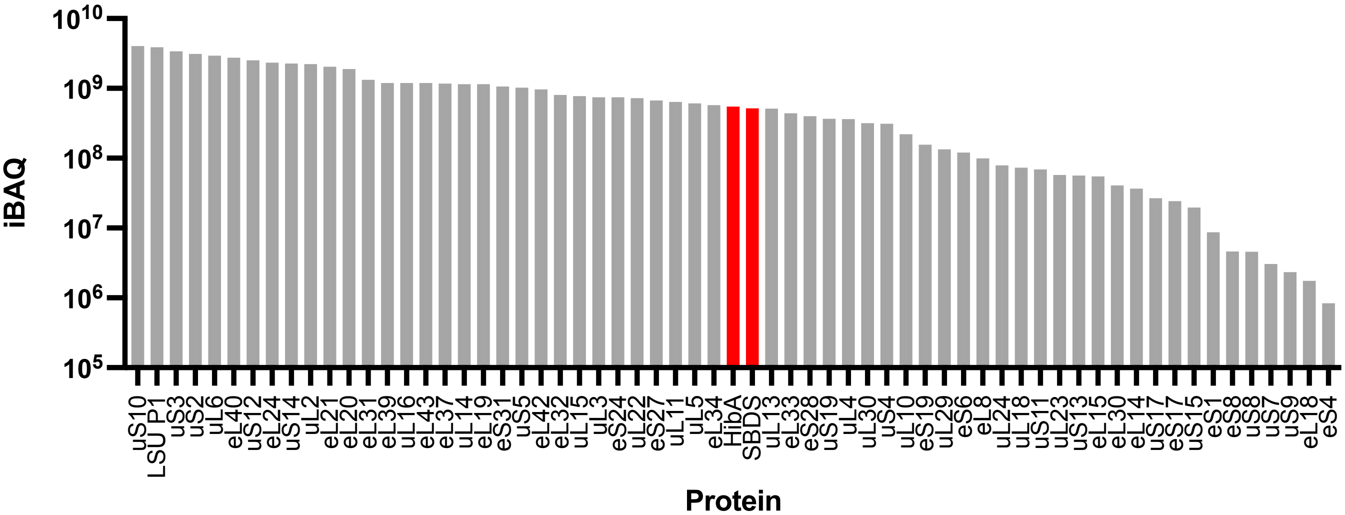

Supplementary fig. 4: identification of ribosomal proteins, HibA and aSBDS by nanoLC-MS/MS analysis

Mass spectrometry analysis (see methods) was performed on the 70S sample shown in Fig. 1A. Data processing was performed with MaxQuant software against a database containing the P. abyssi proteome (Uniprot# UP000000810). iBAQ intensities are plotted in a log scale for ribosomal proteins, HibA and aSBDS.

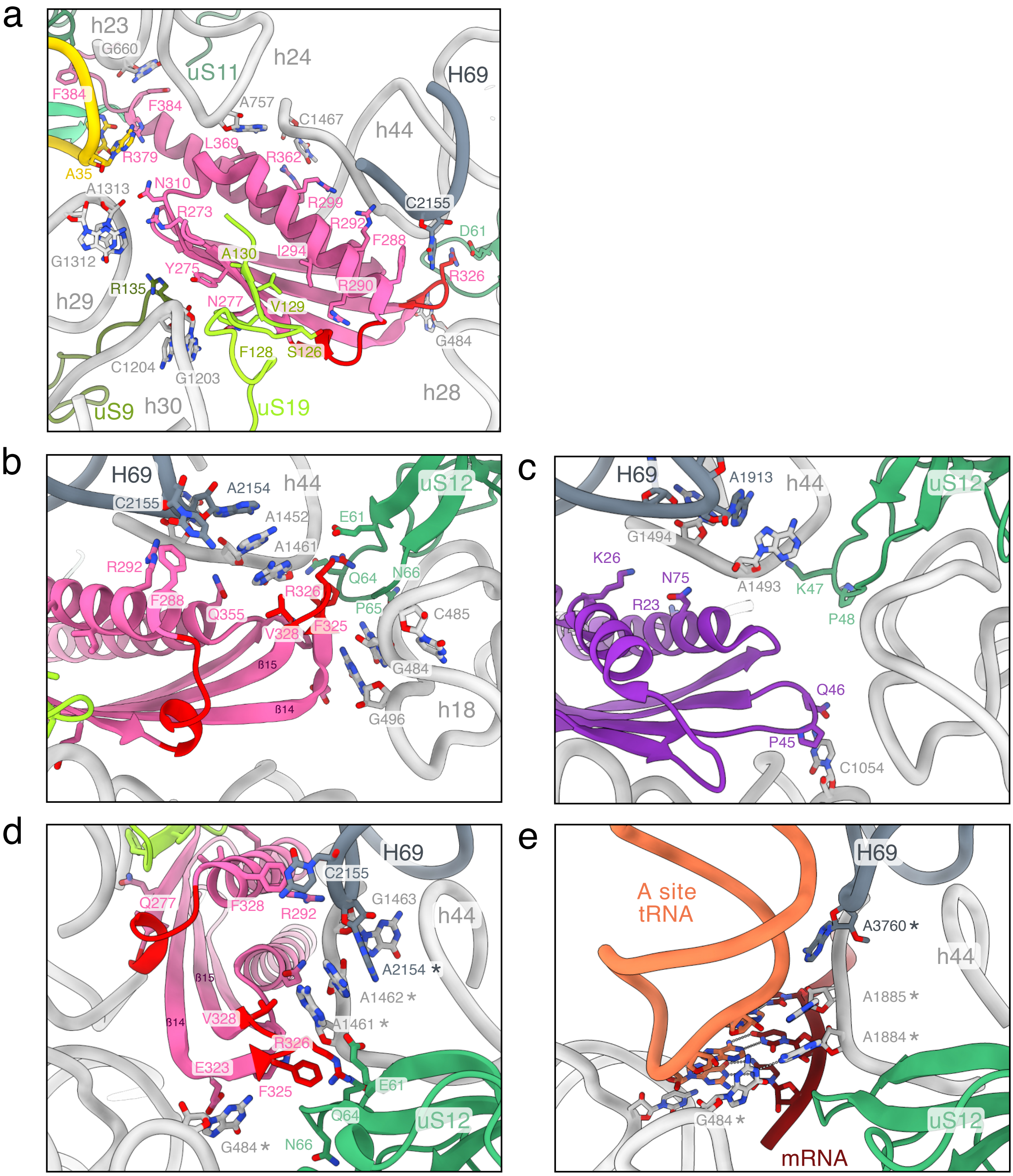

Supplementary fig. 5: Interaction of the C-terminal domain of HibA with uS12 and the B2a bridge at the A site

a. Close-up of the HibA C-terminal domain binding site. Some of the residues involved in ribosome:HibA contacts are shown in sticks. The contacts were calculated with ChimeraX (see also Supplementary Data 1). b. Close-up of the contacts between uS12 (in green), the B2a bridge (H69:h44) and the C-terminal domain of HibA. Only the residues involved in contacts are shown in sticks. The two archaeal-specific insertions are shown in red. c. Structure of E. coli RaiA (PDB 4V8I) bound to the ribosome shown in the same orientation as in view a. Residues involved in contacts between RaiA and the ribosome are shown in sticks. d. Identical to a. but zoomed in on A site. Bases h44-A1461, h44-A1462, h18-G484 and H69-A2154 (corresponding to A1493, A1492, G530 and A1913 E. coli numbering) are indicated by a star. e. Same region but in the structure of human ribosome with A site tRNA and mRNA (PDB 8G61). The two structures were aligned by superimposing the 16S rRNA. The view shows the codon:anticodon interaction and the position of the four bases universally involved in the decoding step. Comparing views c and d shows how HibA interacts with bases important for the decoding mechanism.

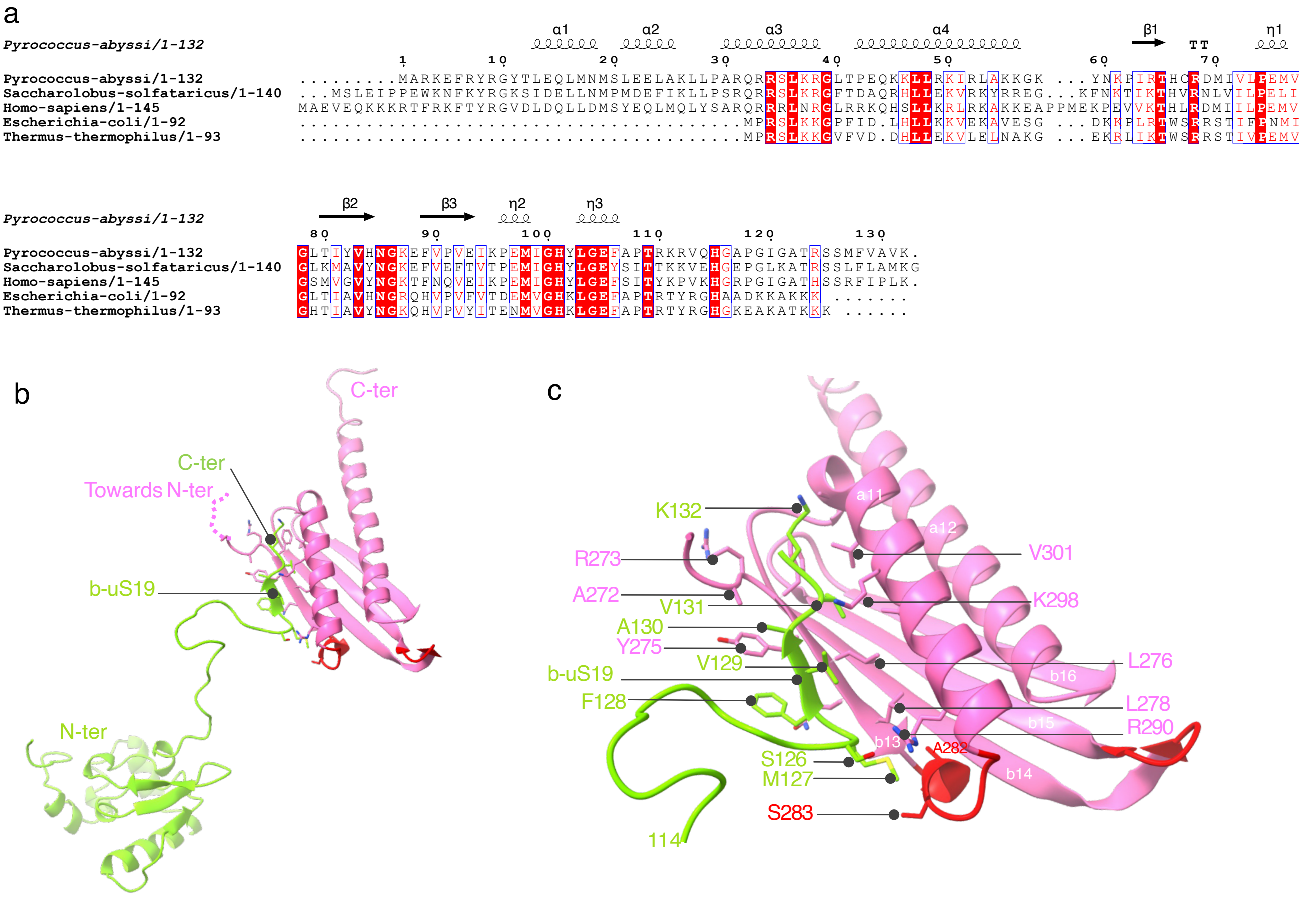

Supplementary fig. 6: Interaction of the C-terminal domain of HibA with uS19

a. Sequence alignment of uS19 from the three domains of life. Eukaryotic and archaeal uS19 have a longer C-terminal tail (8 to 9 residues) than bacterial uS19, see also these studies for larger alignments[4,5]. b. Interaction between uS19 and the C-terminal domain of HibA. The view highlights the contacts of the C-terminal tail of uS19 and the C-terminal domain of HibA. The HibA insertions, as compared to bacterial RaiA or HPF homologs are colored in red. c. Close-up of the contacts between uS19 and the C-terminal domain of HibA.

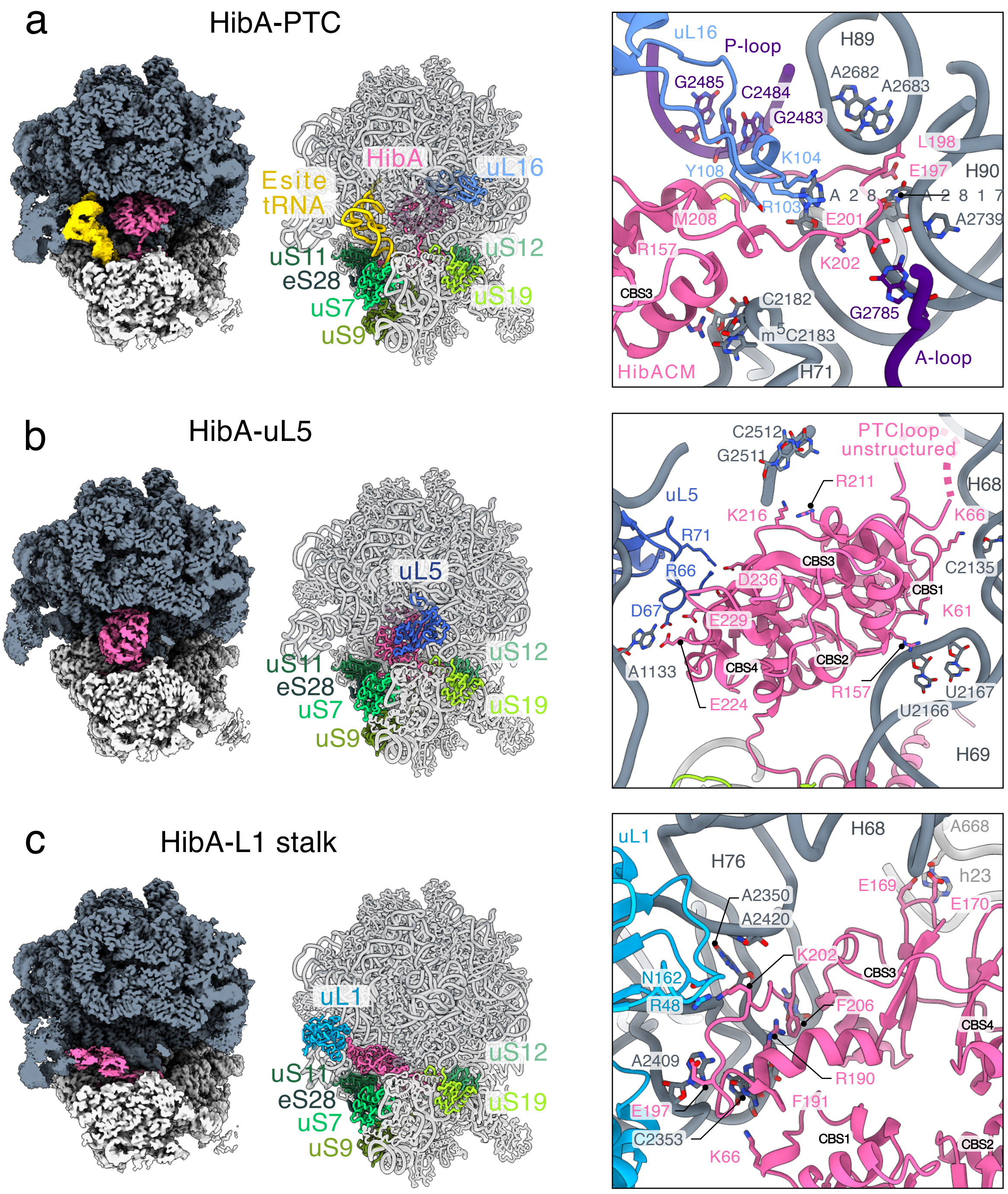

Supplementary fig. 7: Thethree HibA states.

a. HibA-PTC2.1 Å cryo-EM map (left) and structure (right). Thecryo-EM map was clipped to show Hib inside of the ribosome. Hib is shown in pink. Ribosomal proteins interacting with Hib are colored and labeled (right). Theright-hand views show a detail of the interaction of HibA in the PTCregion.
b. HibA-uL5 2.0 Å cryo-EM map (left) and structure (right). Theright-hand views show the interaction of Hib in the B1a-B1b/c bridge region with ul5 and rRNA.
c. HibA-L1 stalk 2.1 Å cryo-EM map (left) and structure (right). The right-hand views show the interaction of Hib in the L1-stalk region with uL1 and rRNA.

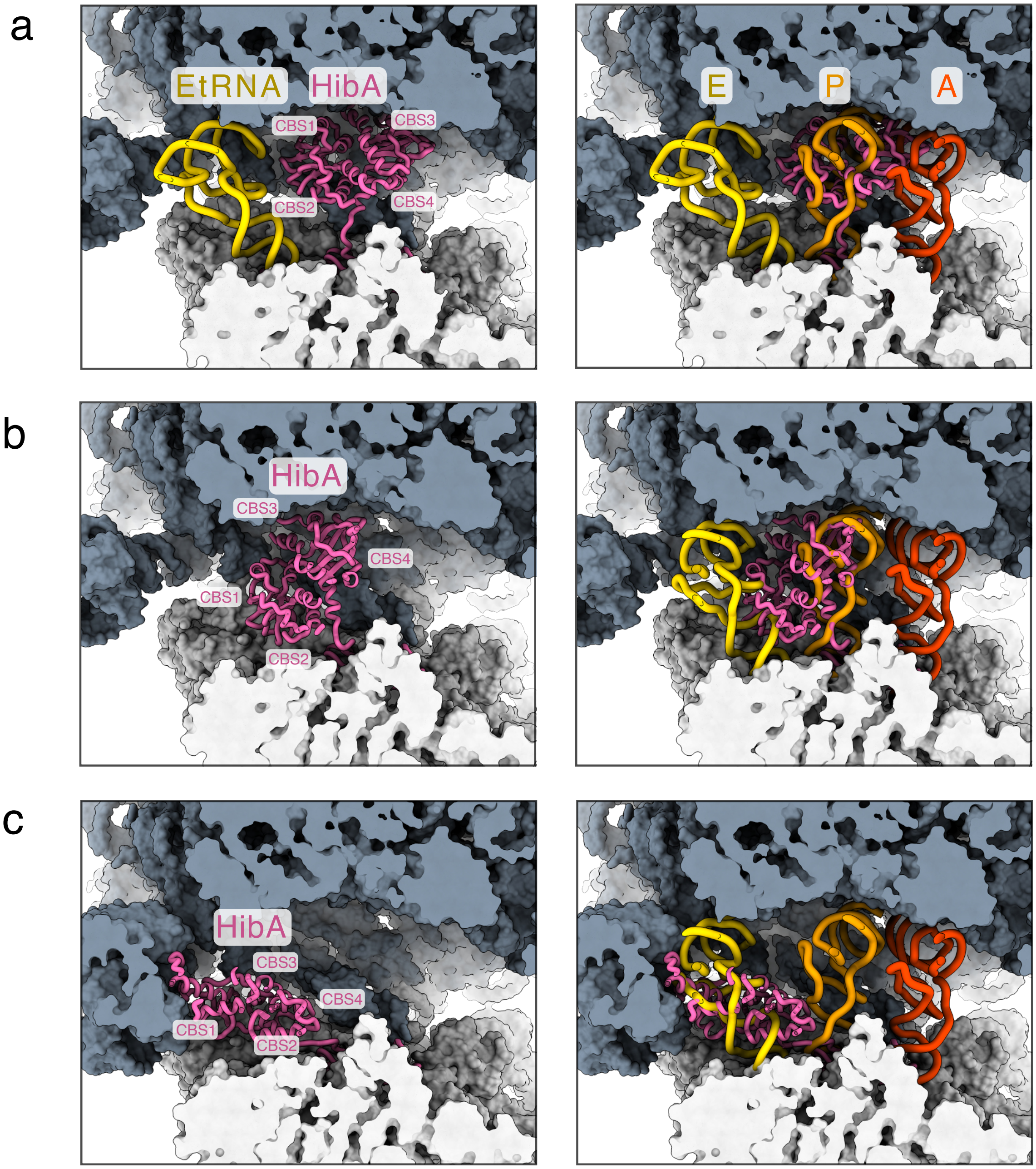

Supplementary fig. 8: comparison of HibA binding sites with tRNA binding sites

The structures HibA-PTC (a), HibA-uL5 (b) and HibA-L1 stalk (c), left panels, were superimposed to the structure of S. acidocaldarius ribosome bound to three tRNAs, right panels (PDB 8HKY).
Color code is as follows: E site tRNA gold, P site tRNA orange, A site tRNA tomato. HibA is in pink.

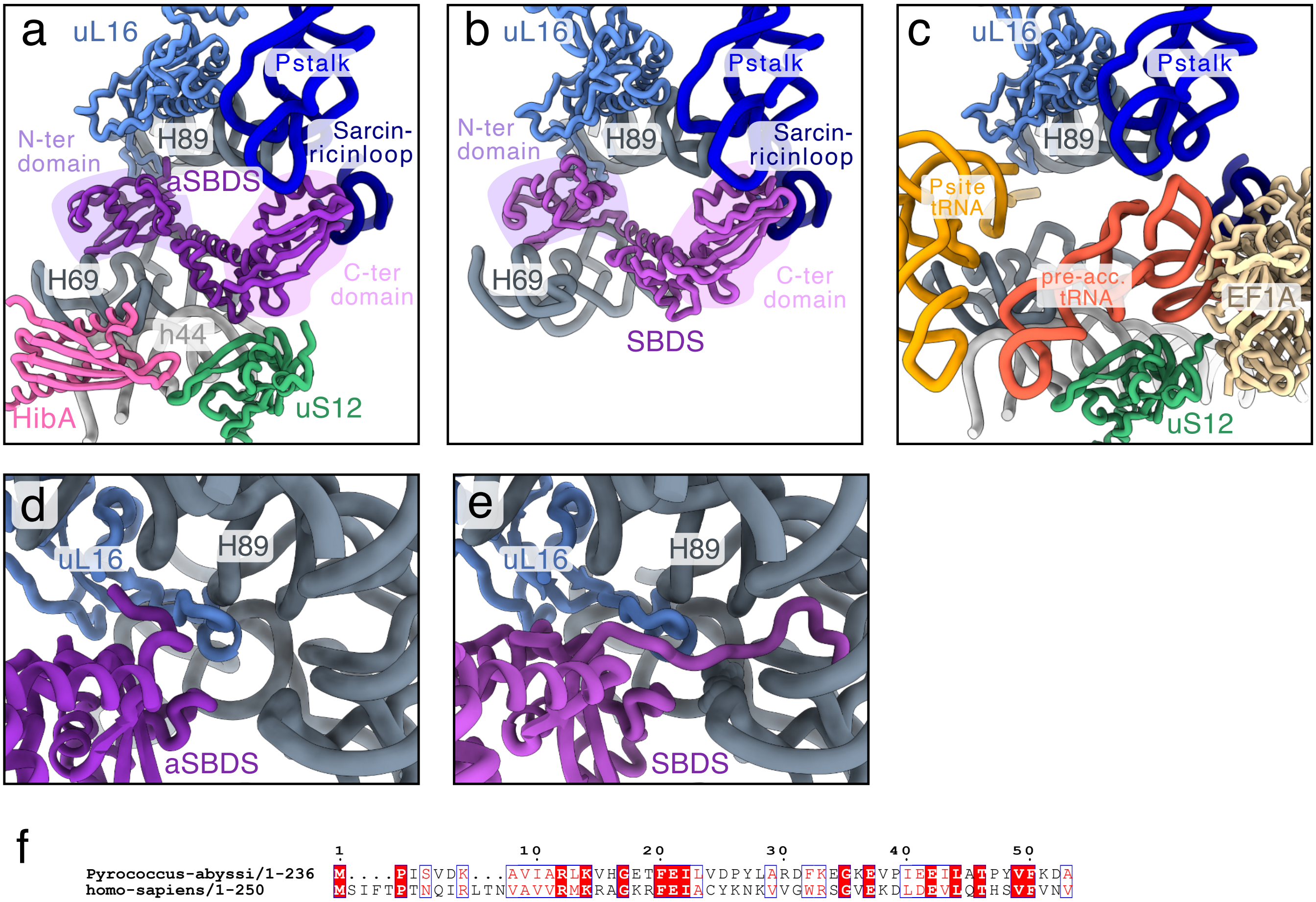

Supplementary fig. 9: Binding site of SBDSto the ribosome

a. Binding of P. abyssi aSBDSto HibA-L1 stalk. rRNAregions and r-proteins interacting with aSBDSare shown and labeled. b. Binding site of Human SBDSto Dictyostelium discoïdeum 50S (PDB 6QKL). Comparison of views a and b shows that the binding sites of SBDSto the ribosome or to the LSU are very similar. c. Same orientation as in view a but for human translating ribosome with a pre-accommodated aa-tRNA:EF1Acomplex (PDB 8G5Z). The view shows that the C-terminal domain of SBDSoccupies the same position as that of the elbow of a pre-accommodated aa-tRNA. d. Same as a but zoomed on the PTC. The view shows the orientation of the N-terminal end of Pa-SBDS. e. Same as b but zoomed on the PTC. The view shows the orientation of the N-terminal end of Human SBDSin the peptide exit tunnel. f. Sequence alignment of Pa-SBDSand human SBDS. Thearchaeal versions of the protein are systematically 6 to 7 residues shorter in their N-terminal end as compared to eukaryotic SBDSversions. This may explain why the N-terminal end of Pa-SBDSis not observed in the peptide exit tunnel (compare views d and e). For clarity, the alignment of P. abyssi aSBDSand Human SBDSsequences is only shown for the first 50 residues.

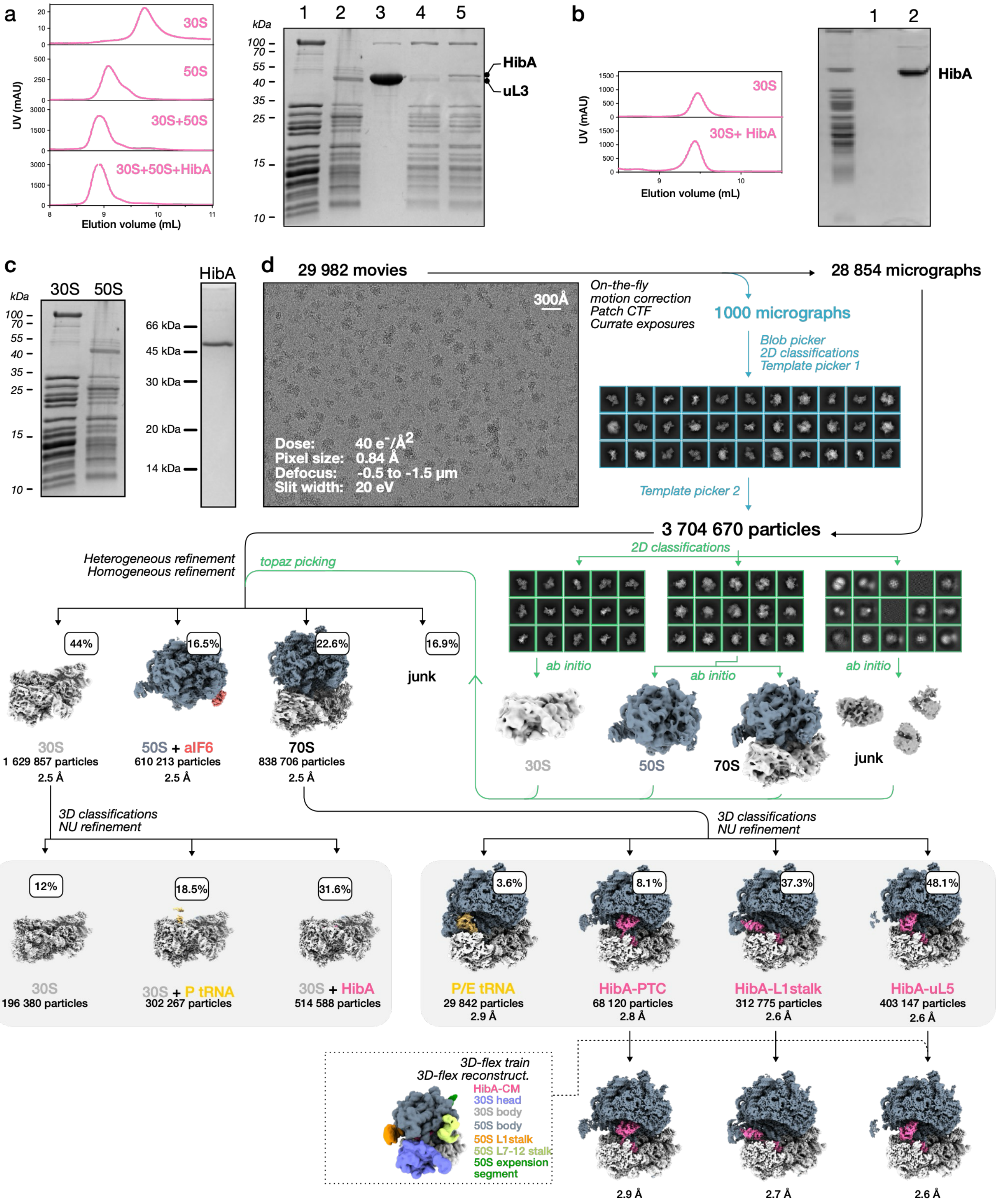

Supplementary fig. 10: Cryo-EM structure determination of in vitro reconstitued hibernating ribosomes.

a. Bio-Agilent Sec5 chromatograms for *P. abyssi* ribosomal subunits-HibA mixtures. SDS-PAGE analysis is shown in the right-hand corner. 1:30S, 2:50S, 3:HibA, 4:elution peak of the 30S+50S mixture, 5: elution peak of the 30S+50S+HibA mixture. The high molecular protein in lanes 1,4,5 corresponds to phosphoenol pyruvate synthase (PEP) that is a usual contaminant of the 30S preparation. The high molecular protein in lanes 3 is a contaminant of HibA **b.** Bio-Agilent Sec5 chromatograms for *P. abyssi* small ribosomal subunit in complex with HibA. SDS-PAGE analysis is shown in the right-hand corner. 1: elution peak of the 30S+HibA mixture. 2: HibA. **c.** SDS-PAGE analysis of purified *P. abyssi* ribosomal subunits and recombinant HibA. **d.** Cryo-EM workflow. Image processing was carried out in cryoSPARC v4.1. After motion correction and CTF estimation, micrographs were screened based on CTF fit (<5 Å) and ice thickness. From an initial subset of 1,000 images, particles were picked using the blob picker and classified in 2D to generate templates. Particles recovered from template-based picking underwent iterative 2D classification, ab initio reconstruction, and heterogeneous refinement. The cleaned particle set was then used to train Topaz, yielding 3,704,670 particles. Extracted particles (box size 480 pixels) were classified in 3D against ribosome and junk references, and the resulting 70S particles were subjected to focused classification on the tRNA binding sites. HibA-bound classes were further refined using non-uniform refinement followed by 3D Flex. Note that free 50S are bound to aIF6, excluding their association with the 30S. The 50S:aIF6 particles correspond to a final stage of 50S biogenesis that co-purify with the mature 50S. Source data are provided as a source data file.

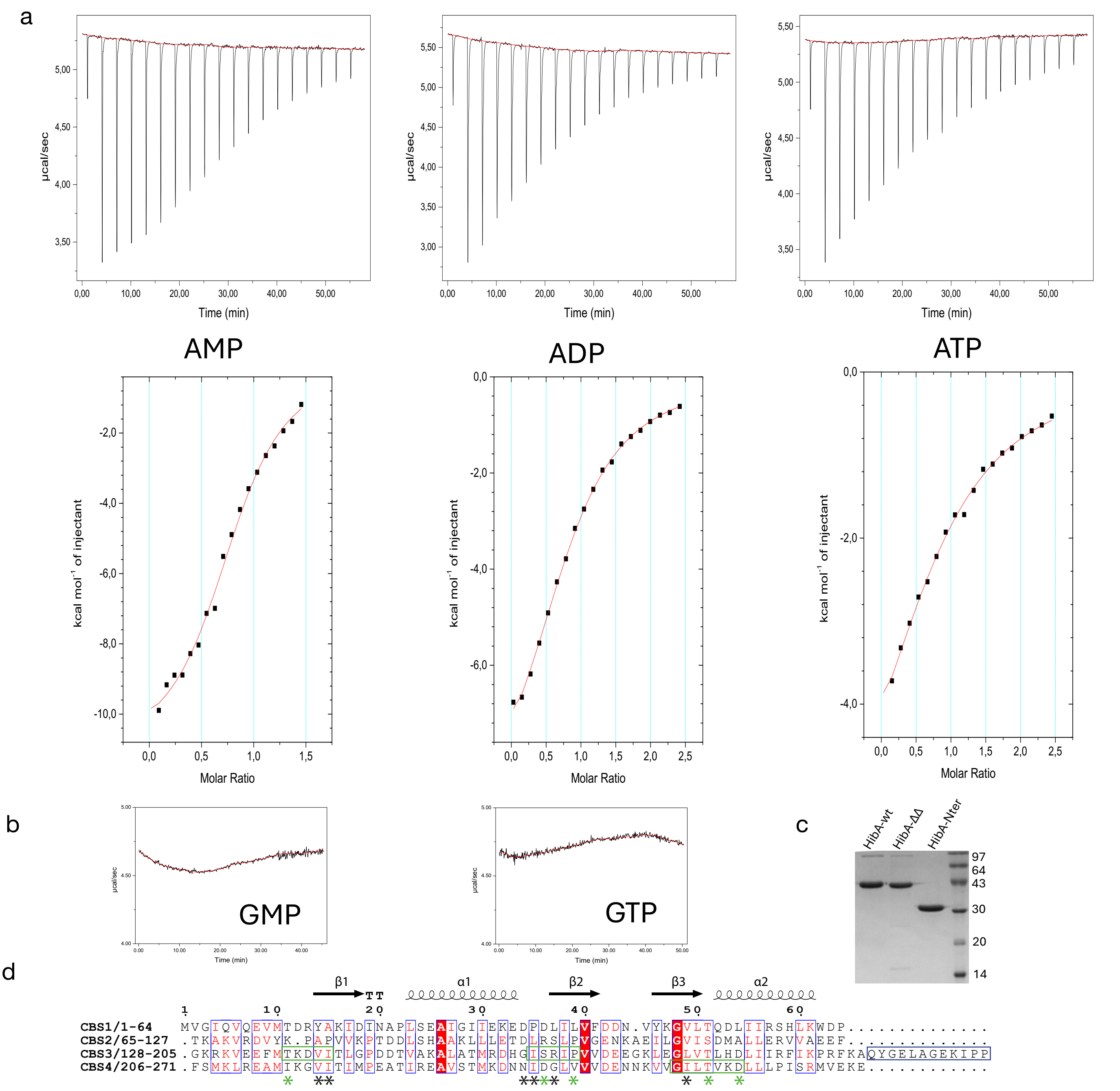

Supplementary fig. 11: Nucleotide binding to HibA

a. ITC titration curves (upper graphs) and corresponding binding isotherms (lower panels) for the interaction of HibA with AMP-Mg2+(left), ADP-Mg2+(middle) and ATP-Mg2+(right). Example experiments are shown. Derived Kd values and stoichiometries were 12 ± 2 µM (n=0.87 ± 0.05; AMP, 3 experiments), 65 ± 1 µM (n=0.82 ± 0.03; ADP, 2 experiments) and 113 ± 21 µM (n=0.81 ± 0.1; ATP, 3 experiments).
b. ITC titration curves with GMP-Mg2+(left) or GTP-Mg2+(right) did not give detectable heat signal.
c. SDS-PAGEanalysis of the HibA-Nter protein used in ITC experiments and in vitro translation assays (Figure 5a).
d. Sequence alignement of the four HibA CBS domains. Conserved residues are framed in blue. ATP binds to the interface between CBS3 and 4. The residues involved in ATP binding are boxed in green and highlighted with stars. The green stars correspond to residues present in canonical binding sites of adenosine derivatives in a Bateman module[7]. Three blocks of residues are involved in ATP binding. The conserved second motif h-y-y'-h-P (where «h »is hydrophobic and « y » any residue) favors the interaction with adenosyl groups while preventing binding of guanosyl derivatives[6,7]. Other residues, by their carbonyl groups favor the binding of adenosine derivatives as compared to guanosine derivatives. The third block of residues contains the G-h-h'-T/S-x-x'-D/N AMP binding motif. The aspartate residue of this motif is conserved and interacts with the 2' and 3' hydroxyl groups of ribose. Each CBS domain contains a potential binding cavity for nucleotides, yet a Bateman module typically binds only one adenylated ligand[6]. Here, the absence of ATP binding in the second Bateman domain is explained by the presence of bulky side chains residues that obstruct the cavity and by the absence of conserved residues known to be important for adenosine derivatives binding. Source data are provided as a source Data file.

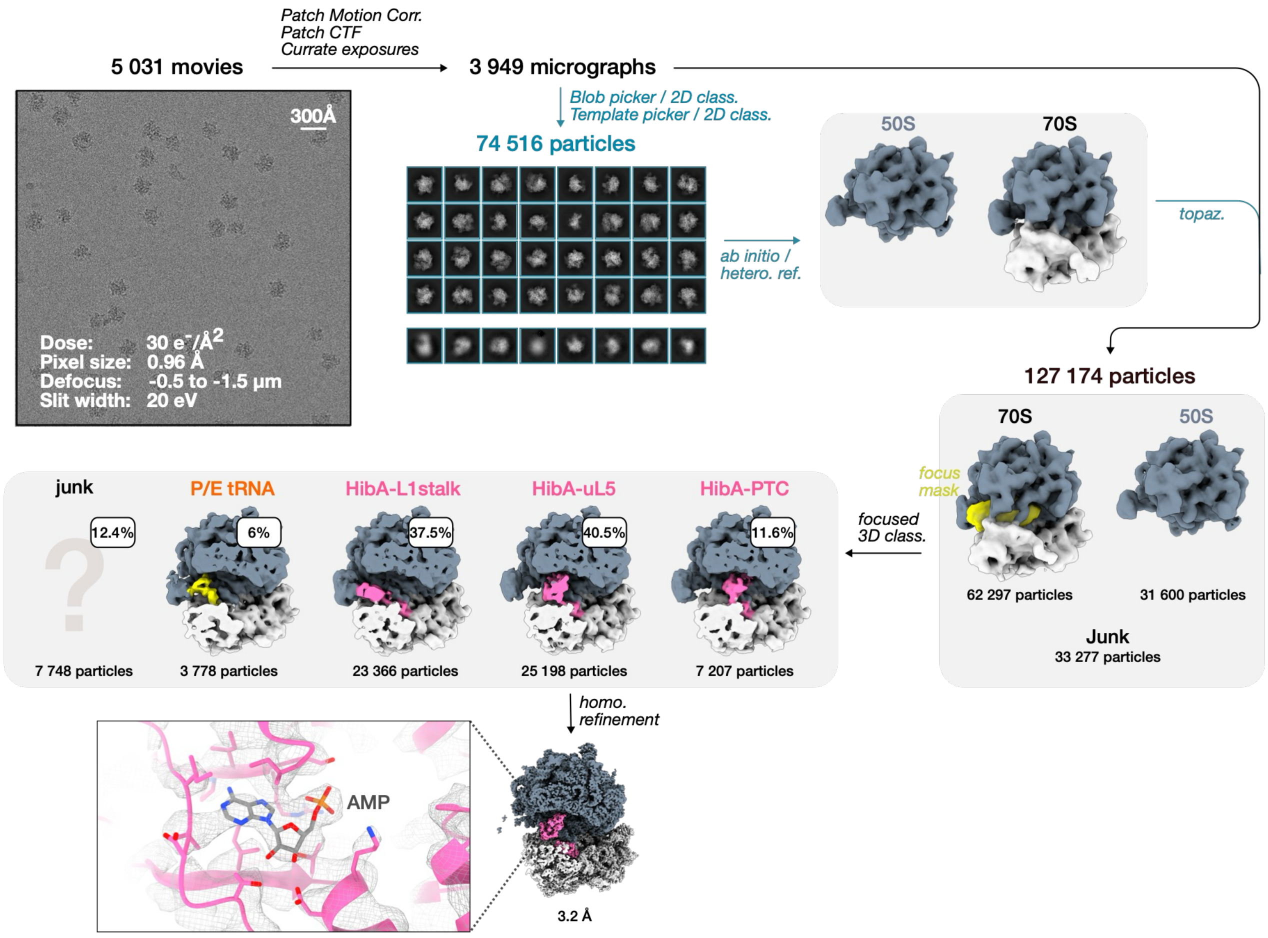

Supplementary fig. 12: Cryo-EM structure determination of *in vitro* reconstituted hibernating ribosomes in the presence of AMP.

Image processing was performed in cryoSPARC v4.1. Following motion correction and CTF estimation, micrographs were filtered based on CTF fit (<5 Å) and ice thickness. Particles were initially picked with the blob picker and classified in 2D to generate templates for subsequent particle picking. Particles obtained from template-based picking were subjected to iterative 2D classification, ab initio reconstruction, and heterogeneous refinement to remove contaminants. The resulting clean subset was then used to train Topaz for particle picking across the dataset, yielding 74,516 particles. Extracted particles (box size 480 pixels) were classified in 3D against ribosome and junk references, and the resulting 70S ribosomes were further analyzed by 3D classification focused on tRNA-binding sites. The most populated HibA-uL5 conformation was further refined to assess whether AMP was present. The AMP binding pocket is clearly visible in the density, although the overall map quality is insufficient for reliable model building.

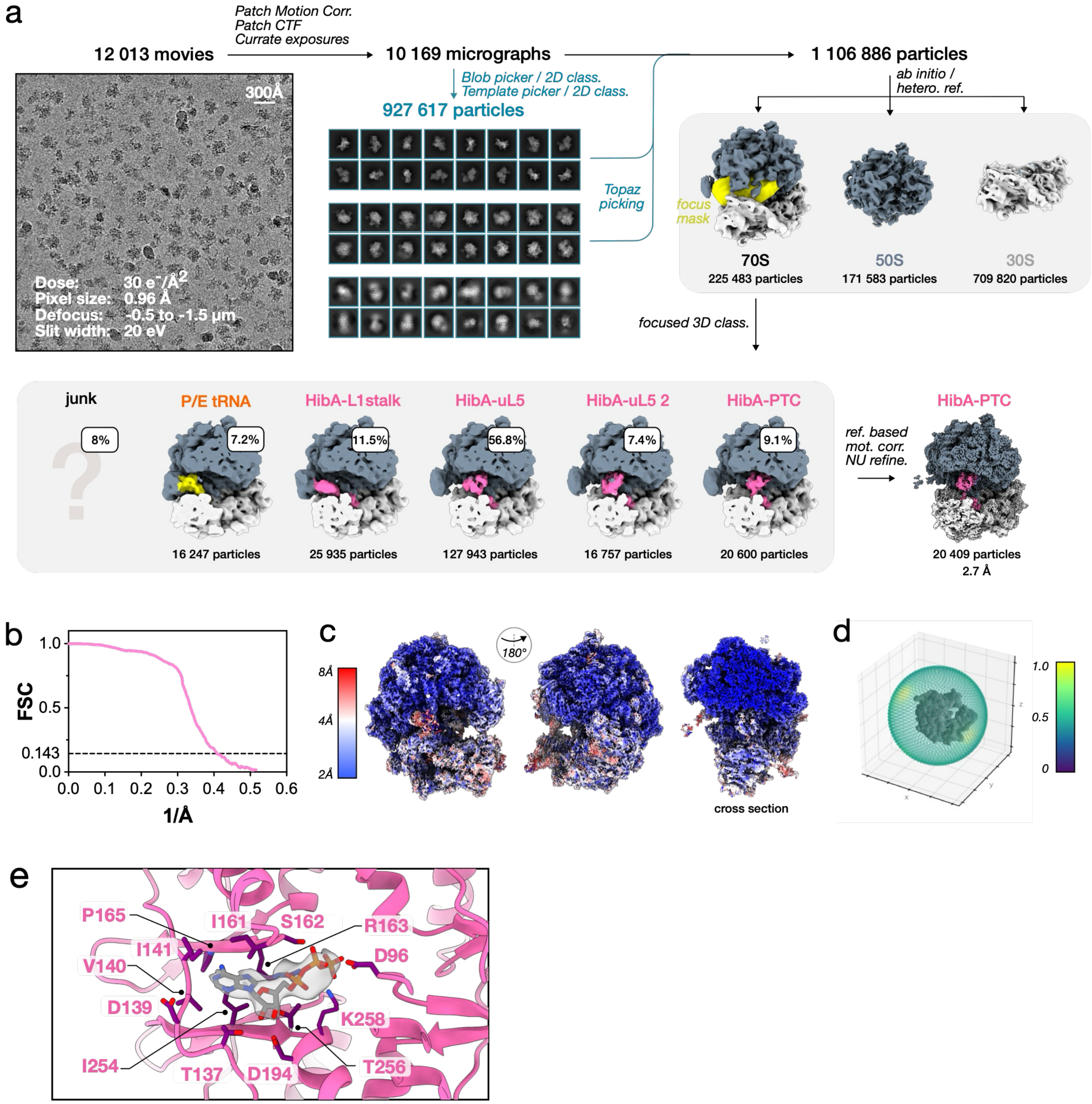

Supplementary fig. 13: Cryo-EM structure determination of *in vitro* reconstituted hibernating ribosomes in the presence of ATP.

a. Cryo-EM workflow. Data processing was performed in cryoSPARC v4.1. After motion correction and CTF estimation, exposures were filtered based on CTF fit (<5 Å) and ice thickness. Particles were initially picked with the blob picker and classified in 2D to generate templates for particle picking. Newly picked particles were classified in 2D, and ribosome particles were used to train Topaz. Particles repicked with the trained model were extracted in 420-pixel boxes and classified in 3D against ribosome and junk references. The resulting 70S ribosomes were subjected to focused classification on the tRNA-binding sites, and the HibA-PTC conformation was further refined following reference-based motion correction. **b.** Resolution of the final reconstructions was determined by gold-standard FSC at the 0.143 criterion. **c.** Local resolution of the final reconstruction. **d.** 3D scatter plot. **e.** Residues involved in ATP binding are shown along with the cryo-EM map around the nucleotide (EMDB-55134).

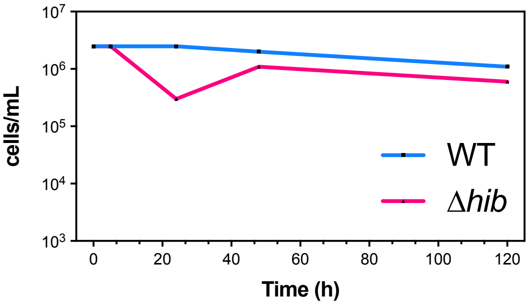

Supplementary Fig. 14: Viability after nutrient stress is unaffected by hib deletion in T. barophilus.

Most Probable Number (MPN) assays were performed to assess the recovery of T. barophilus wild type (blue) and Δhib (orange) strains after incubation in carbon-free medium at 85 °C. Cell viability was monitored at different starvation times (0 h, 5 h, 24 h, 48 h, 120 h).

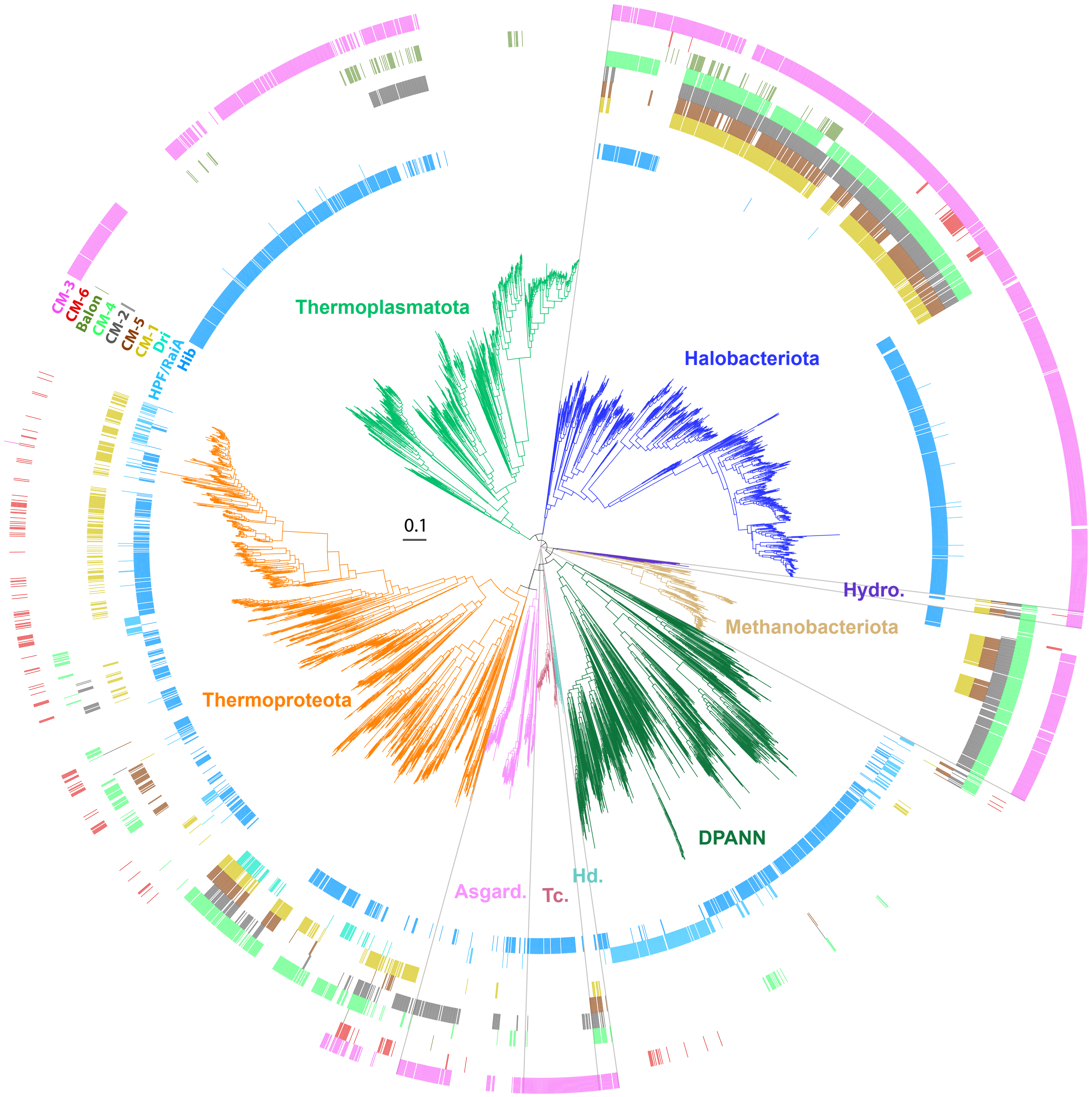

Supplementary fig. 15: Mapping of hibernation factors and homologous CM proteins on a phylogeny of 4026 archaea.

Hydro, Hydrothermarchaeota; Hd, Hadarchaeota; Tc, Thermococci; Asgard, Asgardarchaeota. Source data are provided as a Source Data file.

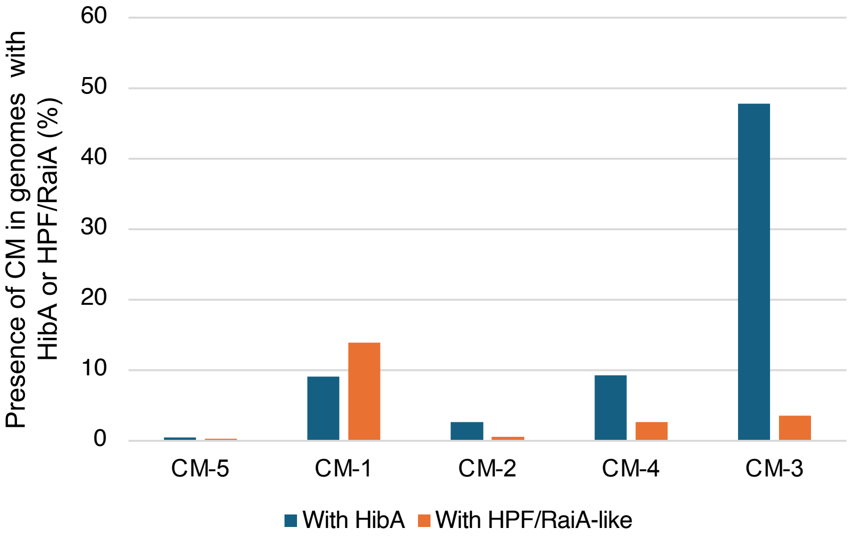

Supplementary fig. 16 : co-occurrence

Percentage of genomes coding for HibA or HPF/RaiA-likewhich also code for a standalone CBS module family (CM-5, CM-1, CM-2, CM-4, CM-3). Analysis done on a database of 4026 archaea.

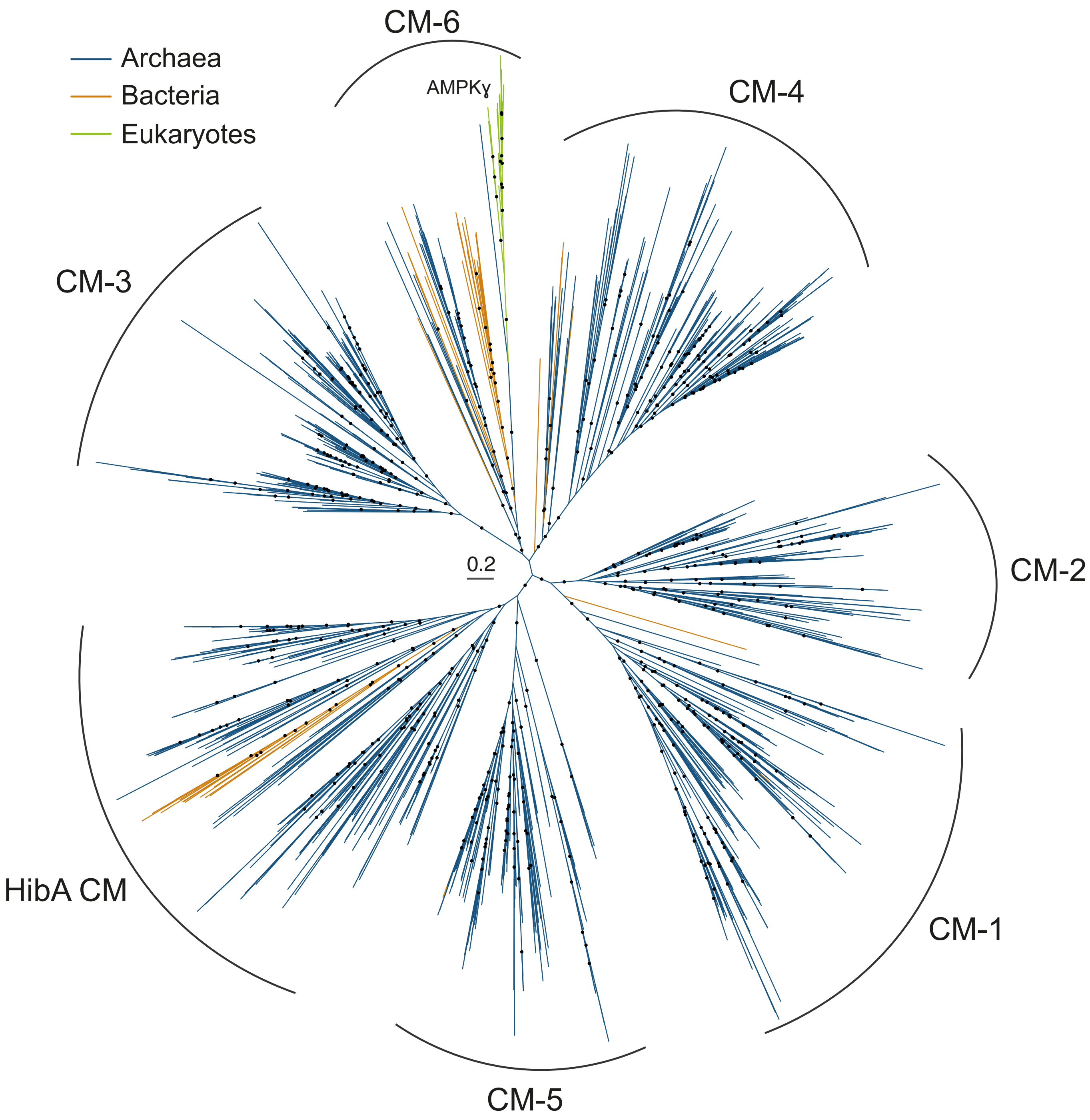

Supplementary fig. 17: Phylogeny of CM.

Tree of CBS modules (CM) including HibA CM, Dri and standalone CM from Archaea, Bacteria and Eukaryotes. AMPKg, AMP-activated protein kinase gamma subunit.

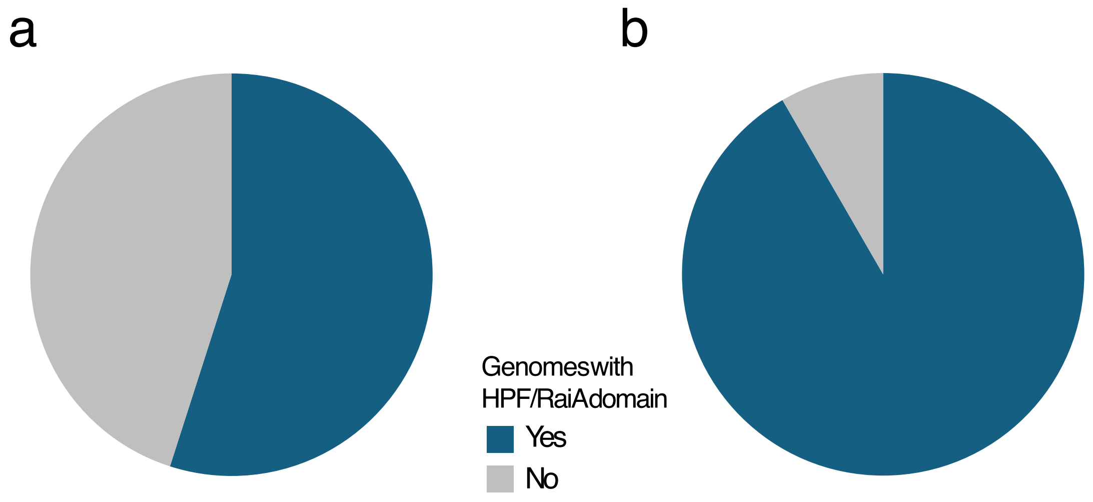

Supplementary fig. 18: Distribution and diversity of proteins with a HPF/RaiAdomain in archaea and bacteria.

Proportion of a) archaeal and b) bacterial genomes coding for these proteins.

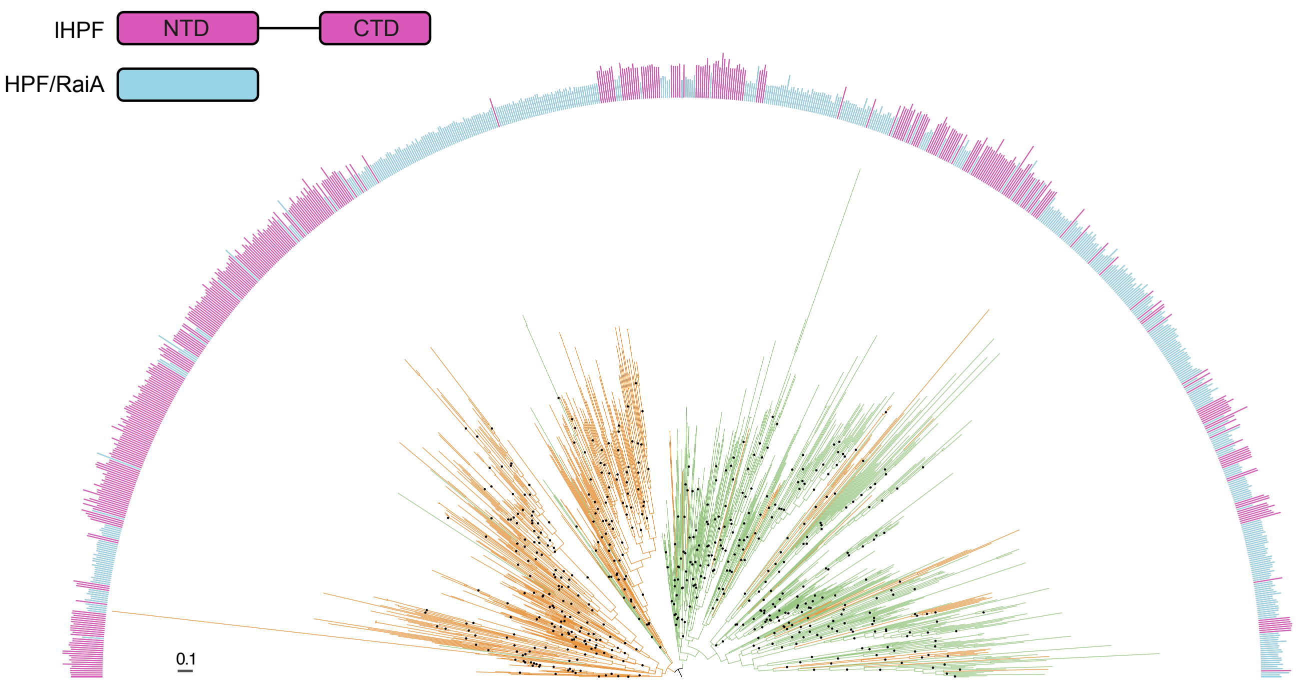

Supplementary fig. 19: Phylogeny of the HPF/RaiA and lHPF of Bacteria.

Classification between HPF/RaiA and lHPF categoies was not based on sequence length. HPF/RaiA were defined as sequences in which the CTD of lHPF (or another conserved domain) cannot be detected. Those in which this domain can be detected where defined as lHPF. Maximum-likelihood tree (LG+R7) basedon a trimmed alignment of 85 amino acid positions. Black dotes on the branches indicate ultrafast bootstrap support >90%.

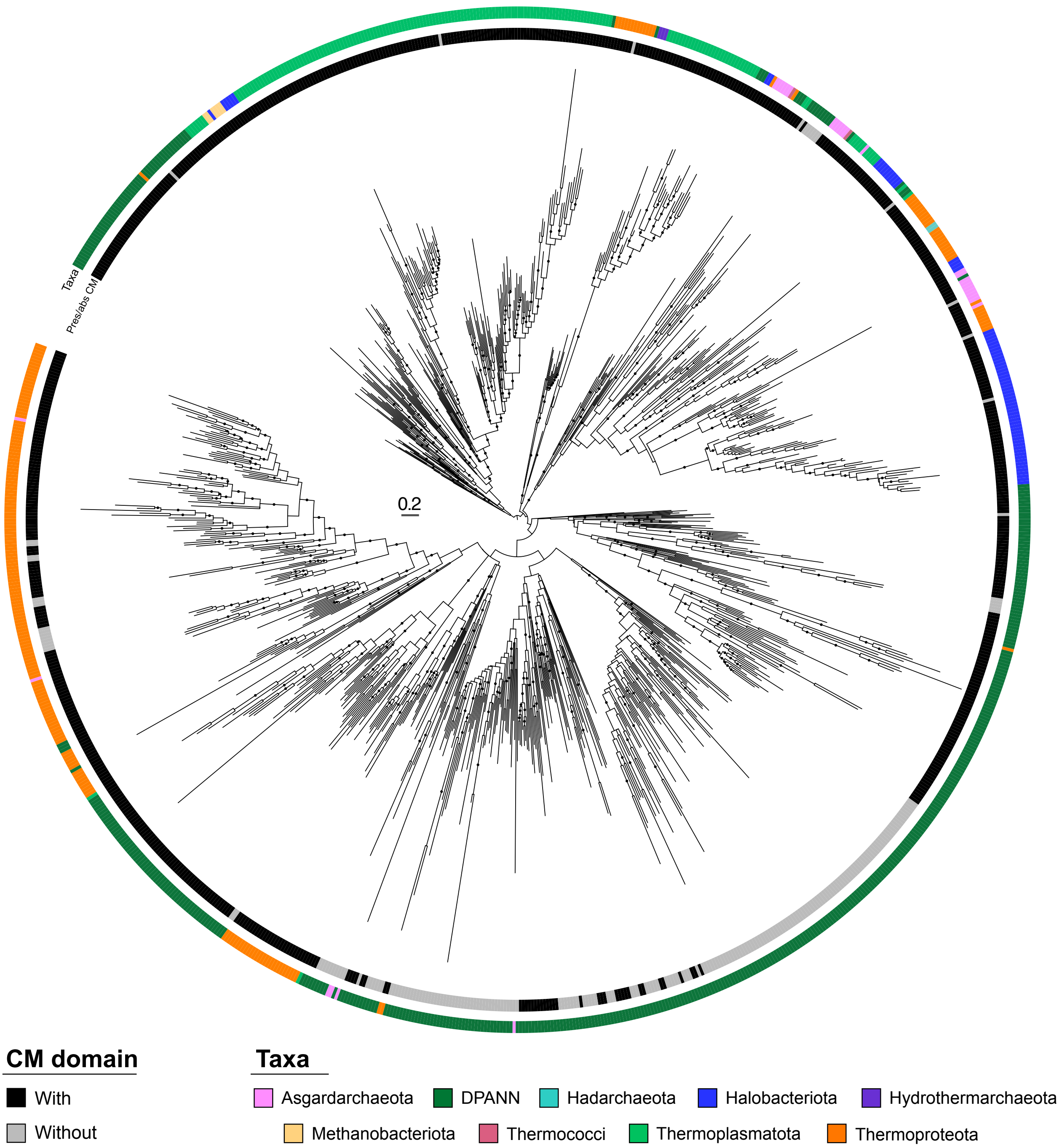

Supplementary fig. 20: Phylogeny of the HPF/RaiA domain in Archaea.

The first layer indicates the presence or absence of the CBS module (CM) in the respective proteins. The taxonomic affiliation of the sequences is indicated on the second layer. Sequences were clustered at 65% identity and a representative of each cluster was used for the phylogeny. Maximum-likelihood tree (LG+R5) based on a trimmed alignment of 97 amino acid positions. Black dotes on the branches indicate ultrafast bootstrap support >90%.

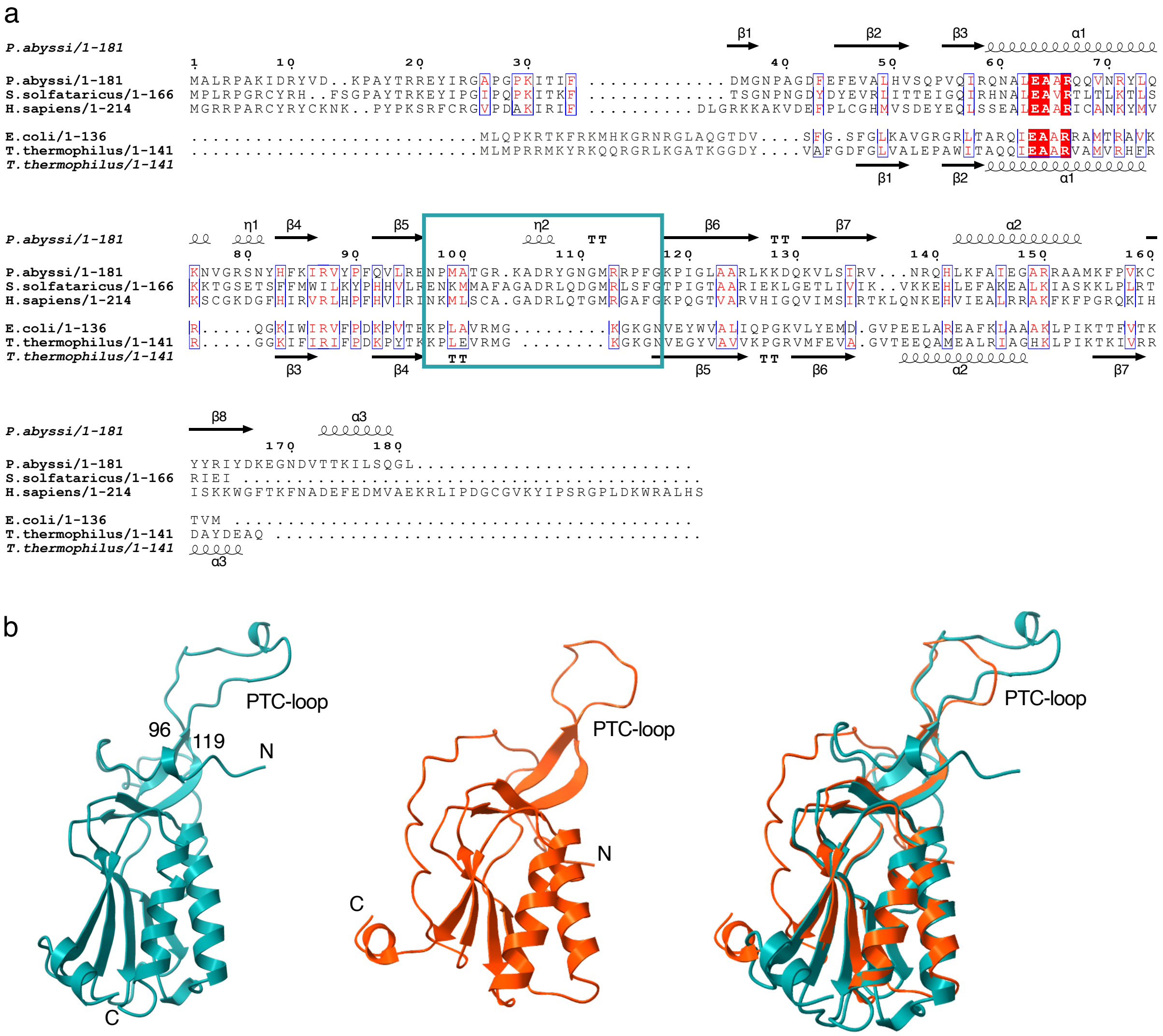

Supplementary fig. 21: uL16 in the three domains of life

a. Sequence alignment of uL16 from the three domains of life. The uL16 loop located close to the PTC is squared. This loop is longer in eukaryotes and archaea and closer to the PTC.

b. Comparison of P. abyssi uL16 (blue) with bacterial uL16 (T. thermophilus, orange, PDB4V8H).

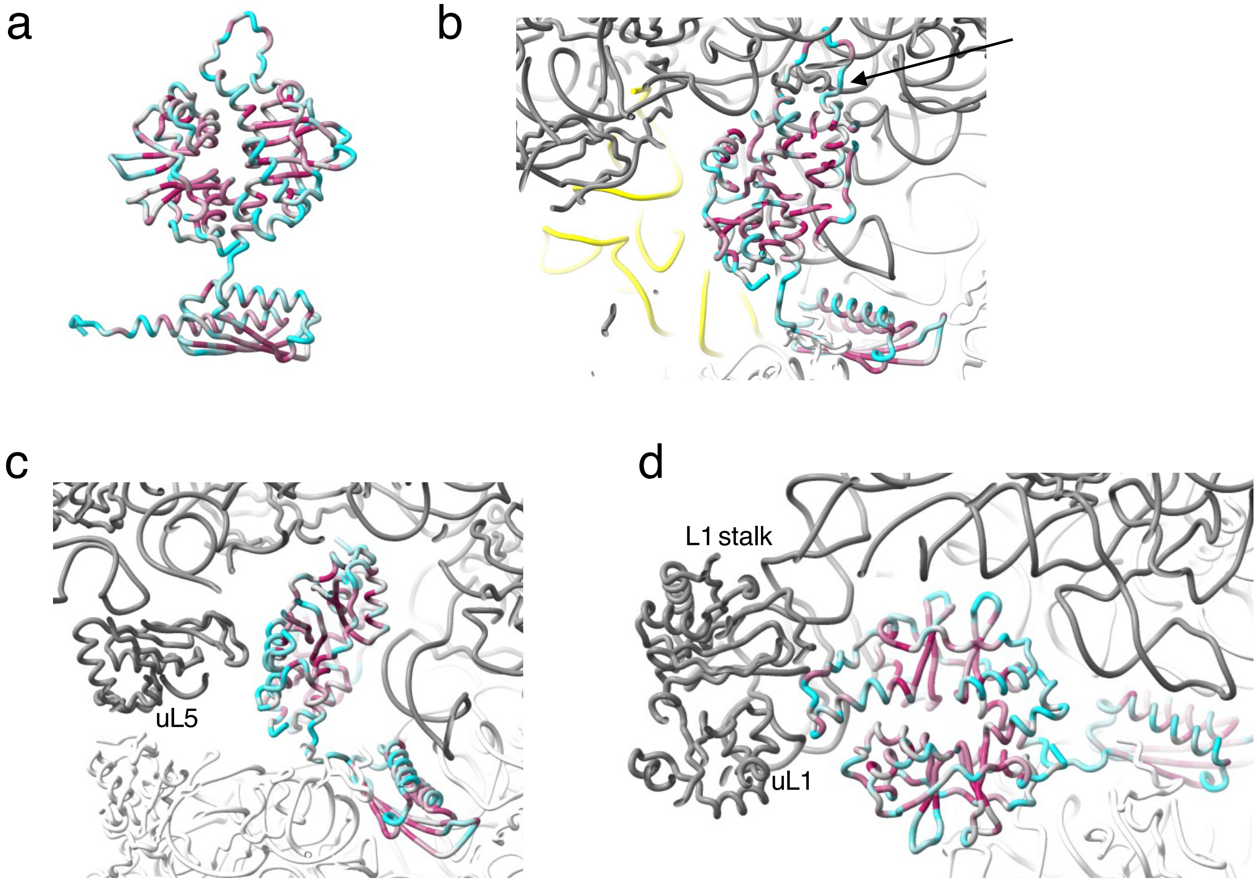

Supplementary fig.22: conservation of residues in HibA orthologs .

a. Sequence conservation has been reported on the HibA structure. Conservation score are in the 1-9 range with 1 for variable position and 9 for conserved. palette cyan-gray-maroon was used in ChimeraX (1, cyan, variable and 9, maroon, conserved).
b. HibA in the PTC conformation
c. HibA in the uL5 conformation
d. HibA in the L1 stalk conformation.
The views show that the conserved residues are located in the heart of the CBS module. Binding sites do not involve highly conserved residues.

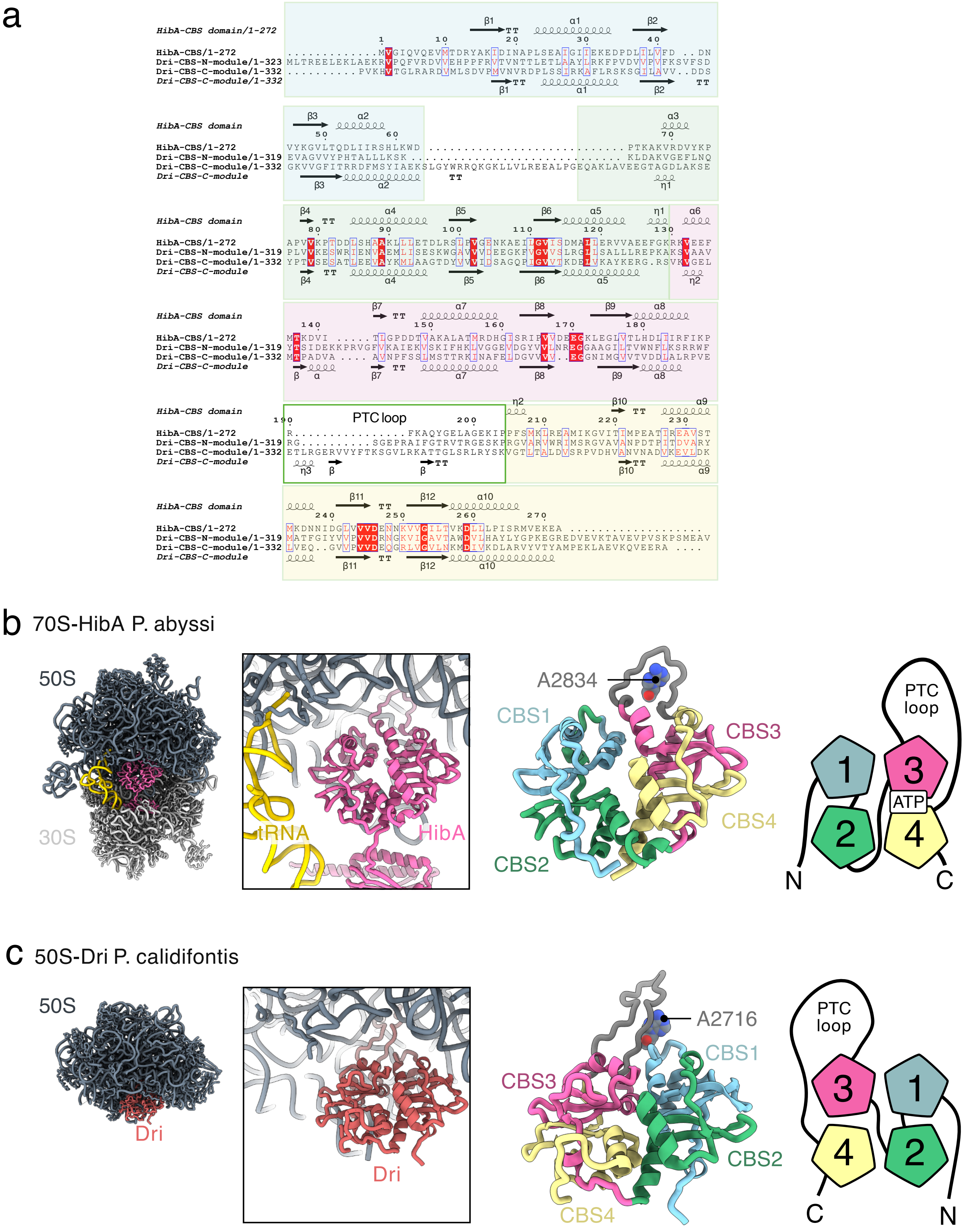

a
HibA-CBS domain/1-272
HibA-CBS/1-272
Dri-CBS-N-module/1-323
Dri-CBS-C-module/1-332
Dri-CBS-C-module/1-332
HibA-CBS domain
Dri-CBS-N-module/1-319
Dri-CBS-C-module
PTC loop
b 70S-HibA P. abyssi
50S
30S
tRNA
HibA
A2834
CBS1
CBS2
CBS3
CBS4
PTC loop
1
2
3
4
ATP
N
C
c 50S-Dri P. calidifontis
50S
Dri
Dri
A2716
CBS1
CBS2
CBS3
CBS4
PTC loop
3
1
4
2
C
N

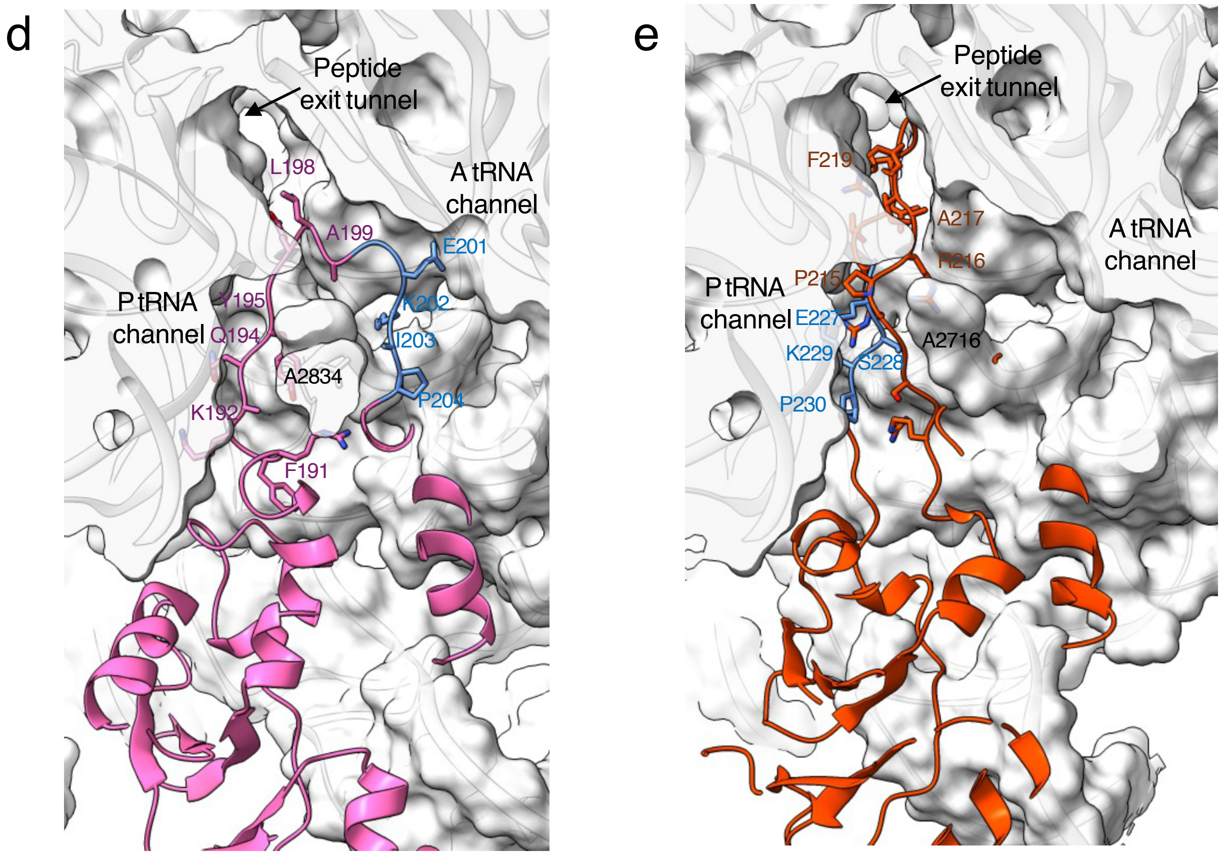

Supplementary Fig.23:Comparison of HibA and Dri CBS modules.

a. Structural alignement of the CBS modules from HibA and Dri (N and C). The CBS domains are highlighted as follows; CBS1 blue; CBS2 green, CBS3 pink, CBS4 yellow.
b. View of HibA-PTC. On the right side, a diagram shows the arrangement of the CBS domains.
c. View of Dri N-CBS module bound to the 50S (PDB 9E6Q). A diagram shows the arrangement of the CBS domains. In views b and c, the 50S subunits have been superimposed. Comparison of views b and c shows that HibA and Dri (N-module) orientations differ by 180°.
d. Interaction of the PTC loops of HibA and Dri with the ribosome: HibA loop in the PTC conformation. Residues belonging to region 190-204 and contacting the ribosome are shown in sticks and labelled. The residues colored in blue belong to the sequence $_{200}$GEKIP$_{204}$ that is conserved in Dri.
e. Interaction of the PTC loops of HibA and Dri with the ribosome: Dri N-terminal domain loop in the PTC. Residues belonging to region 210-230 and contacting the ribosome are shown in sticks and labelled. Note that the Dri loop is six residues longer as compared to HibA. The residues colored in blue belong to the sequence $_{226}$GESKP$_{230}$ that is conserved in HibA. The paths of Dri and HibA loops are different. The HibA loop surrounds the A2834 base and occupies the binding sites of the CCA of A and P site tRNAs. In contrast, the Dri loop is located entirely within the P site tRNA CCA channel. Note that the conserved sequence GEKIP/GESKP occupies different positions in the two structures.

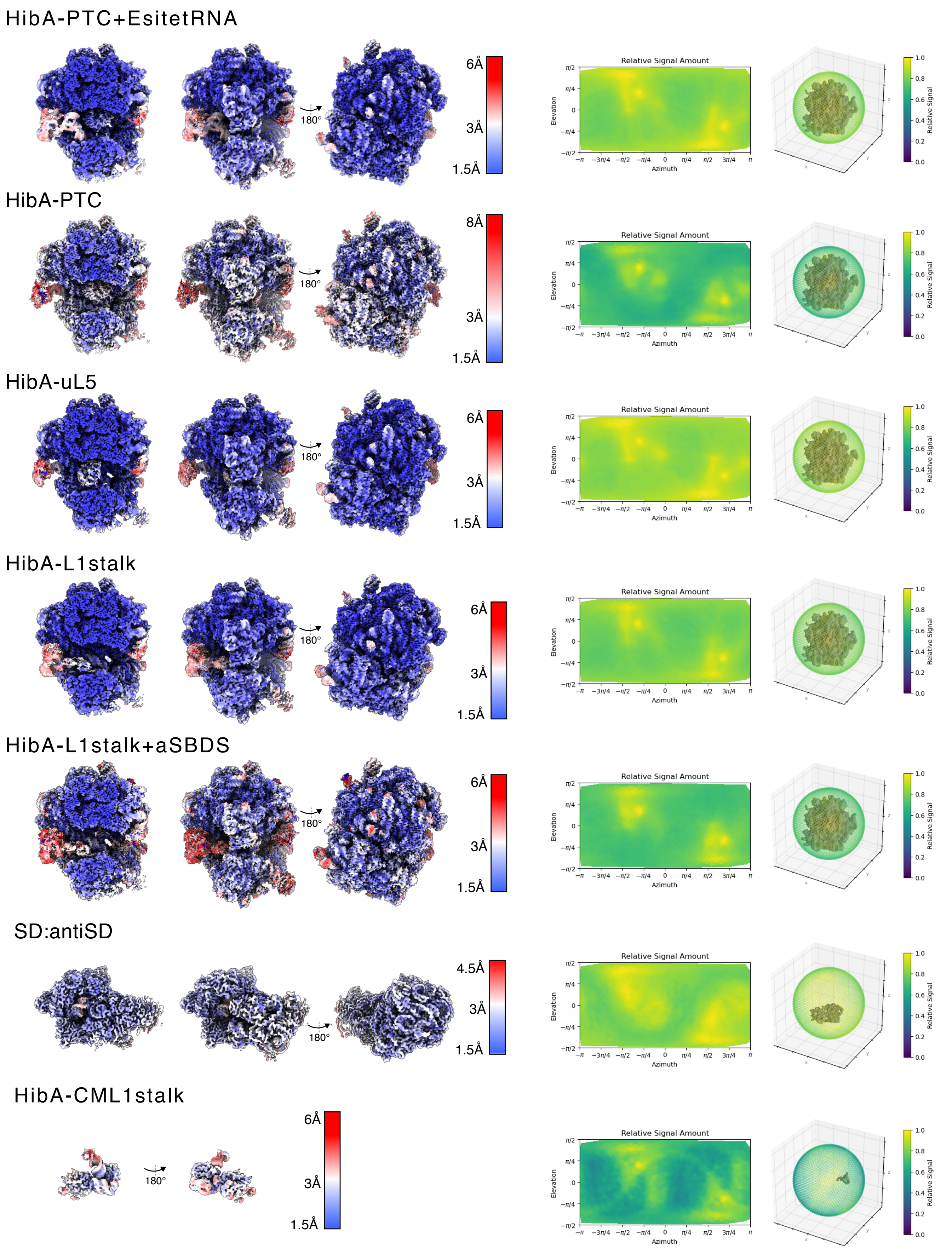

Supplementary fig. 24: Local resolution and directional distribution of cryo-EM reconstructions.

Local resolution for the different cryo-EM maps are shown on the left. Relative signal versus viewing direction, together with a 3D scatter plot are displayed on the right.

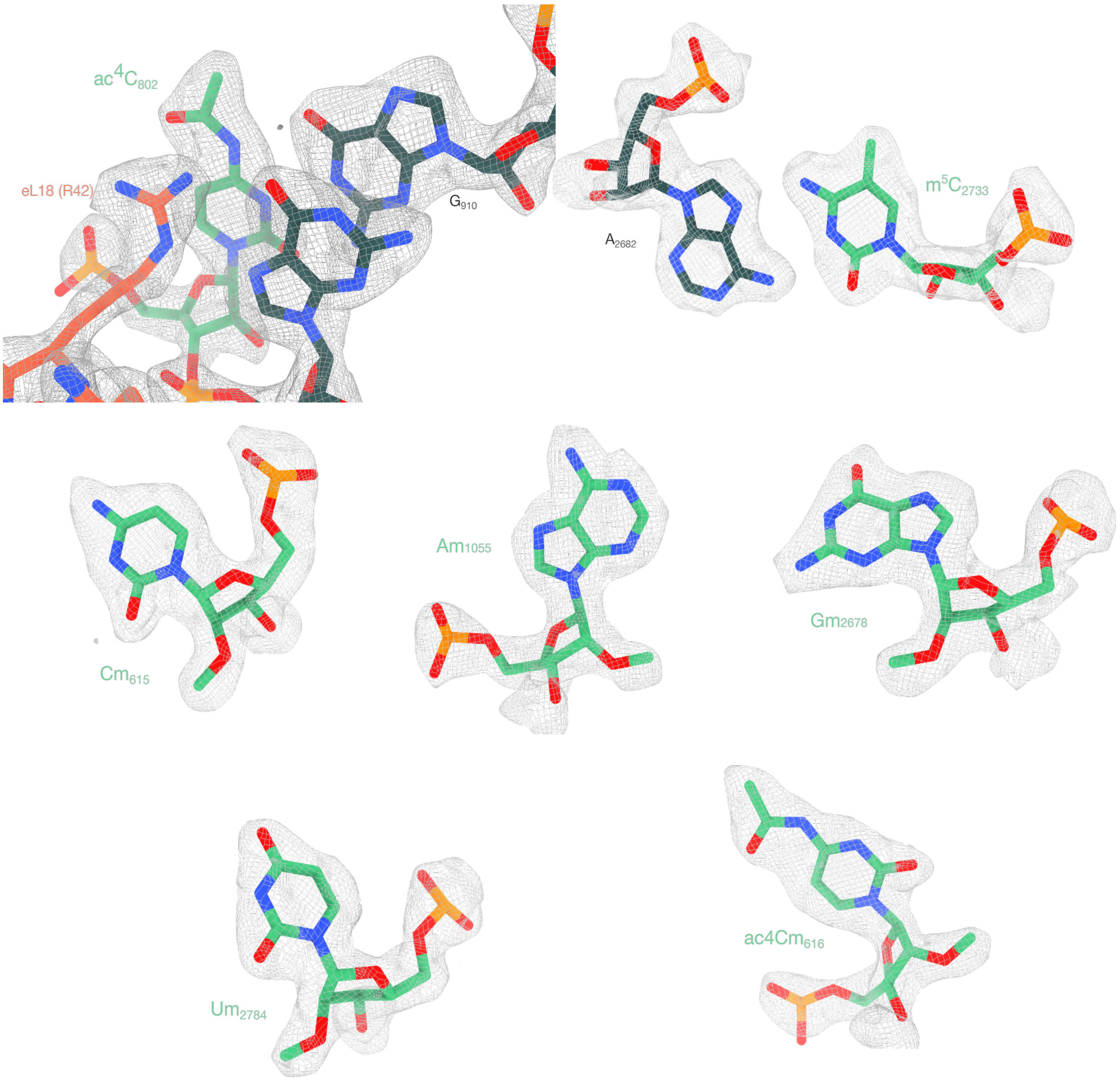

Supplementary fig. 25: Examples showing the quality of the cryo-EM map for rRNA modified nucleotides and magnesium ions

The 2.1 Å resolution cryo-EM map (PDB 9SRE, EMDB 55139) is shown using the zone command in ChimeraX.

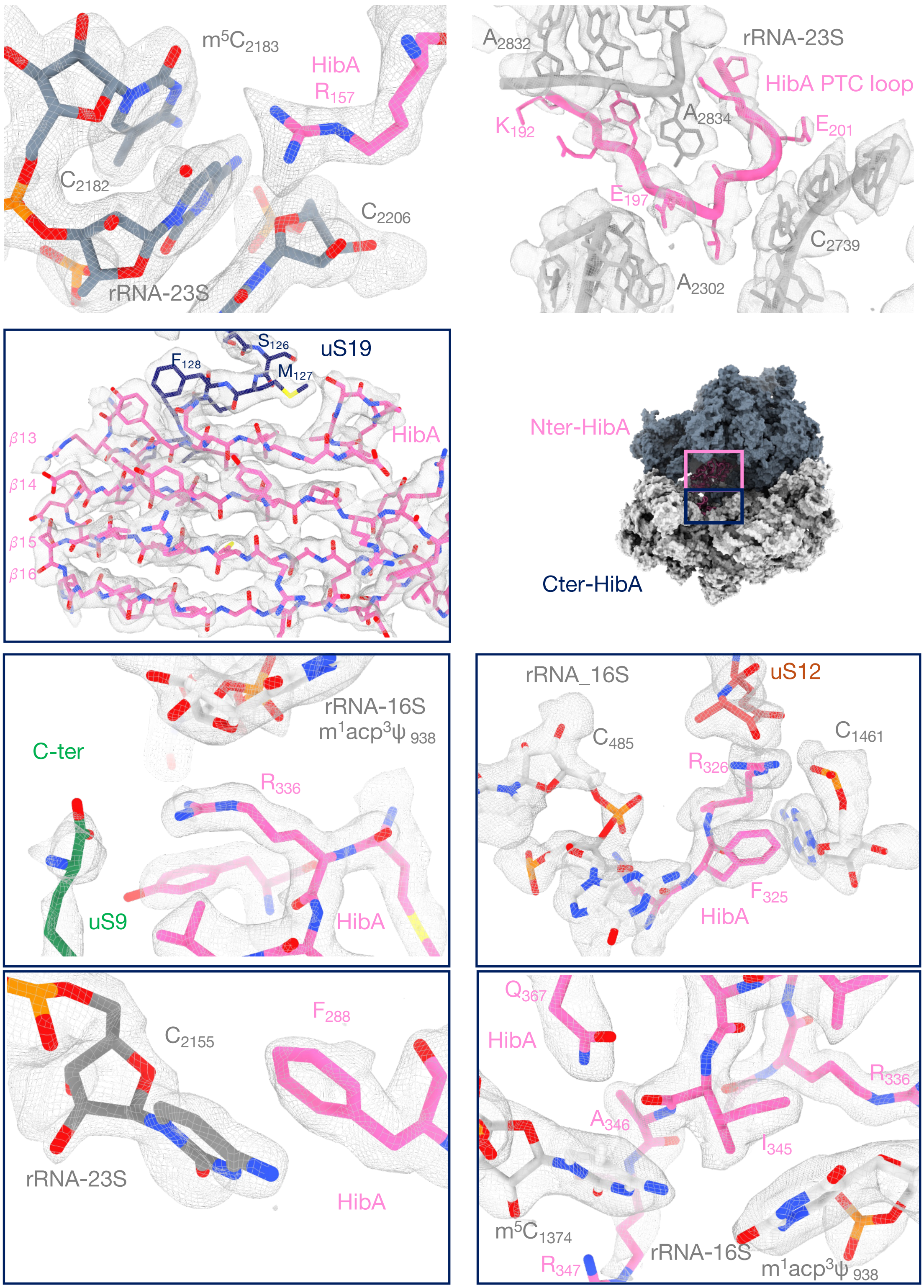

Supplementary fig. 26: Examples showing the quality of the cryo-EM map around HibA protein.

The 2.1 Å resolution cryo-EM map (PDB 9SRE, EMDB 55139) is shown using the zone command in ChimeraX.

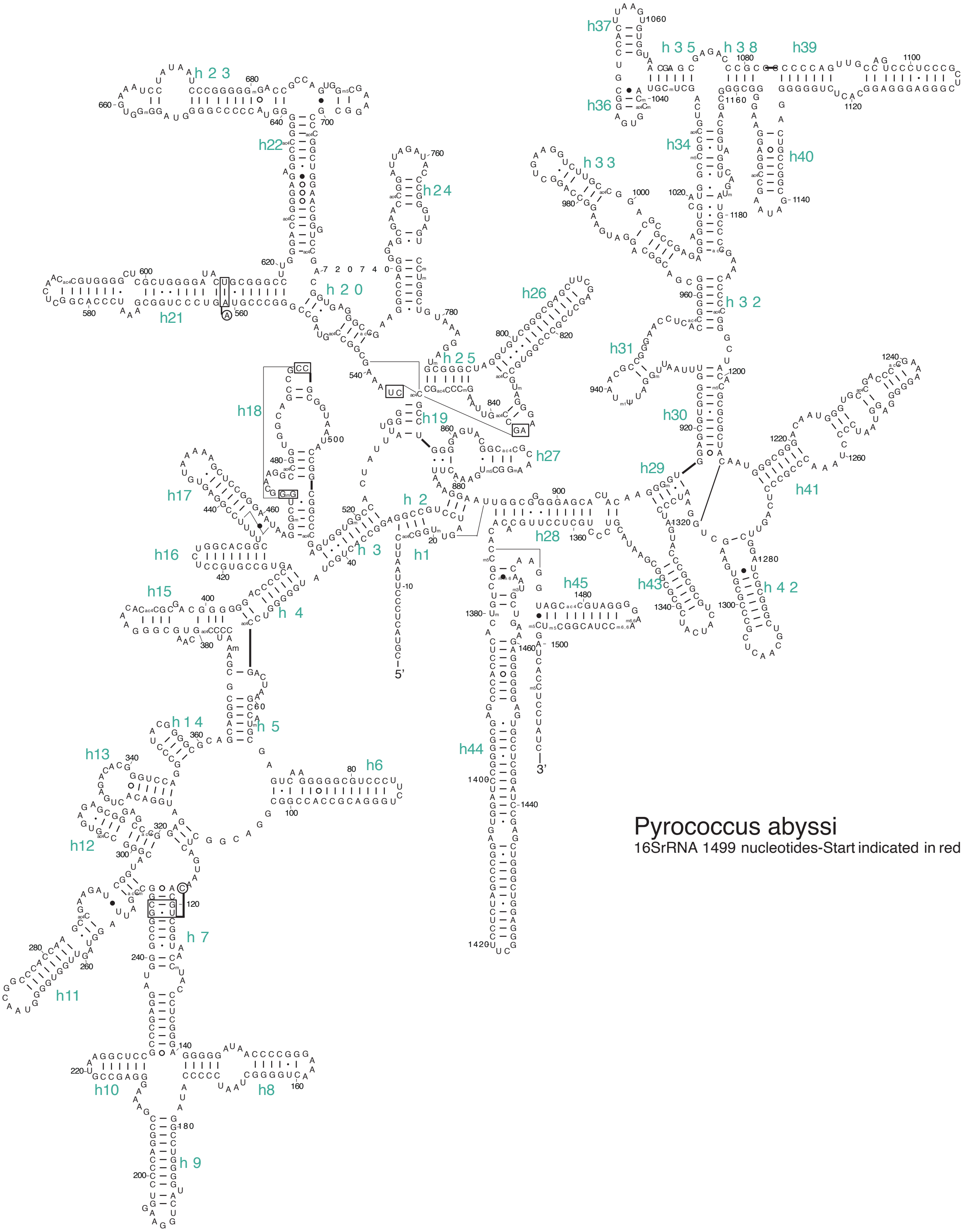

Supplementary fig. 27: 16SrRNAs from P. abyssi

Secondary structure diagrams were retrieved from https://crw-site.chemistry.gatech.edu/ and updated according to the cryo-EM structures. The 5' end of P. abyssi 16SrRNA observed in the cryo-EM structure is indicated with a red letter[8].

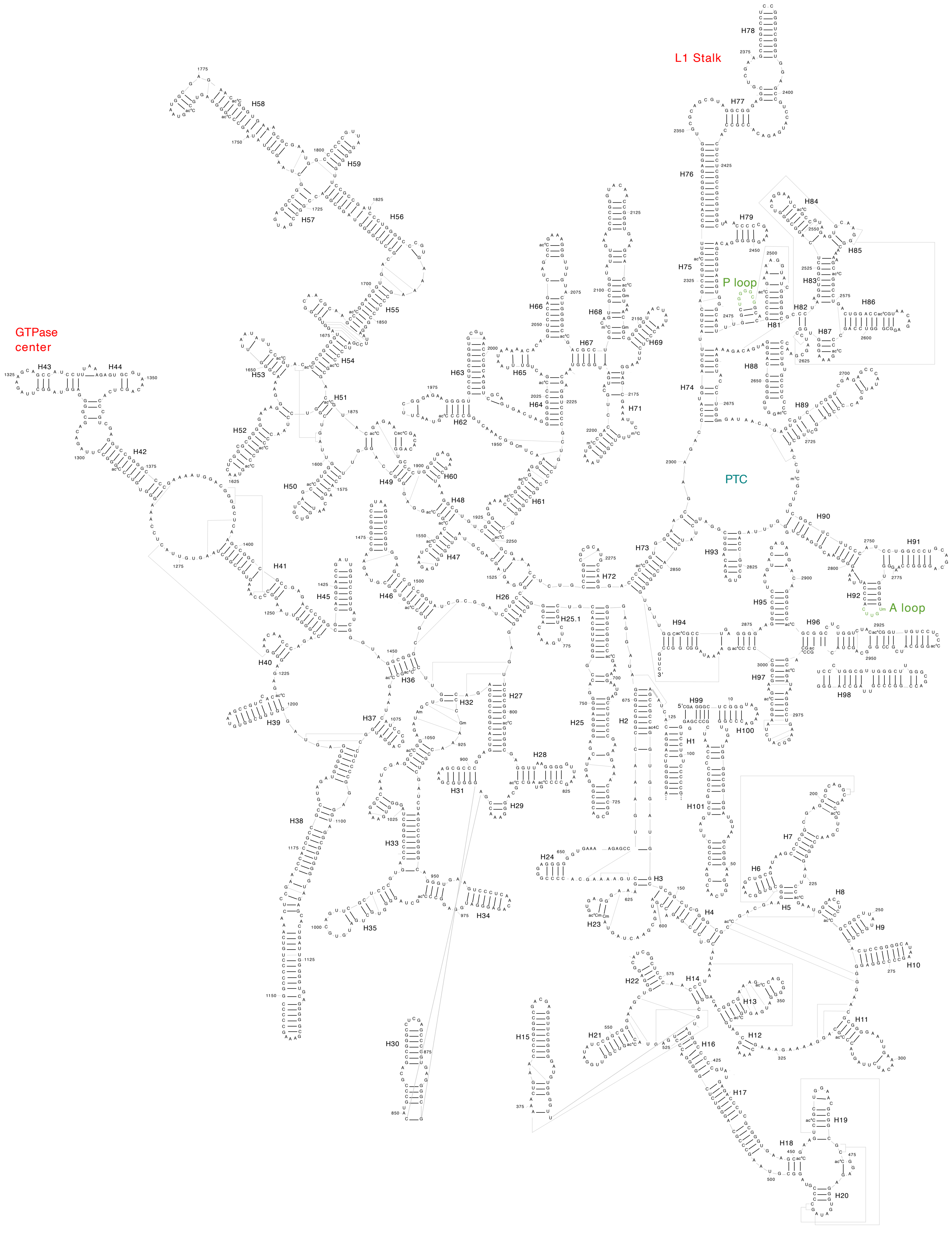

Supplementary fig. 28: 23S rRNAs from P. abyssi

Secondary structure diagrams were retrieved from https://crw-site.chemistry.gatech.edu/ and updated according to the cryo-EM structures. The 3' and 5' end of P. abyssi 23S rRNA observed in the cryo-EM structure are indicated.

Supplementary Tables:

Strains	Genotype or other relevant characteristics	Source or reference
E. coli		
DH5α	*Φ80dlacZΔm15, recA1, endA1, gyrA96, thi-1, hsdR17 (r_k−, m_k+), supE44, relA1, deoR, Δ(lacZYA-argF)U169*	Thermo Fisher Scientific, Asnières, France
T. barophilus		
UBOCC-M-3300	Δ*TERMP_00517* named WT	Birien et al. 2018[1]
RDMP94	Δ*TERMP_00517* Δ*hib* named Δ*hib*	This study
**Plasmids**		
pUPH	Pop-in Pop-out vector	Birien et al. 2018[1]
pRD507	pUPH + *hib*UpDn	This study

Supplementary Table 1: strains and plasmids.

**Primer**	**Sequences**	**Utilization**
7	GTACATTACCCTGTTATCCCTACGGCCGCTCTAGATGAACTTTGATGAACG	To analyze the loss of pUPH after pop out. Amplification of the *hmg-CoA* gene of the plasmid.
8	TGCATAGGGATAACAGGGTAATTCATCTCCCAAGCATTTTATGAGCCC	
398	TTGAGATCCTTTTTTTCTGCGCG	To analyze the loss of pUPH after pop out. Amplification of the *E. coli ori* of the plasmid.
399	CCTGACGAGCATCACAAAAATCG	
635	GCTAAGATCTCTATAAGGGTGATGACACTGTTCTTAAGG	To amplify the upstream sequence of *hibA*
636	GCCAGAGTTCAGCTTATCAAGGAATCTTTCTGTCACCTCCTTACAATTTTATCTACGC	
637	GCGTAGATAAAATTGTAAGGAGGTGACAGAAAGATTCCTTGATAAGCTTGAACTCTGGC	To amplify the downstream sequence of *hibA*
638	TCAGGTACCTTGGAATACAGATAAAAGAAGAAGTGTATGAAATAGCC	
639	ATGCAATTTATCACTAAACGAGCTTGCC	To analyze the deletion of *hibA*
640	CAATTTTATATCCCGATGGATACTCTTCTTGG	
HibA-ΔSAL-for	CAACTGGGCGGTGATGCCTCTGAATTTACCCGT	Delta-SAL
HibA-ΔSAL-back	ACGGGTAAATTCAGAGGCATCACCGCCCAGTTG	Delta-SAL
HibA-Δ2-for	CGTCGCTTTAATGAAGCAGCATACCAGGCTCGTATG	Delta KFRGVHL- substituted by AA
HibA-Δ2-bac	CATACGAGCCTGGTATGCTGCTTCATTAAAGCGACG	Delta KFRGVHL- substituted by AA
HibA-stop272_F	ATGGTTGAAAAAGAATGACGCTTCTACCTGCAA	HibA N-ter
HibA-stop272_B	TTGCAGGTAGAAGCGTCATTCTTTTTCAACCAT	HibA N-ter

Supplementary Table 2 : oligonucleotides

**Data collection**	**70S-E-tRNA-HibA-PTC**	**70S-HibA-PTC**	**70S-HibA-uL5**	**70S-HibA-L1 stalk**	**HibA-CBSmodule-L1 stalk**	**70S-HibA-L1 stalk-SBDS**	**SD-antiSD**	**HibA-ATP**
**PDB**	**9SRE**		**9SRD**	**9T7H**	**9SRF**	**9SRB 5513**	**9SRA**	**9SR9**
**EMDB**	**55139**	**9SRC 55137**	**55138**	**55636**	**55140**		**55135**	**55134**
Microscope	Titan Krios G3	Titan Krios G3	Titan Krios G3	Titan Krios G3	Titan Krios G3	Titan Krios G3	Titan Krios G3	Titan Krios G4
Camera	Gatan K3 Biocontinuum (6k x 4k)	Gatan K3 Biocontinuum (6k x 4k)	Gatan K3 Biocontinuum (6k x 4k)	Gatan K3 Biocontinuum (6k x 4k)	Gatan K3 Biocontinuum (6k x 4k)	Gatan K3 Biocontinuum (6k x 4k)	Gatan K3 Biocontinuum (6k x 4k)	TFS Falcon 4i (4k x 4k)
Magnification	105 000x	105 000x	105 000x	105 000x	105 000x	105 000x	105 000x	130 000x
Voltage (kV)	300	300	300	300	300	300	300	300
Electron exposure (e–/Å^2)	40	40	40	40	40	40	40	30
Defocus range (µm)	-0.5 to -1.5	-0.5 to -1.5	-0.5 to -1.5	-0.5 to -1.5	-0.5 to -1.5	-0.5 to -1.5	-0.5 to -1.5	-0.5 to -1.5
Pixel size (Å)	0.84	0.84	0.84	0.84	0.84	0.84	0.84	0.96
Symmetry imposed	C1	C1	C1	C1	C1	C1	C1	C1
Initial particle images (no.)	1 507 591	1 507 591	1 507 591	1 507 591	1 507 591	1 507 591	1 507 591	225 483
Final particle images (no.)	182000	24000	207000	170000	170000	45000	79361	20409
Resolution (unmasked, Å)	2.1	2.6	2.1	2.1	4.5	2.3	3.4	3.3
Resolution (masked, Å)	2.1	2.6	2.1	2.1	2.9	2.3	2.7	2.5
FSC threshold	0.143	0.143	0.143	0.143	0.143	0.143	0.143	0.143
**Refinement**								
Initial model used (PDB code)	7ZHG,4V6U, 6TH6							
Model resolution (Å)	2.1	2.5	2.1	2.1	2.9	2.3	2.7	2.5
FSC threshold	0.143	0.143	0.143	0.143	0.143	0.143	0.143	0.143
Map sharpening *B* factor (Å^2)	-40							
**Model composition**								
Non-hydrogen atoms	198252	170554	170317	172054	5729	174096	170770	3105
Protein residues	8915	8700	8671	8895	481	9131	8700	386
Nucleotides	4721	4644	4644	4644	92	4650	4654	ATP:1
Ligands	ZN:13 MG:244	ZN:13 MG:244	ZN:13 MG:244	ZN:13 MG:241		ZN:13 MG:240	ZN:13 MG:244	
Water molecules	24379	0	0	0	0	0	0	0
**Average *B* factors (Å^2)**	41.2	70.0	71.9	77.9	121.4	74.1	85.1	73.6
Protein	51.0	96.0	82.2	86.4	106.2	80.0	128.6	74.8
Nucleic acid	40.7	74.2	67.0	69.1		62.9	94.2	
Ligand	44.6							
Water molecules								
**R.m.s. deviations**								
Bond lengths (Å)	0.004	0.004	0.004	0.004	0.003	0.003	0.003	0.003
Bond angles (°)	0.670	0.584	0.574	0.567	0.595	0.636	0.605	0.487
**Validation**								
MolProbity score	1.90	1.85	1.63	1.74	2.10	2.04	1.86	1.88
Clashscore	7.46	6.91	5.30	6.72	8.60	7.91	7.60	5.55
Poor rotamers (%)	4.48	3.58	2.43	2.69	5.54	6.27	3.78	5.72
**Ramachandran plot**	98.05	97.78	97.77	97.80	97.69	98.21	98.21	98.18
Favored (%)	1.94	2.22	2.20	2.16	2.31	1.73	1.79	1.82
Allowed (%)	0.01	0	0.02	0.05	0.0	0.06	0	0
Disallowed (%)								
**Correlation CC**								
Mask CC	0.82	0.85	0.91	0.89	0.88	0.88	0.71	0.69
Volume CC	0.82	0.84	0.90	0.88	0.88	0.87	0.70	0.69

Supplementary Table 3: Cryo-EM data collection, refinement and validation statistics for ribosomal complexes isolated from cell lysate.

rRNA modification	PDB identifier	Position in P. abyssi 23S rRNA
N4-acetylcytidine	4AC	458, 474, 533, 641, 694, 802, 829, 835, 922, 981, 1048, 1065, 1068, 1222, 1293, 1460, 1550, 1554, 1557, 1594, 1617, 1621, 1662, 1667, 1695, 1755, 1765, 1780, 1867, 1873, 1885, 1934, 1938, 1962, 2001,2027, 2062, 2083, 2136, 2249, 2287, 2329, 2495, 2545, 2548, 2570, 2585, 2608, 2642, 2718, 2865, 2908, 2925, 2937, 2960, 2966, 2992, 3004
2'-O methylcytidine	OMC	615, 1948
4-acetyl-2'O-methyllcytidine	LHH	616
2'-O methylguanidine	OMG	789, 923, 2138, 2144, 2147, 2678
2'-O methyladenine	A2M	1055, 2173
5-methylcytidine	M5C	2093, 2183, 2198, 2733
2'-O methyluridine	OMU	2784

Supplementary Table 4 : **Modified residues localized in 23S rRNA sequence of *P. abyssi* ribosome.** The name of the rRNA modification and its position in 23S rRNA sequences are indicated. N4-acetylcytidine modifications systematically target the second cytosine of a 5'CCG3'[2,3]

Supplementary References

1. Birien, T. *et al.* Development of an Effective 6-Methylpurine Counterselection Marker for Genetic Manipulation in Thermococcus barophilus. *Genes* **9**, 77 (2018).
2. Coureux, P.-D., Lazennec-Schurdevin, C., Bourcier, S., Mechulam, Y. & Schmitt, E. Cryo-EM study of an archaeal 30S initiation complex gives insights into evolution of translation initiation. *Commun. Biol.* **3**, 58 (2020).
3. Sas-Chen, A. *et al.* Dynamic RNA acetylation revealed by quantitative cross-evolutionary mapping. *Nature* **583**, 638–643 (2020).
4. Schmitt, E. *et al.* Recent Advances in Archaeal Translation Initiation. *Front. Microbiol.* **11**, 584152 (2020).
5. Melnikov, S., Manakongtreecheep, K. & Söll, D. Revising the Structural Diversity of Ribosomal Proteins Across the Three Domains of Life. *Mol. Biol. Evol.* **35**, 1588–1598 (2018).

6. Baykov, A. A., Tuominen, H. K. & Lahti, R. The CBS Domain: A Protein Module with an Emerging Prominent Role in Regulation. *ACS Chem. Biol.* **6**, 1156–1163 (2011).

7. Ereño-Orbea, J., Oyenarte, I. & Martínez-Cruz, L. A. CBS domains: Ligand binding sites and conformational variability. *Arch. Biochem. Biophys.* **540**, 70–81 (2013).

8. Bourgeois, G. *et al.* Structures of Saccharolobus solfataricus initiation complexes with leaderless mRNAs highlight archaeal features and eukaryotic proximity. *Nat. Commun.* **16**, 348 (2025).
