# Supplementary material for: A family of ribosome hibernation factors widespread in Archaea
Source: Nat Commun. 2026 Apr 27;17:5751. doi: 10.1038/s41467-026-72341-8 (PMC13324472; doi:10.1038/s41467-026-72341-8)
Supplement: Supplementary file 2 — Description of Additional Supplementary Files [file 41467_2026_72341_MOESM2_ESM.pdf]

Description of Additional Supplementary Files:

Supplementary Data 1: The contacts between HibA and the ribosome were calculated in ChimeraX
