# Supplementary material for: A family of ribosome hibernation factors widespread in Archaea
Source: Nat Commun. 2026 Apr 27;17:5751. doi: 10.1038/s41467-026-72341-8 (PMC13324472; doi:10.1038/s41467-026-72341-8)
Supplement: Supplementary file 4 — Reporting Summary [file 41467_2026_72341_MOESM4_ESM.pdf]

nature portfolio

Corresponding author(s): Didier Flament, Emanuelle Schmitt & Guillaume Borrel

Last updated by author(s): 09/12/2025

Reporting Summary

Nature Portfolio wishes to improve the reproducibility of the work that we publish. This form provides structure for consistency and transparency in reporting. For further information on Nature Portfolio policies, see our Editorial Policies and the Editorial Policy Checklist.

Statistics

For all statistical analyses, confirm that the following items are present in the figure legend, table legend, main text, or Methods section.

n/a	Confirmed	
☐	☒	The exact sample size (*n*) for each experimental group/condition, given as a discrete number and unit of measurement
☐	☒	A statement on whether measurements were taken from distinct samples or whether the same sample was measured repeatedly
☒	☐	The statistical test(s) used AND whether they are one- or two-sided<br>*Only common tests should be described solely by name; describe more complex techniques in the Methods section.*
☒	☐	A description of all covariates tested
☒	☐	A description of any assumptions or corrections, such as tests of normality and adjustment for multiple comparisons
☐	☒	A full description of the statistical parameters including central tendency (e.g. means) or other basic estimates (e.g. regression coefficient) AND variation (e.g. standard deviation) or associated estimates of uncertainty (e.g. confidence intervals)
☒	☐	For null hypothesis testing, the test statistic (e.g. *F*, *t*, *r*) with confidence intervals, effect sizes, degrees of freedom and *P* value noted<br>*Give P values as exact values whenever suitable.*
☒	☐	For Bayesian analysis, information on the choice of priors and Markov chain Monte Carlo settings
☒	☐	For hierarchical and complex designs, identification of the appropriate level for tests and full reporting of outcomes
☒	☐	Estimates of effect sizes (e.g. Cohen's *d*, Pearson's *r*), indicating how they were calculated

Our web collection on statistics for biologists contains articles on many of the points above.

Software and code

Policy information about availability of computer code

Data collection	PeakTrack software (Teledyne), NCBI FTP, pfam FTP, EPU (Thermo Fisher)
Data analysis	Excel, CD-Search, HM MER v3.3.2, MAFFT v7.490, BMGE vl.12, FASTTREE v2.1.10, BLASTP v2.8.l+, ITOL, IQ-TREE v2.1.2. Cryosparc 4; Phenix (refinement); Prism (GraphPad); ImageJ; ChimeraX

For manuscripts utilizing custom algorithms or software that are central to the research but not yet described in published literature, software must be made available to editors and reviewers. We strongly encourage code deposition in a community repository (e.g. GitHub). See the Nature Portfolio guidelines for submitting code & software for further information.

Data

Policy information about availability of data

All manuscripts must include a data availability statement. This statement should provide the following information, where applicable:
- Accession codes, unique identifiers, or web links for publicly available datasets
- A description of any restrictions on data availability
- For clinical datasets or third party data, please ensure that the statement adheres to our policy

A data availability statement provides the PDB Ids for structural models, the EMDB Ids for cryo-EM maps and the ProteomeXchange dataset Id for MS data.

Research involving human participants, their data, or biological material

Policy information about studies with human participants or human data. See also policy information about sex, gender (identity/presentation), and sexual orientation and race, ethnicity and racism.

Reporting on sex and gender	N/A
Reporting on race, ethnicity, or other socially relevant groupings	N/A
Population characteristics	N/A
Recruitment	N/A
Ethics oversight	N/A

Note that full information on the approval of the study protocol must also be provided in the manuscript.

Field-specific reporting

Please select the one below that is the best fit for your research. If you are not sure, read the appropriate sections before making your selection.

- [x] Life sciences
- [ ] Behavioural & social sciences
- [ ] Ecological, evolutionary & environmental sciences

For a reference copy of the document with all sections, see nature.com/documents/nr-reporting-summary-flat.pdf

Life sciences study design

All studies must disclose on these points even when the disclosure is negative.

Sample size	No sample size calculations were conducted. Triplicate samples were measured for cell growth experiments to determine measurements of the mean and standard deviation.<br>For the genome databases, no sample size calculation was performed. We sampled the largest number of archaeal genomes as possible and then de-replicate them to remove redundant genomes of lower quality. More precisely, all archaeal genomes available in NCBI (2024/01/16) were collected, as well as genomes from other published datasets, including Earth, ocean, soil, glacier and termite MAG catalogues (Nayfach et al. 2021; Nishimura et Yoshizawa 2022; Ma et al. 2023; Liu et al. 2022; Arora et al. 2022). These genomes were dereplicated at 92% ANI using dRep (Olm et al. 2017), and only genomes with more than 80% completeness and less than 5% contamination according to CheckM were retained (Parks et al. 2015). This led to a database of 4026 archaeal genomes, used for protein distribution analyses. We also built a smaller database of 388 archaeal genomes to ease phylogenetic analysis and representations. This database is a manually curated sub-selection of genomes covering main archaeal lineages. For Bacteria we used a previously published database of 1067 genomes covering all major lineages (Witwinowski et al., 2022).<br>Extensive collection of Cryo-EM images were collected. After particle extractions and 2D/3D classification, as many particles as possible were used for each cryo-EM map. The resolution achieved, calculated with Gold standard methods, depends on the number of particles used.
Data exclusions	Cryo-EM images with gross surface contamination or bad CTF estimation metrics were discarded.
Replication	Triplicates samples were analysed for experiments and followed the same trend.
Randomization	N.A.
Blinding	N.A.

Reporting for specific materials, systems and methods

We require information from authors about some types of materials, experimental systems and methods used in many studies. Here, indicate whether each material, system or method listed is relevant to your study. If you are not sure if a list item applies to your research, read the appropriate section before selecting a response.

Materials & experimental systems

n/a	Involved in the study
☒	☐ Antibodies
☒	☐ Eukaryotic cell lines
☒	☐ Palaeontology and archaeology
☒	☐ Animals and other organisms
☒	☐ Clinical data
☒	☐ Dual use research of concern
☒	☐ Plants

Methods

n/a	Involved in the study
☒	☐ ChIP-seq
☐	☒ Flow cytometry
☒	☐ MRI-based neuroimaging

Plants

Seed stocks

Report on the source of all seed stocks or other plant material used. If applicable, state the seed stock centre and catalogue number. If plant specimens were collected from the field, describe the collection location, date and sampling procedures.

Novel plant genotypes

Describe the methods by which all novel plant genotypes were produced. This includes those generated by transgenic approaches, gene editing, chemical/radiation-based mutagenesis and hybridization. For transgenic lines, describe the transformation method, the number of independent lines analyzed and the generation upon which experiments were performed. For gene-edited lines, describe the editor used, the endogenous sequence targeted for editing, the targeting guide RNA sequence (if applicable) and how the editor was applied.

Authentication

Describe any authentication procedures for each seed stock used or novel genotype generated. Describe any experiments used to assess the effect of a mutation and, where applicable, how potential secondary effects (e.g. second site T-DNA insertions, mosiacism, off-target gene editing) were examined.

Flow Cytometry

Plots

Confirm that:

☐ The axis labels state the marker and fluorochrome used (e.g. CD4-FITC).

☐ The axis scales are clearly visible. Include numbers along axes only for bottom left plot of group (a 'group' is an analysis of identical markers).

☐ All plots are contour plots with outliers or pseudocolor plots.

☒ A numerical value for number of cells or percentage (with statistics) is provided.

Methodology

Sample preparation

200 µL of culture were fixed with glutaraldehyde (0.5 % final concentration). After 15 min at 4 °C, the samples were stored at – 80 °C until analysis. The thawed samples were then diluted from 10 to 1000-fold with autoclaved 0.2 µm filtered salted milliQ water solution (NaCl 30 g/L) and stained with the nucleic acid specific dye SYBR®Green for 15 min at room temperature in the dark. The trigger was set on green fluorescence. Samples were delivered at a rate of 5 µL/min and analyzed during at least 1 min.

Instrument

Agilent Advanteon cytometer equipped with a laser with an excitation wave-length of 488 nm was used

Software

The data were processed using the NovoExpress tool (v1.4.1)

Cell population abundance

Cells were fixed and stained with SYBR Green to label DNA. A simple gating strategy based on fluorescence intensity was applied to discriminate cellular events from background noise and debris for cell counting purposes only. Microbial cell abundance was monitored in the range 10E6 - 10E9 cells/ml and Salted milliQ water solution and non-inoculated TRM medium were used as negative controls.

Gating strategy

boundaries for the gating strategy was determined by comparison between plots for the non inculated TRM medium and for cultures of WT T. barophilus along growth kinetics. Population abundance was monitored within these boundaries.

☐ Tick this box to confirm that a figure exemplifying the gating strategy is provided in the Supplementary Information.
