# Supplementary material for: A family of ribosome hibernation factors widespread in Archaea
Source: Nat Commun. 2026 Apr 27;17:5751. doi: 10.1038/s41467-026-72341-8 (PMC13324472; doi:10.1038/s41467-026-72341-8)
Supplement: Supplementary file 5 — Transparent Peer Review file [file 41467_2026_72341_MOESM5_ESM.pdf]

nature portfolio

Peer Review File

A family of ribosome hibernation factors widespread in Archaea

Corresponding Author: Dr Didier Flament

This file contains all reviewer reports in order by version, followed by all author rebuttals in order by version.

Version 0:

Reviewer comments:

Reviewer #1

(Remarks to the Author)
This manuscript reports the identification of a novel archaeal ribosome hibernation factor, Hib, which structurally fuses an HPF/RaiA-like domain and CBS modules as in Dri. Through structural analysis, the authors describe the binding sites and putative functions of both the HPF/RaiA and CBS domains on the ribosome. Bioinformatic analyses further reveal the distribution and evolutionary relationships of hibernation factors across archaeal species.However, whether Hib truly represents "a new family" of hibernation factors should be carefully reconsidered. Structurally, the HPF/RaiA domain appears to play the dominant role, suggesting that Hib may instead constitute a subfamily of HPF/RaiA-like factors rather than a distinct family.Overall, the work represents a conceptual advance in the study of ribosome hibernation mechanisms, but several mechanistic claims require additional evidence and clarification.
Major comments
1.Origin and naming of "Hib". The manuscript should explicitly state the origin of the name "Hib." If it derives from "hibernation," the term may be too informal, given that hibernation factors comprise diverse, structurally unrelated proteins. I strongly recommend reconsidering or justifying the choice of this name.
2.The authors emphasize two specific insertions that interact with uS19 and uS12, suggesting they are important for Hib–ribosome binding. These interactions should be experimentally validated, e.g., by deleting the two insertions separately and testing whether Hib's ribosome-binding ability is affected.
3.Line 195 describes interactions between β13 of Hib and the C-terminal tail of uS19 "extending the Hib β-sheet." What is the reference for "extending"? In Fig. 3e–f, the β-strand of uS19 is not clearly visible—please mark both β13 of Hib and the β-strand of uS19.Similarly, line 410 states that "the first insertion also interacts with the C-terminal of uS19," yet this is not labeled in Fig. 3d or Fig. S4. Please indicate the insertion clearly. The depiction of protein loops is too thick, making it difficult to discern secondary structures; consider refining these illustrations.
4.Hib's N-terminal domain shows higher affinity for AMP than ATP, yet only ATP binding is observed in the structure. Does this imply that ribosome-bound Hib cannot bind AMP? If so, this could provide mechanistic insight into the regulation of Hib–ribosome interaction. Please discuss or experimentally address this discrepancy.
5.Both Hib and Dri contain CBS modules, but their ribosome-binding modes differ. It seems that the PTC loop is critical for 50S interaction. The authors should compare the CBS–50S interfaces between Hib and Dri, identify conserved residues, and discuss shared structural principles. Moreover, since CM-1, CM-2, CM-4, and CM-5 are proposed as potential hibernation factors, while CM-3 and CM-6 are not, the authors should analyze the conservation of these CM families relative to Hib and Dri to infer universal features of CBS modules as hibernation factors. It would also be important to test whether the N-terminal domain of Hib alone can mediate ribosome hibernation. Because Hib fuses CBS and HPF/RaiA domains, what is the functional advantage of this fusion? Truncation of the N- or C-terminal domains followed by ribosome-binding and cellular assays is recommended.
6.Is the "Hib-intermediate" state merely a transitional form between Hib-L1stalk and Hib-PTC? Given its relatively high proportion (51% in Fig. 1b), this seems unlikely. If it represents a distinct state, it should be named accordingly. Since the main conclusion—"Hib acts as a hibernation factor blocking all states of the dynamic translation process"—is based on the observation of three conformations, please discuss whether these states may instead result from weak N-domain–50S interactions. Moreover, what is the biological significance of blocking "all states" of translation?
7.While the study identifies potential interactions between Hib and several factors, the discussion of how these interactions mechanistically regulate ribosome hibernation is relatively limited. Expanding this section would strengthen the understanding of Hib-mediated regulation.

Minor comments
1.Fig. 1d: What “Δ517” refers to? It corresponds to ΔTERMP_00517. Either use “WT” in the legends or keep “Δ517” consistently in the figure, with a clear explanation.
2.Ensure consistent use of “L1 stalk” (avoid mixing “L1 stalk” and “L1-stalk”). Similarly, standardize capitalization for “Hib” vs “HIB” and ribosomal protein names such as “uL1” (avoid “ul1”).
3.When describing molecular interactions, include corresponding EM density maps to substantiate the claims.
4.Fig. 3f: Label β13 directly in the figure to improve clarity.
5.Extended Data Fig. 4: It would improve clarity if the meaning of different colors were indicated in the figure.
6.Please ensure all figure titles and legends accurately describe their figures. For instance, Fig. 1a contains an extra “)”, and similar errors appear elsewhere. Some legends are grammatically incorrect or missing punctuation (e.g., Fig. 5c: “Ribosomal proteinsThe N-terminal domain…”). Titles such as “Fig. 3: Hib-PTC” are overly simplistic and could be more descriptive.
7.Avoid “data not shown.” Such expressions are discouraged in high-impact journals; either provide the data in supplementary materials or remove the statement.
8.Extended Data Fig. 9a: Keep “Nmodule” as “N-module”, “CBSdomain” as “CBS domain”. Use either “Active loop” or “PTC loop”, it is not necessary to use two names in the figure. Change “Comparison of views b. and c.” to “xxxx b and c”.
9.Fig. 6a: Change the title from “Distribution of Hib, Hib-Cter, …” to “Distribution of Hib, HPF/RaiA, …”.

Reviewer #2

(Remarks to the Author)
In the present manuscript, Clement Madru and colleagues make an outstanding discovery of a novel ribosome hibernation factor, Hib. They combine structural, genetic and biochemical characterization of Hib to demonstrate that this protein initiates ribosome hibernation and improves environmental stress tolerance in the archaeon Pyrococcus abyssi. They then provide a comprehensive evolutionary analysis to illustrate that Hib likely acts as a hibernation factor not only in their model organism but in nearly a half of studied archaeal species.

The importance of this finding is difficult to overstate. As you certainly know, ribosomes are the most metabolically expensive molecules in living cells: they account for nearly a third of a cell's dry mass and require more cellular energy for their production (60%) than all other cellular components combined! Therefore, it is not surprising that all studied species utilize a sophisticated mechanism—ribosome hibernation—for safely storing their ribosomes during starvation and stress. When cells lack hibernation factors, they can no longer recover from prolonged periods of stress.

Currently, the archaeal domain remains one of the least studied branches of life with regard to ribosome hibernation—so that its mechanisms in many archaeal species remain completely unknown. This study fills critical gaps in our knowledge by revealing several conceptually novel properties of hibernation factors, both in Archaea specifically and for the field in general.

Below, I provide my suggestions for how to improve this study, specifically its presentation. I want to stress, however, that—given the novelty and impressive amount of work,—I believe that this paper can be accepted and published as is. My main suggestions are related to how the data are presented to give the authors better words to explain the importance of this outstanding work to a broader readership.

ABSTRACT
Their abstract would certainly benefit from mentioning some of their conceptually novel findings that set this work apart from every other study of ribosome hibernation.

1. Firstly, they show that the archaeal cells of Pyrococcus abyssi initiate two parallel ribosome hibernation pathways, mediated by the previously uncharacterized proteins Hib and SBDS. While they do not have high resolution structures or genetic studies supporting the role of SBDS protein in ribosome hibernation, their Figure 2 shows that this project is a major binder of idle ribosomes in stressed archaea. They could certainly mention this fact.

2. Secondly, they understate that their structures demonstrate how a single ribosome hibernation factor, Hib, may adopt several distinct conformation in the ribosome. This finding illustrates that our somewhat naïve expectation of “single complex = single structure” may lead to failure to understand how ribosome hibernation factors work and how they can be discovered using cryo-EM.

3. Thirdly, the authors demonstrate that many archaeal species possess not a single but multiple hibernation factors simultaneously. This indicates that the identification of a single factor in a given species does not reveal the full repertoire of players involved in this essential process in a given species.

TEXT AND FIGURES
I would rewrite this story completely to simplify the message and better explain the importance of these findings:

1. Ribosome hibernation in the archaeon Pyrococcus abyssi relies on two uncharacterized factors, Hib and SBDS.
First, I would present the data from the original Figure 2 as the new Figure 1 to immediately establish the paper's main conclusion. This figure would be modified, specifically panel C, to categorize the ribosomes as "hibernating-like" and "translating-like" rather than "Hib-bound" and "Hib-free," as this more accurately describes their functional states.

Here, I want to stress that their present description of SBDS confuses readers a little bit: this protein visually resemble EF-G/eEF-1A and it is only vaguely mentioned in the results—despite being bound to essentially a third of ribosomes in stress Pyrococcus abyssi. The authors could move, for instance, the discussion of this protein from their Discussion section to the first section of the Results.

2. Hib adopts multiple distinct conformations on the ribosome.
I would then present the ribosome/Hib structures (original Figure 1) to illustrate that Hib undergoes a conformational plasticity that is unique among known hibernation factors. This finding has critical implications for how cryo-EM data of hibernating ribosomes should be processed and for the discovery of novel hibernation factors that may exhibit similar heterogeneity.

3. Hib stabilizes ribosomal subunit interactions and enhances cellular stress tolerance.
Next, I would present on subunit association and cell viability (e.g., original Figure 1 d-e) to demonstrate that Hib stabilizes ribosomes in the 70S form—a state associated with higher stability of ribosomes in non-dividing cells [PMID: 34253058, PMID: 32123037]—and thereby bolsters the archaeal cells' tolerance to stress.

On a minor note, could the authors clarify their figure caption (Fig. 1d,e) where they state that the blue line corresponds to the Delta517 strain? Is Delta517 the same as the wild type? Also, I slightly disagree with their interpretation of the growth recovery experiments, where they state that the difference between the two strains reflects the differences in their growth rate, not mortality. In fact, their Supplementary Fig. 3 does show that Hib deletion appears to decrease the viability of cells after 20-120 h of stress.

4. Hib is a highly conserved archaeal protein.
I would then present their data on Hib occurrence in Archaea to show that this protein is present in nearly a half of studied archaeal species.

5. Archaeal species appear to possess a highly redundant machinery of ribosome hibernation.
And I would finish the manuscript by explicitly stating another conceptually novel finding of their work: that many species that contain Hib have been recently characterized to bear other hibernation factors, such as Dri, aRDF and mRDF component (see, for instance, this paper: PMID: 40914243)

6. I would move all other figures—including Figures 3, 4a-b, Figure 5—to the supplementary information, as they are highly technical and do not substantially advance understanding of Hib's biology. This is because details such as hydrogen bonds and salt bridges are self-evident from the structures and are likely of interest only to a specialized audience.

I would like to stress, however, that this feedback is intended solely to improve the manuscript's presentation; it does not diminish the outstanding contribution of this study to our understanding of how living cells remain viable under stress through dormancy.

Sincerely,
Sergey Melnikov

Reviewer #3

(Remarks to the Author)
This manuscript by Madru et al. deals with the identification of Hib, a family of hibernation factors in Archaea. The authors show that Hib is an important hibernation factor in Archaea using genetic, structural and biochemical studies and computational analysis. Hib inactivates ribosomes by blocking mRNA channel and all three tRNA binding (A, P and E) sites. Deletion of hib led to decrease in 70S ribosome pools and delay in recovery from stationary phase.
The authors have obtained high-resolution cryo-EM structures of complexes of Hib with archaeal ribosomes and resolved three conformations of Hib blocking A, P and E sites. They propose that Hib acts as a hibernation factor by blocking all states of the dynamic translation process and preserve the ribosome from dissociation and degradation. In Hib:ribosome complexes extracted from archaeal cells E-site tRNA was observed, which may serve as an aa-tRNA deprivation sensor for

Hib hibernation factor. Interestingly, hibernating ribosomes also contain Shine-Dalgarno (SD):antiSD duplex although, the origin and significance of this Shine-Dalgarno (SD):antiSD duplex is not clear. Overall, this study identifies a new family of hibernation factors in Archaea and demonstrates how Hib inactivates and preserves ribosomes.
I have the following questions / comments about the work:
Questions/Comments:
1. The Supplementary Table 3 provides details for eight (8) maps while the maps & coordinates are provided for only seven (7). Also, the Supplementary Figure 14 shows local resolution for only seven (7) maps. ONE map is missing. Please provide the details for the missing map.
2. There is a big difference between masked (2.9 Å) and unmasked (4.5 Å) resolution in the Hib-CBSmodule-L1stalk map. The map also does not look like a 2.9 Å map and the Supplementary Figure 14 also highlights the same. Please recheck the mask used and comment on this difference.
3. How was SBDS identified in the complexes extracted from cell lysates?
Minor comments/changes/corrections:
Page 6 & 7: Text mentions Hib CTD adopts 'βαββα' fold whereas the figure legend (c) says 'βαβββα' topology in Hib CTD.
Page 6: Figure 1 (a) - In lane 3 band corresponding to Hib, what is the upper band that runs at the same level as PEP contamination in other lanes? Is this also PEP?
Page 6: Figure legend (e) starts with a double quotation mark (").
Page 7: The authors mention 'multi-stress' stationary phase. What does 'multi-stress' refer to?
Supplementary figure 4: Mention the colours for Hib and uS19 in the figure or in the figure legend.
Page 16: full-stop missing. "This SD:antiSD helix may protect the 16S rRNA from its 3' degradation as reported in bacteria"
Page 23: line 396 'demonstrate' in place of "demonstrates"
Supplementary Table 3: Information about reconstituted or complexes from cell lysates may be indicated to help the readers.

Tanweer Hussain
Indian Institute of Science
Bengaluru, INDIA

Reviewer #4

(Remarks to the Author)
I co-reviewed this manuscript with one of the reviewers who provided the listed reports. This is part of the Nature Communications initiative to facilitate training in peer review and to provide appropriate recognition for Early Career Researchers who co-review manuscripts.

Version 1:

Reviewer comments:

Reviewer #1

(Remarks to the Author)
The authors have revised the manuscript thoroughly and have addressed essentially all of the concerns raised. I have no further comments. However, I have one suggestion: in line 294, the reference to Fig. 6c is missing. In addition, I recommend indicating the gradient concentrations in Fig. 6c and displaying the polysome fraction, since the 30S, 50S, 70S, and polysome peaks together more accurately reflect the translational state.

Reviewer #2

(Remarks to the Author)
The authors have nicely revised their manuscript and I have no further comments and fully support the publication of this work.

Reviewer #3

(Remarks to the Author)
The authors have addressed all concerns and comments satisfactorily. The revised manuscript can be published as it is.

Reviewer #4

(Remarks to the Author)
I co-reviewed this manuscript with one of the reviewers who provided the listed reports. This is part of the Nature Communications initiative to facilitate training in peer review and to provide appropriate recognition for Early Career Researchers who co-review manuscripts.

Open Access This Peer Review File is licensed under a Creative Commons Attribution 4.0 International License, which permits use, sharing, adaptation, distribution and reproduction in any medium or format, as long as you give appropriate credit to the original author(s) and the source, provide a link to the Creative Commons license, and indicate if changes were made.
In cases where reviewers are anonymous, credit should be given to 'Anonymous Referee' and the source.
The images or other third party material in this Peer Review File are included in the article's Creative Commons license, unless indicated otherwise in a credit line to the material. If material is not included in the article's Creative Commons license and your intended use is not permitted by statutory regulation or exceeds the permitted use, you will need to obtain permission directly from the copyright holder.
To view a copy of this license, visit https://creativecommons.org/licenses/by/4.0/

Point by point responses to reviews – MS# NCOMMS-25-80265A

Reviewer #1 (Remarks to the Author):

This manuscript reports the identification of a novel archaeal ribosome hibernation factor, Hib, which structurally fuses an HPF/RaiA-like domain and CBS modules as in Dri. Through structural analysis, the authors describe the binding sites and putative functions of both the HPF/RaiA and CBS domains on the ribosome. Bioinformatic analyses further reveal the distribution and evolutionary relationships of hibernation factors across archaeal species.However, whether Hib truly represents “a new family” of hibernation factors should be carefully reconsidered. Structurally, the HPF/RaiA domain appears to play the dominant role, suggesting that Hib may instead constitute a subfamily of HPF/RaiA-like factors rather than a distinct family.Overall, the work represents a conceptual advance in the study of ribosome hibernation mechanisms, but several mechanistic claims require additional evidence and clarification.

We thank the reviewer for the positive assessment of our work.

Major comments

“However, whether Hib truly represents “a new family” of hibernation factors should be carefully reconsidered. Structurally, the HPF/RaiA domain appears to play the dominant role, suggesting that Hib may instead constitute a subfamily of HPF/RaiA-like factors rather than a distinct family.”

Thank you for this remark. However, the RaiA/HPF-like domain is not widespread in archaea (as shown in our phylogenetic analysis), whereas CBS modules are. Thus, the combination of these two domains, which leads to a HibA hibernation protein with four binding sites on the ribosome, appears to us as a new family of hibernation proteins, with a very specific mechanism. Nevertheless, we proposed to change the title of the manuscript into “A family of ribosome hibernation factors widespread in Archaea”

1.Origin and naming of “Hib”. The manuscript should explicitly state the origin of the name “Hib.” If it derives from “hibernation,” the term may be too informal, given that hibernation factors comprise diverse, structurally unrelated proteins. I strongly recommend reconsidering or justifying the choice of this name.

Considering its widespread distribution, we now refer to this archaeal protein family as **HibA** (for ribosome ***Hib***ernation factor in ***A***rchaea). This is now explained in the text lines 137-139.

2.The authors emphasize two specific insertions that interact with uS19 and uS12, suggesting they are important for Hib–ribosome binding. These interactions should be experimentally validated, e.g., by deleting the two insertions separately and testing whether Hib’s ribosome-binding ability is affected.

We agree with the reviewer that the mention of the archaeal-specific insertions in the C-terminal domain of HibA in the Introduction could over emphasize the importance of these insertions for

ribosome binding. Although these insertions do make contacts with the ribosome (described lines 152-159 and 425-438), there are many other contacts points (270 contacts in total, see Supplementary Data 1). Comparison of contacts between the archaeal and bacterial versions of RaiA/HPF domain is given in Supplementary fig. 3. This is why the description in the results section is more focused on the contact points that are archaeal-specific.

In the revised version, the two insertions are no longer cited in the introduction and only mentioned in the results section and the discussion. In addition, as suggested by the reviewer we produced a variant of HibA where the two insertions have been deleted (Δ_{283}SAL$_{285}$ and $_{324}$KFRGVHL$_{330}$ replaced by AA). To evaluate the impact of these two mutations we setup an *in vitro* translation assay based on luminescence detection (see methods). The results are now shown in fig. 6a and cited line 433-436. In this assay, the deleted version of HibA inhibits translation as efficiently as the wild-type version. This is in agreement with the very large number of contacts (270) and shows that the two insertions are not strictly required for HibA translation inhibition activity.

3.Line 195 describes interactions between β13 of Hib and the C-terminal tail of uS19 “extending the Hib β-sheet.” What is the reference for “extending”? In Fig. 3e–f, the β-strand of uS19 is not clearly visible—please mark both β13 of Hib and the β-strand of uS19.Similarly, line 410 states that “the first insertion also interacts with the C-terminal of uS19,” yet this is not labeled in Fig. 3d or Fig. S4. Please indicate the insertion clearly. The depiction of protein loops is too thick, making it difficult to discern secondary structures; consider refining these illustrations.

Thank you for your remark. Interactions between β13 of HibA and the C-terminal tail is described in Supplementary fig. 6 (previously Supplementary fig. 4). Supplementary fig.6 only is now cited line 160 (results section) and line 430 (discussion) to illustrate these contacts. The β strand of uS19 and HibA β13 are now labelled in Fig. 2c and in Supplementary fig. 6. In addition, the size of protein loops was reduced as suggested in Fig. 2 and 3.

The text has also been changed into “Other archaeal specific contacts are observed with the long C-terminal tail of uS19 (Supplementary fig. 6)” line 159, results section.

4.Hib’s N-terminal domain shows higher affinity for AMP than ATP, yet only ATP binding is observed in the structure. Does this imply that ribosome-bound Hib cannot bind AMP? If so, this could provide mechanistic insight into the regulation of Hib–ribosome interaction. Please discuss or experimentally address this discrepancy.

We apologize for the lack of clarity concerning this point in our first version. AMP and ATP are indeed observed in our reconstituted complexes prepared either in the presence of AMP or ATP. AMP binding was not illustrated in the first version but only briefly mentioned in the text. AMP binding is now more clearly explained in the text (line 258 “Although AMP was clearly visible in the cryo-EM map, we did not refine the model because of the limited resolution (Supplementary fig. 12). Nevertheless, the binding of AMP did not change classes conformation and distribution. This is explained in the text and illustrated in Fig5a and Supplementary fig. 12.

5.Both Hib and Dri contain CBS modules, but their ribosome-binding modes differ. It seems that

the PTC loop is critical for 50S interaction. The authors should compare the CBS–50S interfaces between Hib and Dri, identify conserved residues, and discuss shared structural principles.

Supplementary fig. 23 is dedicated to the comparison of the Hib and Dri proteins. We added two panels to better illustrate the binding sites of the HibA and Dri loops into the PTC (panels d and e). The stretch of conserved residues in Dri and HibA loops (blue parts in the figure) are located in two different areas of the ribosome.

Moreover, since CM-1, CM-2, CM-4, and CM-5 are proposed as potential hibernation factors, while CM-3 and CM-6 are not, the authors should analyze the conservation of these CM families relative to Hib and Dri to infer universal features of CBS modules as hibernation factors.

We analyzed sequence conservation in HibA-CM and reported the score of conservation on the 3D structure of HibA. The analysis shows that conservation areas in HibA-CM are located at the level of the nucleotide binding pockets and not at the interface between HibA and the ribosome. This is now shown on Supplementary fig. 22 and cited in the text lines 455-457.

It would also be important to test whether the N-terminal domain of Hib alone can mediate ribosome hibernation. Because Hib fuses CBS and HPF/RaiA domains, what is the functional advantage of this fusion? Truncation of the N- or C-terminal domains followed by ribosome-binding and cellular assays is recommended.

The N-ter domain of HibA was easily produced and purified. However, the C-terminal domain of HibA was only produced in the insoluble fraction. We tried to overcome this difficulty by constructing a version of HibA containing a thrombin site between the N and C domains. Unfortunately, the produced protein was not efficiently cleaved by thrombin.

The N-terminal domain of HibA was tested for its ability to inhibit translation in parallel to full HibA in our translation assay (Materials and methods). The analysis is now reported in the text lines 272-281 and in Fig. 6a.

In our conditions, the isolated N-terminal domain of HibA did not significantly inhibit translation as compared to full-HibA. This shows that the C-terminal domain is necessary for initial binding of HibA. This is consistent with (i) the fact that we never observed 50S alone bound to HibA although 30S HibA were observed (see Supplementary fig. 10 and text lines 242-244) (ii) the fact that the N-domain has multiple binding sites on the 50S. Therefore, the advantage of having a fusion between the two domains is to maintain the two subunits associated and to protect more vacant active sites on the ribosome. In addition, the three positions of the N-domain may not be possible in the absence of the C-terminal domain anchoring. These points are now mentioned in the discussion section lines 447-461. We thank the reviewer for pointing out the fact that the advantage of the fusion was not discussed before.

6.Is the “Hib-intermediate” state merely a transitional form between Hib-L1stalk and Hib-PTC? Given its relatively high proportion (51% in Fig. 1b), this seems unlikely. If it represents a distinct state, it should be named accordingly. Since the main conclusion—“Hib acts as a hibernation factor blocking all states of the dynamic translation process”—is based on the observation of three conformations, please discuss whether these states may instead result from weak N-domain–50S interactions. Moreover, what is the biological significance of blocking “all states” of translation?

We agree with the reviewer that the name was not appropriate. This state has been renamed HibA-uL5, as HibA interacts with uL5.

The three states of HibA are equally represented (Fig. 1). This may reflect that the accommodation of the N-domain on the 50S is driven by the accessibility of the binding sites and that the interactions only slightly contribute to the binding affinity. The three states collectively cover three active sites on the ribosome (PTC, uL5 and B2a bridge, and L1 stalk) and preserve them from degradation. Therefore, with only one protein, more active sites are protected thanks to the versatility of the N-terminal domain positions. This point (see also reviewer 2) is now discussed lines 437-461

7.While the study identifies potential interactions between Hib and several factors, the discussion of how these interactions mechanistically regulate ribosome hibernation is relatively limited. Expanding this section would strengthen the understanding of Hib-mediated regulation.

See reviewer 2.

The new organization of the manuscript has totally changed the presentation. aSBDS is now presented at the very beginning of the results. The participation of aSBDS and an SD:antiSD duplex in the hibernation process is also described in the results section in a paragraph entitled "multi-layered ribosome protection and inactivation" and discussed lines 488-501.

Minor comments

1.Fig. 1d: What "Δ517" refers to? It corresponds to ΔTERMP_00517. Either use "WT" in the legends or keep "Δ517" consistently in the figure, with a clear explanation.

WT correspond to ΔTERMP_00517, for clarity we termed this strain WT and mentioned parentality in materials (lines 568-569).

2.Ensure consistent use of "L1 stalk" (avoid mixing "L1 stalk" and "L1-stalk"). Similarly, standardize capitalization for "Hib" vs "HIB" and ribosomal protein names such as "uL1" (avoid "ul1").

This has been done throughout the manuscript.

3.When describing molecular interactions, include corresponding EM density maps to substantiate the claims.

Supplementary fig. 26 shows examples of the quality of the cryo-EM map around HibA protein binding sites. Unfortunately, it was not possible to display the cryo-EM maps in all illustrations for the sake of visibility. PDB files and cryo-EM maps are easily accessible to interested readers.

4.Fig. 3f: Label β13 directly in the figure to improve clarity.

This has been done in the corresponding figure of the revised version (Fig. 2c).

5.Extended Data Fig. 4: It would improve clarity if the meaning of different colors were indicated in the figure.

Extended Data Fig. 4 in the first version is now Supplementary fig. 8. This figure has been completely redrawn to simplify the message and labels have been added in the figure.

6.Please ensure all figure titles and legends accurately describe their figures. For instance, Fig. 1a contains an extra “)”,and similar errors appear elsewhere. Some legends are grammatically incorrect or missing punctuation (e.g., Fig. 5c: “Ribosomal proteinsThe N-terminal domain…”). Titles such as “Fig. 3: Hib-PTC” are overly simplistic and could be more descriptive.

Sorry about that. All figure legends have been carefully checked.

7.Avoid “data not shown.” Such expressions are discouraged in high-impact journals; either provide the data in supplementary materials or remove the statement.

“data not shown.” has been removed. A new panel in Supplementary fig. 11 showing the curves recorded upon GMP and GTP titrations has been added.

8.Extended Data Fig. 9a: Keep “Nmodule” as “N-module”, “CBSdomain” as “CBS domain”. Use either “Active loop” or “PTC loop”, it is not necessary to use two names in the figure. Change “Comparison of views b. and c.” to “xxxx b and c”.

The corrections have been made and PTC loop is now used in the figures and in the text.

9.Fig. 6a: Change the title from “Distribution of Hib, Hib-Cter, …” to “Distribution of Hib, HPF/RaiA, …”.

The corrections have been made.

Reviewer #2 (Remarks to the Author):

In the present manuscript, Clement Madru and colleagues make an outstanding discovery of a novel ribosome hibernation factor, Hib. They combine structural, genetic and biochemical characterization of Hib to demonstrate that this protein initiates ribosome hibernation and improves environmental stress tolerance in the archaeon Pyrococcus abyssi. They then provide a comprehensive evolutionary analysis to illustrate that Hib likely acts as a hibernation factor not only in their model organism but in nearly a half of studied archaeal species.

The importance of this finding is difficult to overstate. As you certainly know, ribosomes are the most metabolically expensive molecules in living cells: they account for nearly a third of a cell's dry mass and require more cellular energy for their production (60%) than all other cellular components combined! Therefore, it is not surprising that all studied species utilize a sophisticated mechanism—ribosome hibernation—for safely storing their ribosomes during starvation and stress. When cells lack hibernation factors, they can no longer recover from prolonged periods of stress.

Currently, the archaeal domain remains one of the least studied branches of life with regard to ribosome hibernation—so that its mechanisms in many archaeal species remain completely unknown. This study fills critical gaps in our knowledge by revealing several conceptually novel

properties of hibernation factors, both in Archaea specifically and for the field in general.

Below, I provide my suggestions for how to improve this study, specifically its presentation. I want to stress, however, that—given the novelty and impressive amount of work,—I believe that this paper can be accepted and published as is. My main suggestions are related to how the data are presented to give the authors better words to explain the importance of this outstanding work to a broader readership.

We are very grateful to the reviewer for the positive and constructive assessment of our work. We have carefully considered all suggestions regarding the presentation of the manuscript and have substantially revised the Abstract, Introduction, Results and Discussion sections accordingly. In particular, we now (i) highlight more explicitly the main conceptual advances of the study (discovery of HibA, its conformational plasticity on the ribosome, and the taxonomic/evolutionary insights); (ii) change the presentation of the Results to first present the native HibA–ribosome complexes and their conformations, then the genetic and physiological evidence that HibA preserves 70S ribosomes and supports recovery from stress, and finally the taxonomic and evolutionary analyses; and (iii) introduce aSBDS at the very beginning of the Results.

We fully agree with the reviewer that the frequent co-occurrence of aSBDS with HibA on idle ribosomes is intriguing and may point to additional layers of ribosome protection. Considering the novelty of the identification of HibA, the article is focused on this protein. aSBDS is presented as s a possible factor that can complement HibA in protecting inactive ribosomes under stress. 3D classification based on aSBDS was deepened and the percentage of aSBDS-containing classes was updated accordingly. In addition, an MS analysis of the 70S sample used in our cryo-EM analysis (shown in Fig. 1 in the revised version) has been performed and is now cited at the end of the first paragraph of the results section and presented in Supplementary fig. 4.

ABSTRACT

Their abstract would certainly benefit from mentioning some of their conceptually novel findings that set this work apart from every other study of ribosome hibernation.

1. Firstly, they show that the archaeal cells of Pyrococcus abyssi initiate two parallel ribosome hibernation pathways, mediated by the previously uncharacterized proteins Hib and SBDS. While they do not have high resolution structures or genetic studies supporting the role of SBDS protein in ribosome hibernation, their Figure 2 shows that this project is a major binder of idle ribosomes in stressed archaea. They could certainly mention this fact.
The abstract has been re-written accordingly.

2. Secondly, they understate that their structures demonstrate how a single ribosome hibernation factor, Hib, may adopt several distinct conformation in the ribosome. This finding illustrates that our somewhat naïve expectation of “single complex = single structure” may lead to failure to understand how ribosome hibernation factors work and how they can be discovered using cryo-EM.

This point is now highlighted in the abstract.

3. Thirdly, the authors demonstrate that many archaeal species possess not a single but multiple hibernation factors simultaneously. This indicates that the identification of a single factor in a

given species does not reveal the full repertoire of players involved in this essential process in a given species.

We thank the reviewer for this comment. In *Pyrococcus abyssi*, ribosome hibernation may involve complementary ribosome-associated mechanisms, with HibA acting as the primary factor and aSBDS, deacylated tRNAs and an SD:antiSD duplex potentially contributing to ribosome protection, whereas taxonomic analyses indicate that canonical hibernation factors are generally mutually exclusive across archaeal lineages. We have clarified this point in the Abstract.

TEXT AND FIGURES

I would rewrite this story completely to simplify the message and better explain the importance of these findings:

1. Ribosome hibernation in the archaeon Pyrococcus abyssi relies on two uncharacterized factors, Hib and SBDS.

First, I would present the data from the original Figure 2 as the new Figure 1 to immediately establish the paper's main conclusion. This figure would be modified, specifically panel C, to categorize the ribosomes as "hibernating-like" and "translating-like" rather than "Hib-bound" and "Hib-free," as this more accurately describes their functional states.

We thank very much the reviewer for his suggestions that improved the presentation of our results. We now start with the presentation of the data from the original figure 2 (70S preparation in near-native state that becomes Fig. 1 in the revised version) as suggested, and follow the plan proposed by the reviewer. we also categorized the ribosomes as "hibernating-like" and "translating-like" as suggested.

Here, I want to stress that their present description of SBDS confuses readers a little bit: this protein visually resemble EF-G/eEF-1A and it is only vaguely mentioned in the results—despite being bound to essentially a third of ribosomes in stress Pyrococcus abyssi. The authors could move, for instance, the discussion of this protein from their Discussion section to the first section of the Results.

We apologize if our text was not clear but we do not think that SBDS resembles "EF-G/eEF-1A," (only domain 3 of SBDS resembles domain V of EF-G). We only wanted to note that the position of the C-terminal domain of aSBDS occupied a position similar to that occupied by the tRNA elbow during the first step of accommodation when an aminoacyl-tRNA:EF1A complex is bound to the ribosome (lines 220-224, illustrated in Supplementary fig. 9a and c). As suggested by the reviewer, aSBDS is now presented at the very beginning of the results section and part of the discussion concerning this protein has been moved.

2. Hib adopts multiple distinct conformations on the ribosome.

I would then present the ribosome/Hib structures (original Figure 1) to illustrate that Hib undergoes a conformational plasticity that is unique among known hibernation factors. This finding has critical implications for how cryo-EM data of hibernating ribosomes should be processed and for the discovery of novel hibernation factors that may exhibit similar heterogeneity.

Thank you for highlighting this point, which is indeed an important new development in our work. The second paragraph of the results is now entitled as suggested and the conformational plasticity of HibA is also highlighted in the discussion (lines 437-461).

3. Hib stabilizes ribosomal subunit interactions and enhances cellular stress tolerance. Next, I would present on subunit association and cell viability (e.g., original Figure 1 d-e) to demonstrate that Hib stabilizes ribosomes in the 70S form—a state associated with higher stability of ribosomes in non-dividing cells [PMID: 34253058, PMID: 32123037]—and thereby bolsters the archaeal cells' tolerance to stress.

This has been done accordingly.

On a minor note, could the authors clarify their figure caption (Fig. 1d,e) where they state that the blue line corresponds to the Delta517 strain? Is Delta517 the same as the wild type? Also, I slightly disagree with their interpretation of the growth recovery experiments, where they state that the difference between the two strains reflects the differences in their growth rate, not mortality. In fact, their Supplementary Fig. 3 does show that Hib deletion appears to decrease the viability of cells after 20-120 h of stress.

WT correspond to ΔTERMP_00517 (Delta517 strain), for clarity we termed this strain WT and mentioned parentality in materials (lines 568-569).

We have clarified the presentation of the growth and viability data in the Results section.

First, we agree that the ΔhibA strain displays a modest decrease in viability during prolonged nutrient deprivation. We have therefore revised the text to state that ΔhibA shows a slightly greater loss of viability during the first 20 h and a modestly lower viability up to 120 h (Supplementary fig. 14). This revised wording more accurately reflects the data and avoids implying that viability is strictly identical between the strains.

Second, we have clarified that the exponential growth rates of WT and ΔhibA are similar, as shown by the parallel slopes of the exponential phase in Fig. 6b. Thus, the observed phenotype corresponds to an extended lag phase rather than a difference in growth rate.

4. Hib is a highly conserved archaeal protein.
I would then present their data on Hib occurrence in Archaea to show that this protein is present in nearly a half of studied archaeal species.

This has been done accordingly.

5. Archaeal species appear to possess a highly redundant machinery of ribosome hibernation. And I would finish the manuscript by explicitly stating another conceptually novel finding of their work: that many species that contain Hib have been recently characterized to bear other hibernation factors, such as Dri, aRDF and mRDF component (see, for instance, this paper: PMID: 40914243)

We agree that archaeal ribosome hibernation involves a broader diversity of factors than previously anticipated. In the revised manuscript, we now incorporate the recently described mRDF complex (PurH–aEF2) into the Introduction & Results (lines 75-80 & 310-314). This addition strengthens the presentation of the emerging functional diversity of archaeal hibernation mechanisms. Given that

our Discussion already focuses on mechanistic and evolutionary aspects of Hib and CBS-based factors, and that the phylogenetic patterns point to mutual exclusion rather than widespread redundancy, we chose not to duplicate this information there.

6. I would move all other figures—including Figures 3, 4a-b, Figure 5—to the supplementary information, as they are highly technical and do not substantially advance understanding of Hib's biology. This is because details such as hydrogen bonds and salt bridges are self-evident from the structures and are likely of interest only to a specialized audience.

Following the reviewer recommendation we carefully reviewed all our figures. As suggested, Fig. 1 is now dedicated to the presentation of our analysis in cell extracts. Fig. 2 now presents HibA protein and the binding site of the C-terminal domain. The new version of Fig. 3 was produced by merging the less detailed views from previous Fig3 and Fig4.Therefore the three conformations are now shown on a same figure, highlighting their importance. Fig. 4 is focused on SBDS and SD :antiSD. All other detailed views have been moved in Supplementary data. We think that the new versions of the figures clarify the information and we thank the reviewer for his suggestions.

I would like to stress, however, that this feedback is intended solely to improve the manuscript's presentation; it does not diminish the outstanding contribution of this study to our understanding of how living cells remain viable under stress through dormancy.

Sincerely,
Sergey Melnikov

Reviewer #3 (Remarks to the Author):

This manuscript by Madru et al. deals with the identification of Hib, a family of hibernation factors in Archaea. The authors show that Hib is an important hibernation factor in Archaea using genetic, structural and biochemical studies and computational analysis. Hib inactivates ribosomes by blocking mRNA channel and all three tRNA binding (A, P and E) sites. Deletion of hib led to decrease in 70S ribosome pools and delay in recovery from stationary phase.
The authors have obtained high-resolution cryo-EM structures of complexes of Hib with archaeal ribosomes and resolved three conformations of Hib blocking A, P and E sites. They propose that Hib acts as a hibernation factor by blocking all states of the dynamic translation process and preserve the ribosome from dissociation and degradation. In Hib:ribosome complexes extracted from archaeal cells E-site tRNA was observed, which may serve as an aa-tRNA deprivation sensor for Hib hibernation factor. Interestingly, hibernating ribosomes also contain Shine-Dalgarno (SD):antiSD duplex although, the origin and significance of this Shine-Dalgarno (SD):antiSD duplex is not clear. Overall, this study identifies a new family of hibernation factors in Archaea and demonstrates how Hib inactivates and preserves ribosomes.
I have the following questions / comments about the work:

We thank the reviewer for the positive assessment of our work.

Questions/Comments:

1. The Supplementary Table 3 provides details for eight (8) maps while the maps & coordinates are provided for only seven (7). Also, the Supplementary Figure 14 shows local resolution for only seven (7) maps. ONE map is missing. Please provide the details for the missing map.

We apologize for the inconvenience. Obtaining the PDB code for the eighth file took some time due to a bug in the submission system. All PDB and EMDB numbers are now listed in Supplementary Table 3.

In addition, local resolution map for HibA-ATP has been added in Supplementary fig. 13. We thank the reviewer for raising this point.

Supplementary Figure 14
2. There is a big difference between masked (2.9 Å) and unmasked (4.5 Å) resolution in the Hib-CBSmodule-L1stalk map. The map also does not look like a 2.9 Å map and the Supplementary Figure 14 also highlights the same. Please recheck the mask used and comment on this difference.

The large difference between the masked and unmasked FSC curves arises from the particle extraction strategy used for the signal-subtracted dataset. We intentionally kept the same box size as for the other maps in order to ensure proper alignment between all models and reconstructions. As a result, a large amount of noise remains in the region previously occupied by the ribosome, which strongly impacts the unmasked FSC estimation (see below).

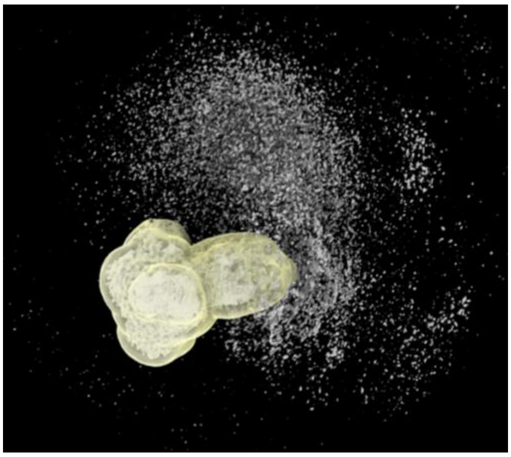

In addition, we have re-examined the mask used for the FSC calculation and confirm that it is appropriate, with good coverage of the signal and a sufficiently soft edge, making an artificial inflation of the resolution unlikely. Although the masked FSC yields a nominal global resolution of 2.9 Å, we agree with the reviewer that this value does not reflect the visual appearance of the entire map. The map shows good-quality in the well-ordered regions, while the local resolution is clearly lower at the base of the L1 stalk and in other flexible regions. This heterogeneity is also visible in Supplementary Fig. 24, where the map is displayed with a relatively broad contour, leading to a wide outline that emphasizes the less well-resolved regions. We provide below additional views of the local resolution map shown at different contour levels. These views illustrate that, despite the presence of flexible regions, the core of the map reaches local resolution values consistent with the reported masked FSC of ~2.9 Å.

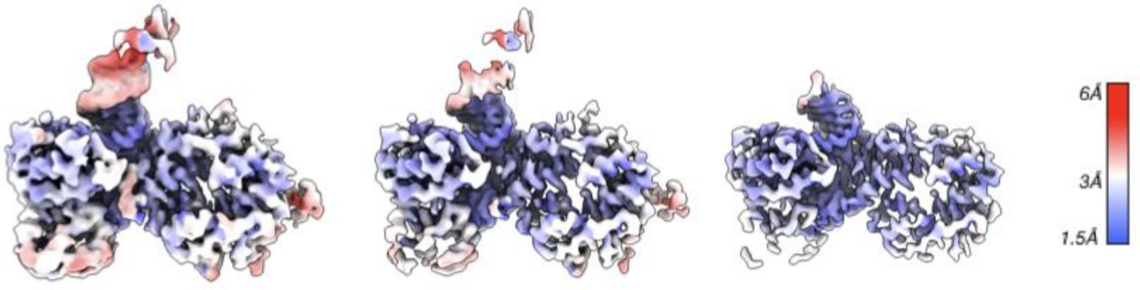

3. How was SBDS identified in the complexes extracted from cell lysates?

SBDS was identified on the basis of the interpretation of the cryo-EM map where secondary structures are visible (EMDB-55136). In addition, we have now performed MS analysis on our 70S sample preparation and observed that SBDS and HibA are as abundant as ribosomal proteins. This analysis is now reported in the manuscript and in supplementary fig. 4.

Lines 145-148: "Importantly, mass spectrometry analysis of our 70S fraction (Fig.1A) showed that HibA and aSBDS are indeed detected in our sample and have iBAQ values similar to those of ribosomal proteins, which supports their high abundance (Supplementary fig. 4)".

Minor comments/changes/corrections:

Page 6 & 7: Text mentions Hib CTD adopts 'βαββα' fold whereas the figure legend (c) says 'βαβββα' topology in Hib CTD.

Thank you for pointing this out. 'βαββα' fold has been replaced everywhere by 'βαβββα'.

Page 6: Figure 1 (a) - In lane 3 band corresponding to Hib, what is the upper band that runs at the same level as PEP contamination in other lanes? Is this also PEP?

The SDS-PAGE analysis that was shown in Figure 1 of the first version of the manuscript is now presented in Supplementary fig. 10. The upper band in lane 3 is a contaminant in HibA preparation. This is now mentioned in the legend of the figure.

Page 6: Figure legend (e) starts with a double quotation mark (").

This has been corrected.

Page 7: The authors mention 'multi-stress' stationary phase. What does 'multi-stress' refer to?

We clarify in the text. Lines 294-297, where "multi-stress" stationary phase is now defined as the simultaneous exposure to several physiological constraints associated with stationary phase, including nutrient limitation and physicochemical stresses (accumulation of metabolic by-products, associated pH changes, and alterations in ionic and redox conditions of the culture medium).

Supplementary figure 4: Mention the colours for Hib and uS19 in the figure or in the figure legend.

Sorry about that. The colors are now defined in the figure and the legend.

Page 16: full-stop missing. “This SD:antiSD helix may protect the 16S rRNA from its 3’ degradation as reported in bacteria”

This has been corrected.

Page 23: line 396 ‘demonstrate’ in place of “demonstrates”

This has been corrected.

Supplementary Table 3: Information about reconstituted or complexes from cell lysates may be indicated to help the readers.

We now mention in the title of Supplementary Table 3 that the cryo-EM analysis was done on ribosomal complexes isolated from cell lysate.

Tanweer Hussain
Indian Institute of Science
Bengaluru, INDIA

REVIEWERS' COMMENTS

Reviewer #1 (Remarks to the Author):

The authors have revised the manuscript thoroughly and have addressed essentially all of the concerns raised. I have no further comments. However, I have one suggestion: in line 294, the reference to Fig. 6c is missing. In addition, I recommend indicating the gradient concentrations in Fig. 6c and displaying the polysome fraction, since the 30S, 50S, 70S, and polysome peaks together more accurately reflect the translational state.

We added the reference to Fig. 6c in the text. We also indicated gradient concentrations and polysomes fractions in Fig. 6c

Reviewer #2 (Remarks to the Author):

The authors have nicely revised their manuscript and I have no further comments and fully support the publication of this work.

Reviewer #3 (Remarks to the Author):

The authors have addressed all concerns and comments satisfactorily. The revised manuscript can be published as it is.

Reviewer #4 (Remarks to the Author):

I co-reviewed this manuscript with one of the reviewers who provided the listed reports. This is part of the Nature Communications initiative to facilitate training in peer review and to provide appropriate recognition for Early Career Researchers who co-review manuscripts.
